# A Computer Model of Oxygen Dynamics in the Cortex of the Rat Kidney at the Cell-Tissue Level

**DOI:** 10.3390/ijms20246246

**Published:** 2019-12-11

**Authors:** Vivien Aubert, Jacques Kaminski, François Guillaud, Thierry Hauet, Patrick Hannaert

**Affiliations:** INSERM U1082-IRTOMIT, 86000 Poitiers, France; aubert_vivien@yahoo.fr (V.A.); jacques.kaminski@gmail.com (J.K.); fguillaud@gmail.com (F.G.); thierry.hauet@gmail.com (T.H.)

**Keywords:** kidney, oxygen, renal cortex, peritubular capillary, proximal tubule, rat, hemoglobin, oxygen consumption, Na reabsorption, agent-based model

## Abstract

The renal cortex drives renal function. Hypoxia/reoxygenation are primary factors in ischemia-reperfusion (IR) injuries, but renal oxygenation per se is complex and awaits full elucidation. Few mathematical models address this issue: none captures cortical tissue heterogeneity. Using agent-based modeling, we develop the first model of cortical oxygenation at the cell-tissue level (RCM), based on first principles and careful bibliographical analysis. Entirely parameterized with Rat data, RCM is a morphometrically equivalent 2D-slice of cortical tissue, featuring peritubular capillaries (PTC), tubules and interstitium. It implements hemoglobin/O_2_ binding-release, oxygen diffusion, and consumption, as well as capillary and tubular flows. Inputs are renal blood flow RBF and PO_2_ feeds; output is average tissue PO_2_ (tPO_2_). After verification and sensitivity analysis, RCM was validated at steady-state (tPO_2_ 37.7 ± 2.2 vs. 36.9 ± 6 mmHg) and under transients (ischemic oxygen half-time: 4.5 ± 2.5 vs. 2.3 ± 0.5 s in situ). Simulations confirm that PO_2_ is largely independent of RBF, except at low values. They suggest that, at least in the proximal tubule, the luminal flow dominantly contributes to oxygen delivery, while the contribution of capillaries increases under partial ischemia. Before addressing IR-induced injuries, upcoming developments include ATP production, adaptation to minutes–hours scale, and segmental and regional specification.

## 1. Introduction

Renal hypoxia is considered a common root of acute and chronic kidney diseases [1,2]. In transplantation, the unavoidable ischemia-reperfusion sequence (IR) is involved in short and long-term graft dysfunctions and injuries (IRI) [3,4,5]. Paradoxically, the kidney can sustain hypoxic/ischemic periods up to tens of hours [6], but it is also exquisitely sensitive to oxygenation/perfusion defaults [7,8], to the point that ischemia-induced renal failure is much more frequent than in other organs [9]. Renal oxygenation has been puzzling renal physiologists for decades, and still does [1,7,10,11]. Sixty years after the first evidence of oxygen decrease from the cortex to the inner medulla, numerous features have been experimentally unraveled [11,12]. In addition to the cortico-medullary PO_2_ gradient (40–60 mmHg in the cortex to 5–25 mmHg in the medulla), they include the arteriovenous (AV) diffusive oxygen shunting, a low oxygen extraction ratio (10–15%), a luxurious perfusion amounting to 10% of cardiac output per kidney—required for an efficient filtration function—and an oxygen consumption driven by glomerular filtration: the more it filters, the more oxygen the kidney consumes for Na^+^ reabsorption [13,14] (for a quantitative review of basal oxygen consumption, see [15]). Moreover, the kidney exhibits intricate anatomical and metabolic specificities, at the regional and segmental levels [16,17], coupled to a complicated glomerulo-tubulo-vascular organization and regulations [18], which altogether appear to dictate tissue-level oxygen-related heterogeneities [19]. This complexity pushed physiologists to resort to mathematical modeling and computer simulation (MS), as a complement to the experimental approaches [10,20,21,22,23].

Renal oxygen distribution started to be addressed under the MS angle in the late 90’s [12,20]. The AV oxygen shunt has been long hinted and its mathematical descriptions are based on careful anatomic determinations. However, its physiological relevance and quantification remain debated [23,24,25,26]. Finally, oxygen distribution within the medulla, with its countercurrent system and complex organization, has been modeled with attention and a great level of histological detail [27,28,29,30].

Conversely, oxygen distribution in the cortex has been addressed only lately, in the mid-2010’s. Likely, the urine concentration and NaCl reabsorption role of the medulla have somewhat eclipsed the cortex [30,31]. Further, since the cortex is highly perfused and exhibits PO_2_ levels similar to other organs’ (30–60 mmHg), its oxygenation has been considered unlimited. Finally, the cortical apparent disorder (cortical tubular labyrinth, entangled with the peritubular capillary network), is less amenable to geometric treatment than the highly organized medullary regions [32].

Recently though, a few modeling studies of oxygen use and distribution in the renal cortex have been proposed. These MS studies follow either one of two conceptual lines: one focuses on nephron segmentation and epithelial polarity and transport, while the other ignores epithelial “details” but integrates vascular anatomical features to describe delivery and consumption all along the renal vascular tree. In the tubule-centered line, expanding the proximal constructs by Weinstein and et al. [33,34], Layton and et al. provide a detailed description, along the proximal tubule, of oxygen consumption in relation to epithelial transporters [22,35]. However, they ignore both tissue heterogeneity and oxygen availability. Conversely, in the vascular-centered line, Lee and et al. propose an elaborate model of oxygen transport and consumption in the rat renal cortex, based upon a previous model of AV diffusional shunting (see above). This top-down anatomically-based model predicts average tissue and micro-vascular PO_2_ [10].

From a pathophysiological standpoint, renal IRI has been shown to develop in specific areas, such as the deep cortex, and tissue regions in the kidney, tubular segments, and cell-types exhibit marked differences in terms of lesional or adaptive responses to alterations in oxygen levels [8,36,37]. Moreover, the ultimate sensors and victims of ischemia and/or reperfusion are parenchymal and mesenchymal cells, including epithelial, capillary, and interstitial cells, as reflections of renal tissue heterogeneity. Thus, an informed description of the mechanisms and specificities of renal cortical oxygenation at the histological level under normal conditions appears as a minimal prerequisite to understand, quantify, and ultimately to correct the central role of oxygen-related dysfunctions. However, this is bound to be an ab initio endeavor, since no MS study at the cortical cell-issue level has been proposed yet.

The objective of the study is as follows., As a first step toward a realistic description of kidney IR events, we present here the development and validation of a computer model of renal cortical oxygenation, at the cell-tissue level. In order to address the discreteness and heterogeneity of cellular, tubular and tissue components, we use agent-based modeling. In order to assure internal consistency and to rule out inter-species differences, the model is specifically parameterized for the Rat. Including the main biophysical features of oxygen transport, exchange, diffusion, and consumption, within an idealized histological tissue slice, it is a dynamic construct, able to delineate transients as well as steady-state PO_2_.

## 2. Methods

### 2.1. Bibliographical Analysis and Data Extraction

A thorough bibliographical search and analysis was performed for the extraction of Rat kidney data (anatomical and geometrical, functional and biochemical), quantitative and qualitative. We focused on healthy, adult male Rats ~70%, the rest female); only studies/data relating to animals older than 12 weeks (or with a bodyweight >180 g) were considered. More than 800 Rat-related references were surveyed and stored (PubMed©, BiblioInserm©, BiblioVie©/CNRS, GoogleScholar©, QKDB), and about 160 original were selected and used. The species targeted in the study is the Rat (*Rattus norvegicus*), which in our experimental studies corpus (kidney, cortex, tubule, flow) is dominated by the Sprague-Dawley and Wistar strains (about half of the studies), followed by the Münich (Münich–Wistar and Münich Frömter), Dahl, Lewis and Wistar–Kyoto strains. For simplicity and because the different parameters required by the model are not available for all those strains, no attempts were made to distinguish between those strains.

### 2.2. Agent-Based Modeling with NetLogo©

Histological elements such as tubules, capillaries, and cells are discrete and discontinuous elements. As opposed to more conventional equation-based formalism [27,28,33], they are best described by the computer “agent” concept, as implemented in agent-based modeling, ABM [38,39]. Agents are autonomous, “reactive” objects, dynamically interacting with each other, according to a set of rules. They are defined by variables and functions which allow them, at each execution step, to modify their own variables as well as those of other agents, including those constituting their environment. We used NetLogo© (version 6.10), a free ABM programming language and modeling platform, which presents multiple advantages, including power and versatility. Originally designed for educational purposes, NetLogo is now widely used for research as well. For detailed presentations of ABM and NetLogo, see [38,39,40].

In NetLogo, patches are a special type of agent that is fixed, unable to move, but otherwise, possess all agent properties. Since tissue structure is fixed in our rat renal cortical model (RCM), we use “patches”: thus, we will use “patch” to refer to model elements.

### 2.3. The Cortical Tissue Model for the Rat Kidney

**Figure 1:**
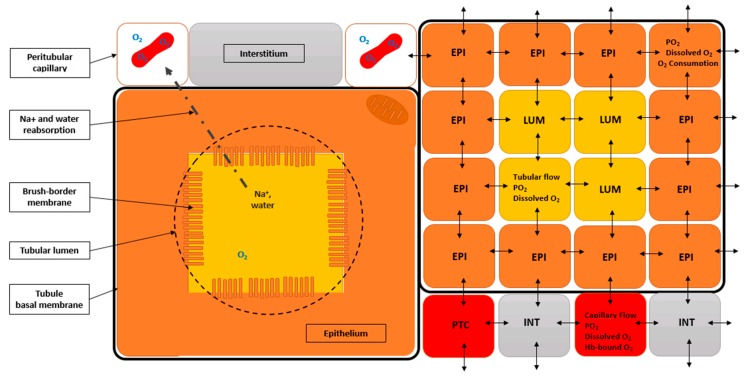
Schematic illustration of renal cortical tissue in rat renal cortical model (RCM). (**Left**) Histological components that are implemented in the model. Peritubular capillaries (white squares), which contain red blood cells, oxygen, and hemoglobin (Hb). Tubular epithelium (orange square), with its brush-border membrane; nuclei are not represented, but one O_2_-consuming/ATP-producing mitochondria is symbolized in the upper right corner. Tubular lumen (yellow square), containing glomerular ultrafiltrate (including water, sodium, oxygen); the point-dotted arrow symbolizes tubular reabsorption from the lumen to one peritubular capillary, an ATP/O_2_ consuming process. Interstitium (grey rectangle): only “fixed” elements are considered in the current model, thus interstitial cellular elements, such as resident fibroblasts and macrophages, are not represented, although their oxygen consumption is accounted for in model calculations. The square thick black line represents the tubule basal membrane. (**Right**) RCM tissue implementation with NetLogo. This section shows how the histological components are mapped within NetLogo, the ABM software used (nb: we use only fixed agents, called “patches” in NetLogo). Four different types of patches are defined: (i) peritubular capillaries (PTC), or peritubular capillary patches, characterized by their flow rate (transverse, not represented), PO_2_, Hb content, oxygen content, and concentration, etc (erythrocytes are not represented and hemoglobin is considered homogeneously distributed within PTC); (ii) epithelial patches (EPI), or tubular epithelial patches, characterized by similar O_2_-related variables (no Hb, no flow), (iii) luminal patches (LUM), or luminal patches (flow rate, O_2_-related variables, no Hb, no consumption), and (iv) INT, or interstitial patches (same variable types as EPI). Importantly, all patch-types have their own oxygen diffusion coefficient: double-headed arrows symbolize inter-patches oxygen diffusive exchanges.

The RCM is an idealized slab of the cortex. Figure 1 illustrates the mapping of the four principal cortical histological components into NetLogo, using fixed agents, the so-called “patches” (mobile cell elements such as macrophages are currently not represented). Vascular patches (PTC), correspond to peritubular capillaries, including their endothelial layer (not represented, because typical height is <1.0 µm). PTC were axially perfused with post-glomerular, capillary “blood”. Note that erythrocytes are not individualized, but idealized as “mixed” with the plasma. Model tissue receives oxygen input via “blood” perfusion of PTC patches. Epithelial patches (EPI) roughly correspond to proximal cells, and their various cell surfaces (brush-border, lateral, basal) are attributed values from detailed morphometric determinations [41,42]. Luminal patches (LUM) form the tubule lumen. One important feature of RCM, overlooked in other studies, is the implementation of such tubular patches and related perfusion. Interstitial patches (INT) correspond to the mesenchymatous interstitium, extracellular matrix, and interstitial cells.

RCM tissue dimensions are 320 µm × 320 µm, subdivided into 1024 square patches (10 × 10 µm^2^). In order to define oxygen concentration, exchange surfaces, as well as orthogonal capillary and tubular feeding volumetric flows, a thickness of 10 µm was chosen, close to epithelial cells and luminal dimensions (default patch volume: 1000 µm^3^, or fL). Total tissue volume is ~1.0 nL (1024 fL). Additional simplificationse: (i) brush-border height isaggregated to the lumen (BBH° = 4.3 µm, SM1-Table S3a); (ii) glomerules (4% *v*/*v* of the cortical tissue) are considered inexistent at RCM scale (in-between two cortical radial arteries and their glomerular tufts); (iii) non-capillary vessels (~15% *v*/*v*) are ignored, as well as lymphatics (2% *v*/*v*; [41,43]).

Patches and model variables: patches possess their own variables, including oxygen diffusivity D (patch units, PU: µ^2^/ms), volume (µ^3^ or fL), oxygen content (amoles), oxygen concentration (amol/patch, or µmol/L) and oxygen partial pressure (mmHg), in addition to house-keeping (all patches) and transport-related oxygen consumption (epithelial patches only). Model and patches also handle global variables, including the number of nephrons, volumetric flows (e.g., RBF and single-nephron glomerular filtration rate, SNGFR).

Model structure and geometry: from bibliographical analysis and histological slices from the laboratory, we devised several “cortical tissues”: their detailed characteristics are presented in Supplementary Materials (SM2-Table S1). The main morphometric features are the capillary density and the proximal tubule dimensions, both expected to influence oxygen supply and consumption. The outer tubular radius (orTub) ranges 15–30 µm (see SM1-Table S3a), and tubules represent ~60–80% *v*/*v* of the cortex. The capillary density ranges 250–1110 mm^−2^ (capillary/tubule ratio ranges 1.1–2.2)

In order to quantify the influence of these indexes on model output, we constructed six tissue variants for RCM, featuring two radius (orTub = 20 µm or 25 µm), combined with three capillary/tubule ratio (Nc/Nt ratio = 1.0, 1.5 and 2.0): the reference, base-case tissue is the “1540”, for 1.5 Nc/Nt and 40 µm orTub. Model tissues were edited using a customized NetLogo program (a modification by one of us, VA, of the “NetLogo Pac-Man Level Editor”, U. Wilenski (2003), available in NetLogo Models Library).

Finally, for proper oxygen diffusion (Fick’s first diffusion law), the effective exchange surface area (ESA) is a key morphometric aspect: e.g., the brush-border membrane for lumen-epithelial contacts augments considerably (~20-fold) the lumen-to-epithelium contacts [41]. Because ESA varies according to tissue components involved, we defined adjusting factors for all exchanging patch-type pairs in the diffusion routine: the default ESA (100 µm^2^) is multiplied the corresponding patch-to-patch factor (Equation (16); see SM1-Table S3b).

### 2.4. Units, Inputs/Outputs, Parameters and Variables

Units and normalization: for the whole kidney, we normalize eligible variables to gram of kidney weight (gkw), whereas in RCM we express most variables either *per* nephron (e.g., volumetric flows in nL/min), or per patch type. For O_2_ consumption, we equate “mmol. (min.10^3^ gkw)^−1^ “ to “mM/min” (or mmol.(L.min) ^−1^), considering kidney tissue density equal to 1 mL/gkw [42]. Further, because we address either the whole-kidney, the cortex itself, or patches within the model, in order to disambiguate, we add a label (with a hyphen) to “mM”: for instance “mM-wk/min” for the whole kidney or “mM-ctx/min” for the cortex. For patches, we refer to their “specific” consumption (e.g., mM-EPI/min). Within RCM, units are adapted to patch and time dimensions (patch units, PU): µm, ms, amol and mmHg (1 amole/1000-µm^3^ patch = 1 µmol/L or µM).

Model inputs/outputs, parameters, and variables: model inputs are external (or independent) variables that drive model outputs, given a parametric configuration. The main model inputs are renal blood flow (RBF) and feeding PO_2_ (capillaries and tubules). Inputs can be fixed for the entire simulation, or changed at run-time. Model outputs are dependent variables; the main output is the (average) cortical oxygen partial pressure, because experimentally it is the measured variable (in some studies, the vascular PO_2_ is determined). As mentioned, each patch has its own set of variables, among which O_2_ amount is the state variable. This allows for monitoring local and average PO_2_ for epithelial, capillary, interstitial, or luminal compartments. Finally, we define independent parameters, the value of which conditions other (dependent) parameters.

### 2.5. Model Equations

We implemented dynamic descriptions of perfusion, hemoglobin-O_2_ binding/release, diffusion, and consumption, in order to determine levels of oxygen throughout the model and the different patch types.

#### 2.5.1. Equations for Perfusion and Volumic Flows

(1) “Cortical blood flow:
  (1) CBF=RBF .frCBF
  where CBF, model cortical blood flow (mL.min^−1^.gkw^−1^; FU, flow units), RBF, renal blood flow: FU (model input, controlled from the model interface), frCBF, fractional cortical blood flow (see text, above; no unit).
(2) Operational nephrons:
  (2) $$\mathrm{OpNe}=N_{\mathrm{tot}}\;.\mathrm{frNCtx}$$
  where OpNe is the functioning nephron density: gkw^−1^, N_tot_ is total nephron density in the kidney: gkw^−1^, frNCtx is fraction of operational nephrons in the model(no unit).
(3) Single nephron glomerular blood flow:
  (3) $$\mathrm{SNGBF}=10^{6}\;.\frac{\mathrm{CBF}}{\mathrm{OpNe}}$$
  where SNGBF is single nephron glomerular blood flow (nL/min; 10^6^ converts mL to nL). Please note that the flow unit now refer to a single nephron.
(4) Single nephron efferent arteriolar blood flow:
  (4) SNEABF=SNGBF .[1−FF .(1−Hta)]
  where SNEABF is the single nephron efferent arteriolar blood flow (nL/min), FF is the filtration fraction: no unit, Hta is the arterial hematocrit (no unit).
(5) Single nephron glomerular blood flow,
  (5) SNGFR=SNGBF−SNEABF
  where SNGFR is the single nephron glomerular blood flow (nL/min).
(6) “Single nephron” absolute proximal reabsorption:
  (6) SNAPR=frPR .SNGFR
  where SNAPR is the single nephron absolute proximal reabsorption (nL/min), frPR is the fractional proximal reabsorption (no unit).
(7) Capillary volumic flow:
  (7) $$\mathrm{CVF}=\frac{\mathrm{SNEABF}}{\mathrm{fCapBr}}+0.5\;.\frac{\mathrm{SNAPR}}{\mathrm{fCapBr}}$$
  where CVF, capillary volumic flow (nL/min), fCapBr, capillary branching factor (no unit). The capillary branching factor (fCapBr = 6) corresponds to the average number of capillaries derived from the efferent arteriole (calculated from [44]).
(8) Tubular volumic flow:
  (8) TVF=SNGFR−0.5 .SNAPR
  where TVF is the tubular volumic flow (nL/min).

#### 2.5.2. Equations for Epithelial Transport (Na^+^, water)

(1) Single nephron Na^+^ filtered load:
  (9) $$\mathrm{SNFLNa}=10^{6}\;.\mathrm{SNGFR}\;.\mathrm{Nap}$$
  where SNFLNa is the single nephron Na^+^ load (amol/min; 10^6^ pmol to amol), Nap, plasma sodium concentration (mmol/L).
(2) Na^+^ transport (reabsorption):
  (10) $$\mathrm{TNa}=\mathrm{frPR}\;.\mathrm{SNFLNa}\;.\;\frac{1}{12}\;.\;\frac{10}{\mathrm{TubLength}}$$
  TNa (amol/min) is the sodium transport for one epithelial patch in a tubule section (10 µm-thick and TubLength µm-long); 1/12 is the number of EPI patches per tubule section, it scales local reabsorption to one patch, for tissues with 40 µm outer diameter (orTub; = see Figure 1, right); in 50 µm orTub, the scaling factor is 1/16; frPR, fractional proximal reabsorption (no unit).

#### 2.5.3. Equations for Capillary and Tubular Inputs (PTC, LUM patches)

Capillary and tubular convective oxygen delivery (and removal) by perfusion are modeled using a volume renewal factor, and used to update oxygen content The latter is calculated as tick duration divided by the volumic residence time.

(1) Capillary flow factor:
  (11) CFF=16 . 10−2 . tickdurationVcapCVF
  where CFF is the capillary flow factor (no unit), tickduration in ms (default 1 ms), CVF is the capillary volume flow (nL/min),
  Vcap is the capillary patch (PTC) volume: µm^3^ (1/6.10^−2^ converts µm^3^ to nL and min to ms).
(2) Capillary oxygen renewal (PTC patches):
  (12) $${\mathrm{qO}}_{2}\left(t+1\right)={\mathrm{qO}}_{2}\left(t\right)-\mathrm{CFF}\;.{\mathrm{qO}}_{2}\left(t\right)+\mathrm{CFF}\;.{\mathrm{qO}}_{2}\left(\mathrm{PTC}\text{\_}\mathrm{input}\right)$$
  where qO_2_(t + 1), qO_2_(t) are the PTC patch oxygen content (amol), at tick + 1 and current tick, respectivley, qO_2_(PTC_input) is the PTC oxygen feed (amol), converted from model interface PTC PO_2_ input.
(3) Tubular flow factor (TFF):
  (13) TFF=14 .16 . 10−2 . tickdurationVlumTVF
  where TFF is the tubular flow factor (no unit), TVF is the tubular volume flow (nL/min), Vlum is the luminal patch volume (µm^3^; conversion factor as above); ¼ corresponds to 4 LUM patches per tubule section (see Figure 1) (1/9 for tissues with 50 µm tubule diameter, see tissue variants in SM2).
(4) Luminal oxygen renewal (LUM patches):
  (14) $${\mathrm{qO}}_{2}\left(t+1\right)={\mathrm{qO}}_{2}\left(t\right)-\mathrm{TFF}\;.{\mathrm{qqO}}_{2}\left(t\right)+\mathrm{TFF}\;.{\mathrm{qO}}_{2}\left(\mathrm{LUM}\text{\_}\mathrm{input}\right)$$
  where qO_2_(t + 1), qO_2_(t) are the LUM patch oxygen content (amol), at tick + 1 and current tick, respectivley, qO_2_(LUM_input) is the LUM oxygen feed (amol), converted from model interface LUM PO_2_ input.

#### 2.5.4. Equations for Hemoglobin (PTC Patches)

Equations for hemoglobin-O_2_ association/ dissociation reactions are taken from [45].

(1) Rate equations and associated equations
  (15) $$\mathrm{nHill}=\frac{2.635\;.{\left({\mathrm{HbO}}_{2}\%\right)}^{2}-274.042\;.{\mathrm{HbO}}_{2}\%}{{\left({\mathrm{HbO}}_{2}\%\right)}^{2}-104.1\;.{\mathrm{HbO}}_{2}\%-31.32}$$
  (16) $$k^{\prime }c=6.325.e^{{\left(0.011537.{\mathrm{HbO}}_{2}\%\right)}^{2.88697}}$$
  NB: after proper verifications, to reduce calculation load on NetLogo, Equation (16) was replaced in RCM by a three-parameters regression obtained from Xuru-online (http://www.xuru.org):
  $$k^{\prime }c=0.04525\;.{{\mathrm{HbO}}_{2}\%}^{2.8222}+5441.2829\;\left(R^{2}=0.953\right)$$
  (17) $$\text{JdissHb}\%=k^{\prime }c\;.{\left(\alpha .P_{50}\right)}^{\mathrm{nHill}}\;.{\mathrm{HbO}}_{2}\%$$
  (18) $$\text{JassoHb}\%=k^{\prime }c\;.{\left(\alpha .P_{O2}\right)}^{\mathrm{nHill}}\;.{\mathrm{HbO}}_{2}\%$$
  (19) $${\mathrm{HbO}}_{2}\%\left(t+1\right)={\mathrm{HbO}}_{2}\%\left(t\right)-\text{JdissHb}\%+\text{JassoHb}\%$$
  (20) JdissHb=0.01 .JdissHb% .Hbt
  (21) JassoHb=0.01 .JassoHb% .Hbt
  where n_Hill_ is the Hill’s cooperativity index (no unit; [45]), HbO_2_% is the Hb % saturation, k′c is the “pseudo-velocity parameter” (mM.ms^−1^, see [45]), α is the oxygen solubility coefficient in plasma: µM/mmHg, P_50_ is the half-saturation oxygen pressure of hemoglobin (mmHg), JdissHb% is the Hb-O_2_ dissociation rate (%saturation/ms, [45]), JassHb% is the Hb-O_2_ association rate (%saturation/ms, [45]), JdissHb is the Hb-O_2_ dissociation rate (amol/ms), JassHb is the Hb-O_2_ dissociation rate (amol/ms), Hbt is the monomeric hemoglobin equivalent (amol/patch).
(2) Oxygen balance related to Hb-O_2_ association/dissociation:
  (22) $${\mathrm{qO}}_{2}\left(t+1\right)={\mathrm{qO}}_{2}\left(t\right)+4.\mathrm{JdissHb}-4.\mathrm{JassoHb}$$
  with qO_2_(t + 1) and qO_2_(t), PTC patch oxygen content (amol).

#### 2.5.5. Equations for Diffusion (All Patch Types)

We devised a diffusion procedure based on Fick’s first law, in which the outgoing amount of oxygen from one given “central” patch “i” toward each one of its four “j” Von Neumann neighbors (north, east, south and west), during one tick, is driven by oxygen concentration, the 10 µm-distance between two adjacent patch centers, the exchange surface area, and oxygen diffusivity, according to:

(23) $$\mathrm{Jdiff}\left(i\to j\right)=\frac{1}{L}.\mathrm{Dij}\;.\mathrm{Sij}\;.\left[0_{2}\right]i$$

(24) $$q_{i}O_{2}\left(t+1\right)=q_{i}O_{2}\left(t\right)+\sum_{j=n,e,s,w}\mathrm{Jdiff}\left(j\to i\right)-\sum_{j=n,e,s,w}\mathrm{Jdiff}\left(i\to j\right)$$

where Jdiff(j→i) (resp. (i→j)) is the unidirectional diffusion rate from patch j to patch i (resp. patch i to patch j; amol/ms), L is the patch-to-patch diffusion distance (µm), D_ij_ is the average diffusion coefficient of source and target patches (µm^2^/ms, or cm^2^/s), S_ij_ is the effective surface area contact between source and target patches (µm^2^), [O_2_]_i_ is the oxygen concentration of the source patch (amol/patch, or µM), q^i^O_2_(t + 1), q^i^O_2_(t) are the oxygen content of patch « i » (amol); n, e, s, w refer to north, east, south and west neighboring patches, respectively.

#### 2.5.6. Equations for Oxygen Consumption

Capillaries (PTC) and interstitium (INT): capillary specific consumption rate is extracted from literature: 1.2 ± 1.1 mM/min (SM1-Table S5). PTC patches are considered consuming through their endothelial component (0.5–1 µm thick, not represented), scaled to the corresponding crown volume fraction.

(25) $${\mathrm{JHK}}_{\mathrm{cap}}=\left[0_{2}\right]\;.\;\frac{{\mathrm{Jmax}}_{\mathrm{cap}}}{\mathrm{Km}+\left[0_{2}\right]}$$

(26) $${\mathrm{qO}}_{2}\left(t+1\right)={\mathrm{qO}}_{2}\left(t\right)-{\mathrm{JHK}}_{\mathrm{cap}}$$

with JHK_cap_ is the PTC house-keeping oxygen consumption: amol/patch/ms (patch unit, PU), [O_2_] is the patch oxygen concentration (amol/patch, or µM), Jmax cap is the PTC maximal rate of oxygen consumption (PU; see SM1-Table S6), Km is the cytochrome C oxidase Km for O_2_ (µM), qO_2_(t + 1), qO_2_(t) are the patch oxygen content (amol).

RCM also considers oxygen consumption by interstitial cells (e.g., fibroblasts, resident macrophages). This is detailed in supplement SM2 (Section 2). Consumption equations are the same as for PTC. The Km(O_2_) value of house-keeping, non-transport-related consumption is set by default to the same value as Oxphos (1.1 µM; see below).

Epithelial patches: the Km(O_2_) value of the oxidative phosphorylation is Km, Cox° = 1.1 ± 1.0 µM (SM1-Table S5).

(27) $${\mathrm{JHK}}_{\mathrm{epi}}=\left[0_{2}\right]\;.\;\frac{{\mathrm{Jmax}}_{\mathrm{epi}}}{\mathrm{Km}+\left[0_{2}\right]}$$

(28) JTNa=0.33P02[02] . TNa .frTC .[02]Km+[02]

(29) $${\mathrm{qO}}_{2}\left(t+1\right)={\mathrm{qO}}_{2}\left(t\right)-{\mathrm{JHK}}_{\mathrm{epi}}-\mathrm{JTNa}$$

with JHK_epi_ is the EPI house-keeping oxygen consumption(PU, amol/patch/ms), Jmax_epi_ is the EPI maximal rate of oxygen consumption (PU), JTNa is the transport-related oxygen consumption (PU), P/O_2_ is the ATP/O_2_ Oxphos stoichiometry (2 × “P/O”, see SM1-Table S5), 0.33 corresponds to the ATP/Na^+^ stoichiometry of the NaK-pump, TNa is the EPI sodium transport (PU), frTC is the fractional transcellular Na^+^ transport.

### 2.6. Modeling and Simulation

The cortical tissue model is transversally perfused by blood (via capillary sections) and by post-glomerular filtrate (via tubule sections). Volumic flows (and TNa fluxes) are considered occurring, on the average, at mid-length of capillaries and tubules (see Equations (7) and (8)). In the present version of RCM, proximal convoluted tubule (PCT, roughly corresponding to S1 and S2) and pars recta (PR, ~S3) are not discriminated (parameters values taken from PCT references; see SM1-Table S3a,b); similarly, distal tubule sections are not represented (see Discussion). In NetLogo, one execution step is called a tick, during which equations and rules are executed, for all patch agents, and variables accordingly updated. Tick duration used in this study was 1 ms (see Results). Note however that control simulations (not shown) indicate that the current version of RCM is able to numerically tolerates durations up to 10-20 ms, depending on flow-related inputs and parameters (RBF and filtration fraction). 

### 2.7. Data Handling and Statistical Calculations

Bibliographical and reference data are given as mean ± SD of n determinations, from N sources; when missing, the number of determinations was conservatively set to 3 (Tables in SM1, Section 2).

In a mathematical model, inputs and independent parameters (IP) are formally equivalent. However, we performed parametric and I/O sensitivity analysis (SA) separately. Local SA (i.e., one parameter varied at a time) was performed, at steady-state for tissue PO_2_ (sstPO_2_), by varying the value of the tested IP around its BV°, within its range, ±Δp (usually, 20% of BV°). The relative sensitivity coefficient, RSC, for parameter IP influence upon sstPO_2_, is calculated as:

$${\mathrm{RSC}}_{\mathrm{sstPO}2}^{\mathrm{IP}}=\frac{\frac{\left({\mathrm{sstPO}}_{2}^{p+\Delta p}-{\mathrm{sstPO}}_{2}^{p+\Delta p}\right)}{{\mathrm{sstPO}}_{2}^{p}}}{\frac{\left({\mathrm{IP}}^{p+\Delta p}-{\mathrm{IP}}^{p}\right)}{{\mathrm{IP}}^{p}}}$$

where sstPO_2_ refers to average, steady-state tissue PO_2_, p refers to reference value of the parameter being evaluated, Δp refers to the variation applied to the parameter, and IP, refers to the (independent) parameter tested.

By construction, the two main morphometric parameters, Nc/Nt (capillary density) and tubule diameter, are cast within the tissue geometry. To perform SA with these parameters, appropriate model tissues were used (see tissue variants in SM2, Section 1).

## 3. Results

### 3.1. Bibliographical Analysis and Reference Values

Biological values (BV) extracted from the bibliographical analysis categorize as: independent parameters (~33), dependent parameters (~64), three inputs (RBF, PTC and LUM PO_2_ feeds) and one main output, steady-state tissue oxygen pressure. Table 1 lists the reference values for principal inputs and parameters (BV°). The whole corpus, its analysis and the extracted values for parameters and variables are presented in the Supplementary Materials (SM1-Tables S1 to S8).

RCM° parametric setting corresponds to the model with its parameters (and inputs) all set at their reference value (BV°); nb: an improved variation of this setting, noted RCM*, will be introduced later; see Section 3.4.

### 3.2. Model Verification

Internal consistency and code checking were regularly carried out. In addition, we performed careful verification of morphological and functional aspects of RCM. For concision, we present below the verification of tissue perfusion and transport-related (epithelial) consumption processes. Details of other oxygen–related processes, i.e., the hemoglobin/oxygen equations, the patch-to-patch oxygen diffusion routine, and the non-epithelial consumption are given in Supplementary Materials (SM2). Similarly, comparative verification of the model tissue morphometry with regard to histological data from the rat renal cortex is also reported in SM2.

#### 3.2.1. Verification of Tissue Perfusion

Model perfusion, depends on a cascade of algebraic equations, from RBF down to capillary and tubular volumic flows (CVF and TVF, resp.; Equations (1)–(8)). We verified that the reference values (BV°) of the independent (or external) parameters (e.g., filtration fraction) which drive the flows yield expected values (BV°) of the flow-related dependent parameters (e.g., TVF).

**Figure 2:**
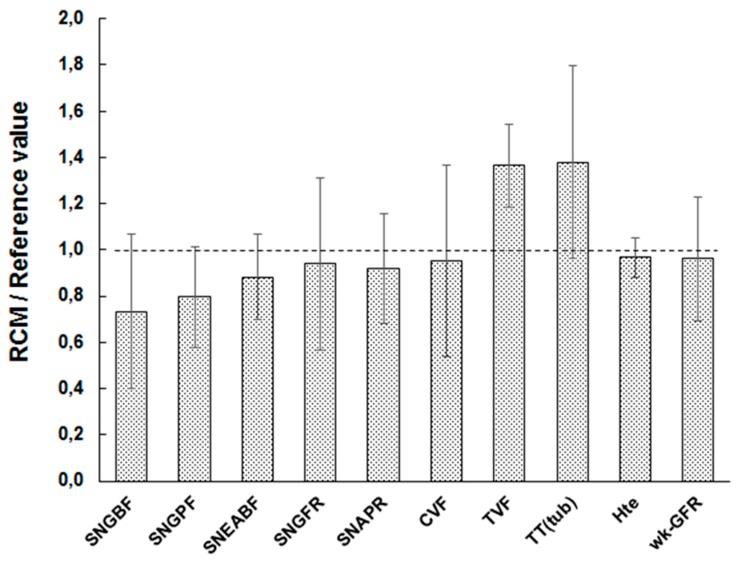
Verification of flow-related processes in the model. RCM is set under RCM° settings (reference tissue “1540”), for inputs and for independent parameters, including flow-driving parameters (nephron number, hematocrit, filtration fraction, capillary branching factor, and tissue “1540” geometry—capillary number and tubule diameter). The nine left bars correspond to the ratio of model tissue over reference value for dependent parameters. All volumic flow-related values fall within 1 SD of their bibliographical, reference value (BV°). The right-most column shows that whole kidney-equivalent glomerular filtration rate (GFR) is also accurately reproduced.

Figure 2 plots the ratio of flow-driving parameters to their BV° counterpart (all independent parameters set to BV°). Experimental CVF° is rare, and scattered in the 10–30 nL/min range [11]. For simplicity, we used 20 nL/min, the mean value, of the reported BV° (SM1-Table S2). RCM° flow-related values fall within 1 SD of their BV°, except for tubular flow TVF, slightly overestimated. Including the retro-calculation of the whole-kidney glomerular filtration rate (GFR) (wk-GFR, right-most bar in Figure 2), the mean RCM°-simulated/BV° ratio for flow-related processes is 0.99 ± 0.22 (*n* = 10, range 0.73–1.38). This argues for a fair representation and distribution of flows, and accordingly, oxygen delivery in the model.

#### 3.2.2. Verification of Transport-Related Oxygen Consumption

In EPI patches, the main oxygen consumers, the transport-related QO_2_ is dynamically calculated by converting reabsorptive sodium transport (TNa, a linear function of filtered load and fractional reabsorption, Equation (10) into oxygen cost, using the Na^+^/K^+^-pump Na/ATP and ATP/O_2_ Oxphos stoichiometric ratio (SM1-Table S4). On the other hand, the non-respiratory, house-keeping consumption (HK-QO_2_-EPI) had to be estimated from a detailed partitioning of kidney consumption. This lengthy accounting exercise, based on renal basal consumption [15], fractional cortical volume and several other parameters, is presented in Supplementary Materials (SM2). Calculations yield a reference HK-QO_2_-EPI of 1.9 ± 0.8 mM-EPI/min. Ignoring non-epithelial QO_2_ (<5% of total consumption), RCM° exhibits an oxygen consumption of about to ~7.7 mM-ctx/min, i.e., 1.18 fold the expected renal cortical consumption (6.5 ± 1.3 mM-ctx/min, SM2-Table S3). This suggests a proper assignment of oxygen consumption in the model.

A second, independent, line of verification is provided by the Na^+^/K^+^-pump activity—the ultimate driver of trans-epithelial Na^+^ reabsorption. In proximal cells, specific maximal pump activity is 396 ± 320 mM-EPI/min (SM1-Table S4). Using the pump and Oxphos stoichiometry (3 Na^+^/ATP [42], and 4.6 ATP/O_2_, resp.; see SM1-Table S4), this converts into a QO_2_ equivalent of 26.4 mM-EPI/min; on the other hand, RCM° simulated TNa-QO_2_ amounts to 10.2 mM-EPI/min, well below the above maximum. Since no explicit pump is present in RCM, these calculations indicate that TNa-related QO_2_ realistically complies with rat proximal transport capacity.

Based on the above arguments, we considered RCM to be satisfactorily verified and consistent. Nevertheless, at this point, we must point at two perfusion and oxygen-related features which remain unsatisfactorily simulated. First, the transport efficiency ratio (TNa/QO_2_) simulated by RCM° was ~11.3, below the reported range (15–26, mean value = 19; [15]). Second, RCM° apparent capillary “blood velocity”, was ~5 mm/s, somewhat outside the range of BV° (0.5–4.1 mm/s; mean 1.1 mm/s, SM1-Table S1). Addressed below (Section 3.4), both issues relate to more complex cell and tissue features, that absent from the presented model, namely energetic metabolism and capillary hemodynamics.

### 3.3. Parametric Sensitivity Analysis and I/O Analysis

In order to characterize model dependence versus parameters and input/output values, we performed parametric sensitivity analysis (SA), input/output analysis (I/O), and input-parameter interaction analysis. Detailed parametric sensitivity analysis is given in SM1, together with input-parameter interactions. Here below, we consider parametric influence upon model output (derived from SA), and the I/O analysis. With respect to model output (tPO_2_, tissue PO_2_), parametric SA allows to compare the relative influence of all parameters with each other, independently of their own numerical scale (see SM1). Second, it allows us to evaluate the influence of each parameter upon model output, given their own experimental uncertainty (SM1-Tables S1–S8). Table 2 reports the 12 most influent parameters (absolute value of associated error >0.5 mmHg); signs indicate the direction of parameter influence. For instance, experimental SD uncertainty about the transcellular Na^+^ reabsorption fraction (0.81 ± 0.20, range 0.53–1.00; SM1-Table S4, energetics and transport) translates into a tissue PO_2_ error of 2.3 mmHg.

#### 3.3.1. Parametric Sensitivity Analysis

Averaging the absolute value of errors for all 22 parameters, we obtain an estimation for (parameter-dependent) model accuracy of 1.1 ± 1.1 mmHg (range 0.0–3.1). Since experimental SD has no sign by definition, we conservatively consider RCM error as twice this value, 2.2 mmHg. Within RCM, the standard-deviation of PO_2_ patches is 4–5 mmHg.

#### 3.3.2. Input/Output Analysis

**Figure 3:**
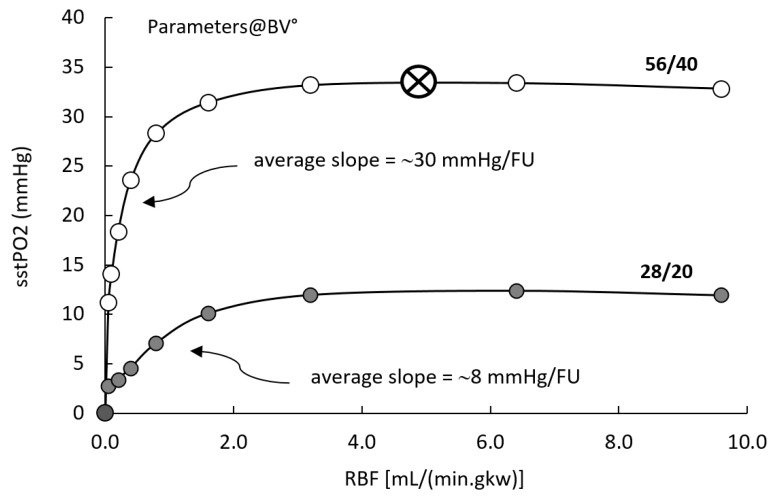
Input/output analysis. Tissue PO_2_ (output) as a function of RBF, and PTC and LUM PO2 (inputs). RCM° parametric configuration. RBF is varied from zero to 10 mL/(min.gkw) (flow unit, FU), while capillary and luminal PO_2_ inputs are set either to reference conditions (PTC/LUM = 56/40, open circles)or to reduced oxygen feed (PTC/LUM = 28/20, grey circles). The open circle/cross symbol indicates the RBF and PO_2_ feed reference conditions.

RCM features three main inputs, RBF and feeding PO_2_ via LUM and/or PTC patches. The following analysis was carried with all parameters set at BV°. Figure 3 presents model response in term of tissue PO2 (sstPO_2_), as a function of RBF, at two combinations of feeding PO_2_ from PTC (capillaries) and LUM (tubules). In the reference condition (open circles, PTC/LUM = 56/40), the sstPO_2_ is practically insensitive to RBF (slope ~0.0 mmHg/FU) in the 3.0–10.0 mL/(min.gkw) (or “flow units”, FU), around BV° (5.3 FU). Conversely, when RBF decreases below 3 FU, its control over model PO_2_ progressively increases: around 2.0 FU, the control slope increases to 2.5 mmHg/FU, while it sharply increases below 1.0 FU, to ~15–50 mmHg/FU. Using lower PO_2_ inputs (PTC/LUM = 28/20, grey circles), the model PO_2_ response to RBF is qualitatively similar, exhibiting insensitivity to RBF in the 3–10 FU range and marked increase below 2.0 FU; however, in the latter ischemic range, RBF control over sstPO_2_ is more modest, in the 5–8 mmHg/FU range.

**Figure 4:**
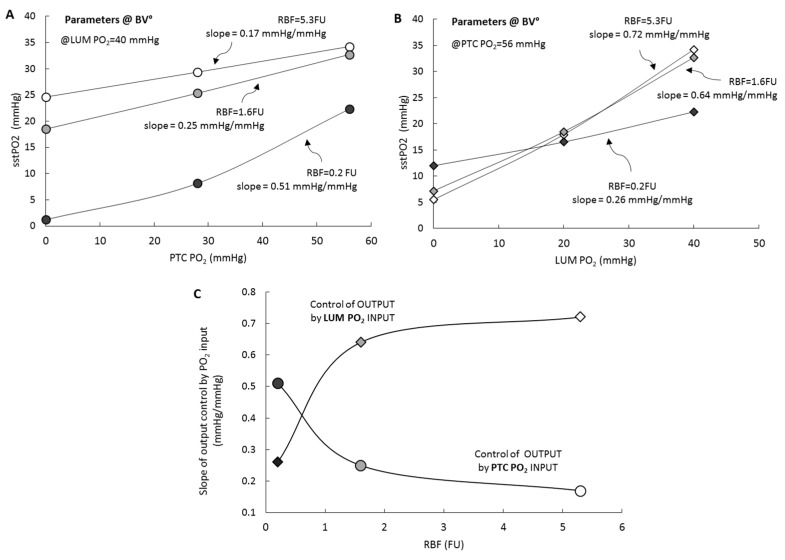
Input/output analysis. Tissue PO2 (output) is plotted as a function of PTC and LUM PO2 (inputs), at three levels of RBF (5.3, 1.6 and 0.2 FU, respectively white, grey and black symbols). RCM° parametric configuration. (**A**) (circles), PTC input is RBF is varied, while luminal PO2 input is kept constant (at its reference value, 40 mmHg). (**B**) (diamonds), LUM input is RBF is varied, while PTC PO2 input is kept constant (at its reference value, 56 mmHg). (**C**) Summarizes the results by plotting the output/input slopes for PTC (circles) and LUM (diamonds); symbols are color-coded as in A and B.

We then considered model output as a function of PO_2_ inputs. Figure 4 (upper panel) shows model output (sstPO_2_) response as a function of PTC PO_2_ input, panel A (resp. LUM, panel B), at three levels of RBF perfusion, while LUM PO_2_ input (resp. PTC) is kept fixed at its reference value. For each level of RBF, we determine the slope of the output dependence versus the input (nb: calculation performed around the reference value, 56 and 40 mmHg for PTC and LUM, resp.). Panel C plots the output/input slope dependence as a function of RBF. It can be seen that under reference perfusion (RBF°) the influence of PTC input on RCM PO_2_ is 0.17 mmHg/mmHg while for LUM it is 0.72 mmHg/mmHg; when RBF is reduced, the control exerted by PTC and LUM inputs respectively increases and decreases and the relative influences of PO_2_ inputs is inverted.

#### 3.3.3. Interactions of Inputs with Independent Parameters

Because of the number of parameters, this analysis is given and detailed in Supplementary Materials (SM1). In summary, parameter control over model PO_2_ is not overall modified with oxygen inputs, (see SM1 and SM1-Figure S2). Thus, we also infer from this analysis that model “error” will not change significantly when modifying its oxygen feeding or perfusion inputs.

#### 3.3.4. Patch-Type Based Analysis

**Figure 5:**
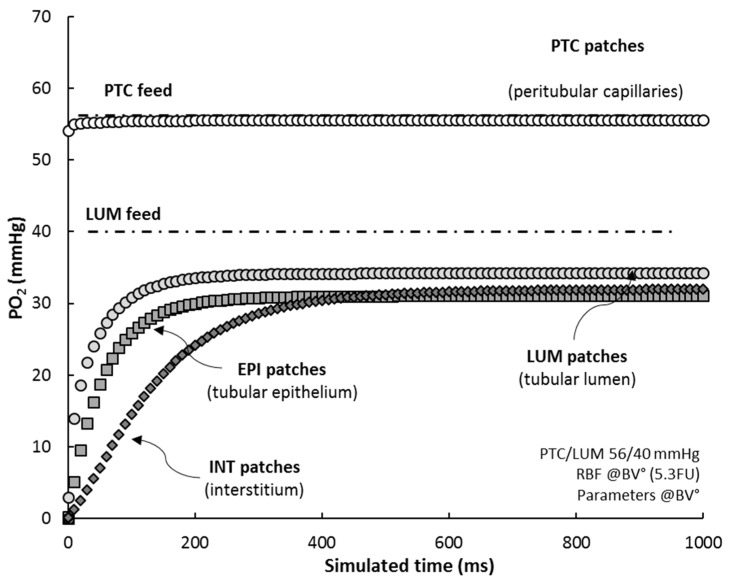
RCM “histological” compartments. Average PO_2_ for each patch-type compartment is shown. All patches were initialized at zero PO_2_ and RCM was simulated until steady-state (RCM°, RBF = 5.3 FU and PTC/LUM feed = 56/40 mmHg; sampling time 10 ms). For comparison, PTC and LUM feeds are also plotted (point-dotted lines).

Based on the patch-mapping of the renal cortex in RCM tissue, the model allows to monitor the oxygen level of its “histological” individual components. When simulating RCM under reference conditions (RCM°), from initial state (all patches set to zero) up to steady-state, we observed that (Figure 5):

(i) the average PO_2_ of PTC’s (white circles) adjusts almost exactly to PTC feed (Hb saturation at 75%), within 0.5–1.0 mmHg, in ~5 ms;
(ii) PO_2_ in LUM patches (grey circles) settles to 35 mmHg, some 5 mmHg lower than their PO_2_ feed, in about 350 ms (half-time 20 ms), indicating that LUM patches deliver oxygen to the tissue;
(iii) EPI patches (grey squares) reach a steady-state level, 31 mmHg, in ~250 ms (half-time 35 ms);
(iv) INT patches (dark diamonds), characterized by a low consumption, progressively fill up with oxygen, up to ~32 mmHg, with a delay of ~100 ms with regard to EPI patches (half-time 110 ms); after 500 ms, the interstitial compartment stabilizes at 32 mmHg, ~1 mmHg higher than EPI patches.

#### 3.3.5. Influence of Hemoglobin

In the parametric sensitivity analysis (see above), we observed, that, under normal conditions, hemoglobin-related parameters exhibit limited control over tissue PO_2_ (P_50_ and “blood” Hb concentration; see SM1 for details). In RCM, hemoglobin function is coded so as to allow the de-activation of its equations. Under normal conditions (RCM°), when the model is run in the absence of hemoglobin, sstPO_2_ output is reduced by ~0.1–0.2 mmHg, a surprisingly low value, well within model error.

We further analyzed this observation by evaluating the influence of Hb on tissue PO_2_, as a function of the RBF input and PO_2_ inputs.

**Figure 6:**
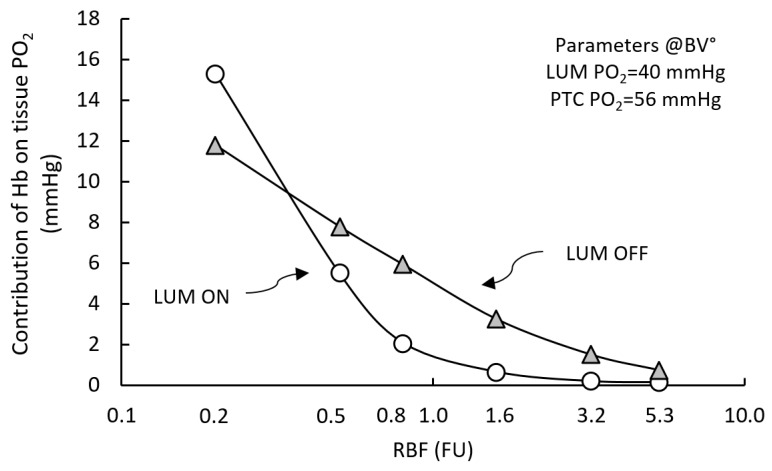
Contribution of hemoglobin on tissue PO_2_ as a function of RBF input. RCM° parametric configuration. Hemoglobin (Hb) contribution to tissue sstPO_2_ (ΔHb) is plotted as a function of RBF (open circles). ΔHb is negligible close to normal RBF (<1 mmHg, within model error), but it increases markedly with decreasing RBF, up to ~15 mmHg at 0.2 FU. Deactivating luminal convection shifts the curve rightwards, “unmasking” a substantial control of Hb over tissue PO_2_ (~1–10 mmHg, grey triangles).

Figure 6 shows that the influence of Hb on sstPO_2_ increases when it is decreased, reaching 6 and 15 mmHg at RBF 0.5 and 0.2 FU, respectively (open circles).

Further exploring this effect, we observed that when LUM convection is de-activated (grey triangles), Hb contribution to tissue PO_2_ it is potentiated between in the 0.5 to 5.0 FU RBF range (1–4 mmHg)). Overall, Hb control of sstPO_2_ is in the relatively low, e.g., 12 mmHg under severely decreased 0.2 FU). The influence of both PO_2_ inputs on Hb contribution to tissue PO_2_ was also evaluated. We observed the low RBF-dependent control of Hb contribution depends mostly on PTC feed (ΔHb ~10 mmHg), versus LUM feed (ΔHb 2–3 mmHg; data not shown). Thus, when significant (under low RBF input), Hb control depends primarily on PTC feed. Accordingly, it vanishes when Hb is de-activated. Finally, under transient conditions, Hb alters noticeably RCM dynamic response. Setting RBF to zero from pre-established RCM° tissue steady-state, if Hb is deactivated, the ischemic relaxation time to complete tissue anoxia (tPO_2_ < 1 mmHg) decreases two-fold by comparison to RCM with active Hb (from ~600 to 300 ms). Hypoxemic relaxation time is ~210 ms with Hb, vs. ~160 ms without Hb.

### 3.4. Physiological Adjustments and Fine-tuning

#### 3.4.1. Tubule and Reabsorption-Related Adjustments

When independent parameters and inputs are set to their reference values (RCM°), model PO_2_ (sstPO_2_) is 33.5 ± 2.2 mmHg, within the experimental, reference determinations of tissue PO_2_ in the outer (ocPO_2_°) and inner (icPO_2_°) cortex, 40.8 ± 6.2 mmHg and 29.8 ± 1.3 mmHg, respectively (see SM1-Table S1). Using 0.66 and 0.33 fractional volumes for the “outer” (superficial and mid-cortical nephrons), and inner (juxta-medullary nephrons), respectively, yields a cortical weighted average of 36.9 ± 4.6 mmHg. Since BV° relates to an “average” cortex, this represents preliminary evidence for RCM validity. There are, however, issues that need to be considered for model validation, all of them relating to Na^+^ reabsorption and oxygen consumption. First, our bibliographical analysis is by definition based upon experimental determinations—with their own limitations, and some parameters require appropriate tuning. Second, the proximal tubule (PT), and especially its convoluted part (PCT, 2/3rd of PT), has the ability to drive Na^+^ reabsorption via a basolateral Na^+^-HCO_3_^−^ cotransport, independently of the Na^+^-K^+^-pump, and without costing ATP or oxygen [22,46].

The BV° for fractional reabsorption is based on experimentally accessible parts of the proximal tubule (PT), neglects the distal tubule (DT, 2.0 mm, 9% reabsorption), and ignores reabsorption by the medulla (25%; see SM2). Accordingly, we correct RCM° settings by: (i) adjusting effective tubule length from 10.4 to 12.4 mm and fractional reabsorption from 0.51 to 0.75. The BV° value of transcellular reabsorption fraction (frCTNa° = 0.81) translates into ~18.0 TNa/QO_2_ ratio, whereas the experimental one, for the cortex (27.0, SM1-Table S4) converts into a 0.41–0.63 transcellular fraction (mean 0.56; [30,31,47]; SM1-Table S4). Adjusting frCTNa from 0.81 to 0.56, the bicarbonate factor (fBic) from 1.15 to 1.5, and increasing the ATP/O_2_ ratio from 4.5 to 5.5, yielded tissue PO_2_ to 35.9 mmHg (TNa/QO_2_ ~19). Table 3 summarizes the adjusted RCM setting (RCM*), with respect to reference setting, RCM°.

#### 3.4.2. Capillary Flow and Hemodynamics

An important issue requires attention, relating to capillary microcirculation. In RCM, capillary flow is a simple distribution of post-glomerular flow down to capillaries, according to the capillary branching factor (Equation (7); [44]). This yields an apparent capillary “blood fluid” velocity of 5 mm/s (at RBF° 5.3 FU), an obvious overestimation *versus* the experimentally reported value (BV° 1.1 ± 1.3 mm/s; SM1-Table S2). The corresponding capillary residence time of oxygen (~2 ms) might have been limiting its diffusion toward the nearby epithelial patches (EPI)—a possible explanation for the weak control exerted by hemoglobin on tissue PO_2_. We tested this hypothesis by applying a 0.25 “capillary factor” to PTC flow, thus increasing oxygen residence time 4-fold, to 8 ms. The apparent capillary velocity was reduced to 1.2 mm/s (within BV° range): this increased Hb control over tissue PO_2_ t0.7 mmHg (vs. 0.1 mmHg). Setting the capillary test-factor to 0.1 (velocity 0.5 mm/s, ~20 ms residence time), further increased hemoglobin contribution to tissue PO_2_ (ΔHb) to 1.5 mmHg, still lower than model accuracy. This issue will be thoroughly addressed in the Discussion, by comparison to well-studied, non-renal tissues. Because of the multi-parametric/input combinatorial, no further attempt was made to “optimize” RCM. A new model setting was defined and referred to as RCM* (see Table 3).

### 3.5. Model Validation

#### 3.5.1. Steady-State Condition

**Figure 7:**
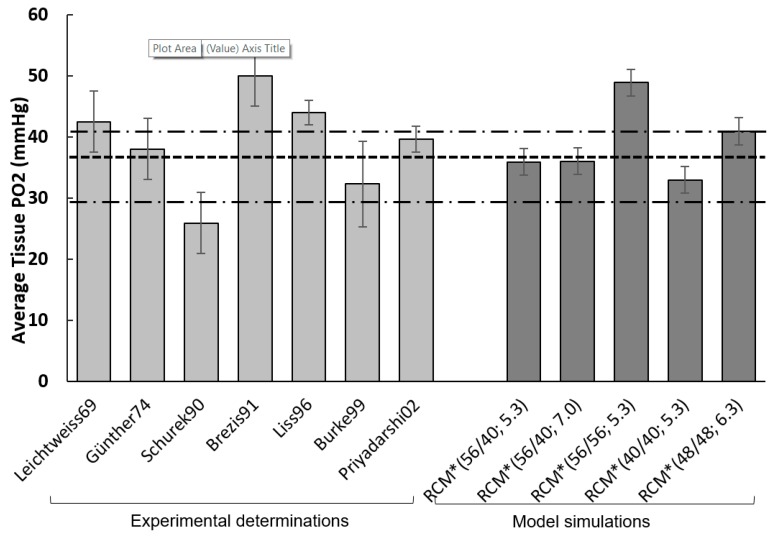
Validation under steady-state conditions. RCM simulations (dark grey) are compared to experimental references (light grey), all based on tissue PO_2_ measurements in rat renal cortical tissue using micro-electrodes (thus providing an average measure of tissue PO_2_; please recall that reported experimental values for the cortex range ~20 to 75 mmHg). Thin point-dotted lines correspond to the BV° for the outer cortex and inner cortex; the thick line (36.9 mmHg) represents the weighted cortical average (0.66 *v*/*v* outer cortex, 0.33 *v*/*v* inner cortex). RCM simulations were run under RCM* adjusted parametric setting (see text), with a panel of selected inputs combinations. Except for the 56/56 feed, RCM PO_2_ falls within the SD range (see SM1-Table S1). Leichtweiss69 [11]; Schurek90 [48]; Burke99 [20]; Brezis91, cited in [13]; Günther74 PMID: 4603896; Liss96 PMID: 8731090; Priyadarshi02 PMID: 11849394.

Figure 7 compares five RCM* model output sets (corresponding to various input combinations, all within experimental scatter) to seven experimental references, all based on direct tissue PO_2_ measurements in rat renal cortical tissue using micro-electrodes. The thin point-dotted lines correspond to the BV° for the outer cortex and inner cortex (40.8 and 29.8 mmHg; see SM1-Table S1); the thick dotted line (36.9 mmHg) represents the cortical average (see above).

RCM*(56/40; 5.3) produces a tissue PO_2_ of 35.9 mmHg. RCM* (56/40; 7.0), with sstPO_2_ = 36.0 mmHg, corresponds to a slightly increased RBF input (within half the reference scatter, SM1-Table S1). RCM* (56/56; 5.3FU), with a tPO_2_ of 48.9 mmHg reflects the influence of LUM PO_2_ feed. The two other simulations correspond to oxygen feed set at the same value, low (40 mmHg) or high (48 mmHg), again within half the experimental scatter. With the exception of the 56/56 mmHg feed, RCM PO_2_ falls within the experimental range, and, interestingly, closer to the outer cortex value.

#### 3.5.2. Transient Conditions

Beyond steady-state, we explored RCM behavior under transient conditions: when RBF input is set to zero (simulating ischemia) and when PTC and LUM oxygen feed inputs are set to zero (simulating anoxemia). For comparison with available experimental data (see SM1 Table S1), two variables are monitored vascular oxygen (as PTC PO_2_) and average tissue oxygen; in addition, we monitor Hb saturation level).

**Figure 8:**
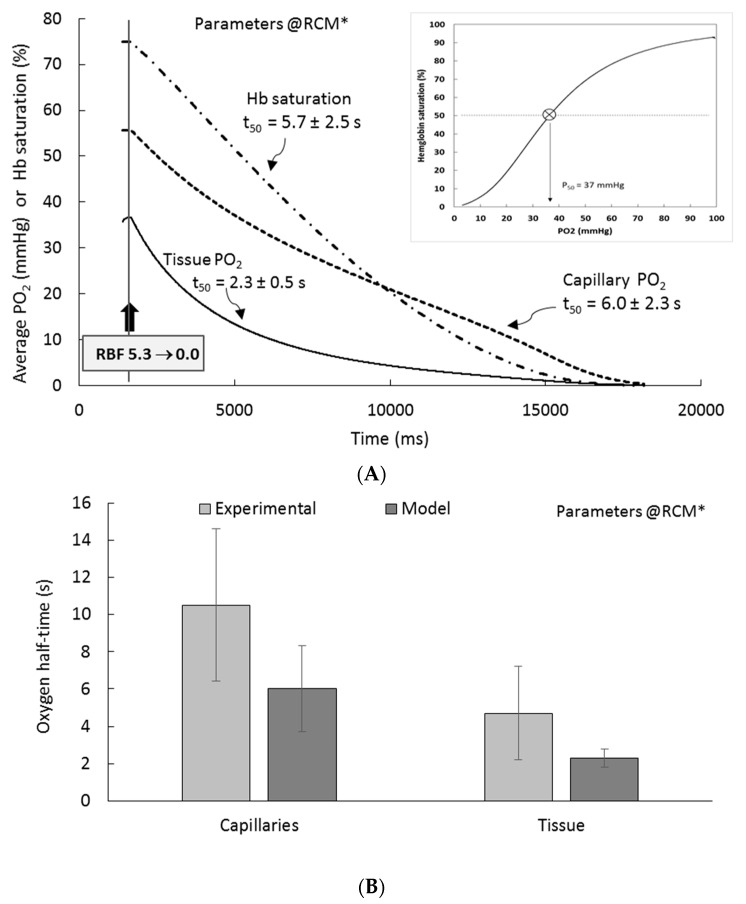
Ischemic half-time of oxygen in RCM. (**A**) At steady-state, RBF is set to zero, simulating “ischemia”: capillary PO_2_, Hb saturation, and tissue PO2 are monitored Average tissue PO_2_ (black line) decreases progressively toward zero (PO_2_ < 0.5 mmHg), with a “half-time” (t_50_) of 2.3 ± 0.5 s. Capillary PO_2_ (“PTC PO_2_”, dotted line) and hemoglobin saturation (dot-point line) are also shown. Inset of panel A, Oxygen dissociation curve of hemoglobin, reconstructed from RCM simulation (50% saturation, 37 mmHg). (**B**) Comparison of oxygen half-times for capillaries and for tissue to experimental values.

Figure 8 (panel A) shows model (RCM* setting) “ischemic” time-course for capillary oxygen (PTC PO_2_), Hb saturation (Hb % saturation), and tissue PO_2_ (tPO_2_), i.e., after RBF has been set to zero; the inset shows the saturation curve of Hb as a function of PO_2_. From PO_2_ decrease, we define ischemic (respectively anoxemic) oxygen “half-time” (t_50_), i.e., the time required for the variable to reach half of its initial value after RBF (resp. PO_2_ feed) has been set to zero. Panel B of Figure 8 summarizes RCM t_50_ values (dark grey bars), and compares them to experimental references (light grey bars, see BV° in SM1-Table S1). For capillary oxygen, ischemic oxygen half-life is 6.0 ± 2.3 s, as compared to the reference “micro-vessels” value (10.5 ± 4.1 s, range 6–30), slightly faster than experimental values, but within SD (see panel B). NB: t_50_ error estimated as model accuracy (2.2 mmHg) divided by the local slope of PO_2_ decrease. For the tissue, ischemic PO_2_ half-life is 2.3 ± 0.5 s, to be compared to the reference value 4.7 ± 2.5 s (see panel B), although somewhat faster, the model reproduces the experimental cortical oxygen ischemic half-life within experimental SD. As expected, the t_50_ of Hb saturation was similar to capillary t_50_ (5.7 vs. 6.0 s; point-dotted line in panel A). Of interest, in view of the limited contribution of hemoglobin to model PO_2_, the ischemic response of RCM was also simulated in the absence of hemoglobin: without Hb, tissue oxygen decrease is accelerated ~5-fold, to from 2.3 to 0.45 s (no experimental value available). In PTC patches alone, in the absence of Hb, ischemic oxygen decrease is accelerated 10-fold to 0.25 s (vs. 6.0 s when Hb is active). This indicates and confirms that Hb is well able to release oxygen, depending on conditions.

Finally, we tested RCM under anoxemic dynamic conditions (not shown). When feeding PO_2_ inputs are set at 0/0 mmHg from steady-state, oxygen drop is very fast, both in CTX (t_50_ ~30–40 ms) and in PTC (t_50_ = 5–10 ms). These values decrease to ~20 ms and ~4 ms, respectively, when Hb is de-activated.

## 4. Discussion

### 4.1. Generalities

The current understanding of physiological regulation of kidney oxygenation remains uncomplete, if not “rudimentary” [49]. So is the exact contribution of hypoxia-reoxygenation and tissue hypoxia in the initiation and progression of renal injury and disease. The lack can be appreciated by the decisive, still unanswered questions listed by expert reviewers (see [1,2,49,50]), as well as by the numerous modeling and simulation (MS) studies proposed since the late 90’s and early 2000’s. From Lubbers and Baumgartl in 1997, to Lee and et al. in 2017 [10,12], some twenty modelling and simulation (MS) studies were dedicated to renal oxygenation (see [26,27,50] for references), among which only four address the cortex [10,22,35,51]. Among the latter, none considers the cortical heterogeneity per se at the histological level, although Lee and et al.’s pseudo-3D model does integrate vessel compartments down to a 2D capillary-tubule model, able to predict the PO_2_ in the cortical tissue and micro-vessels [26].

Focusing on cortical oxygenation at the “histological” level, RCM is proposed as a complementary step to other MS approaches toward the full, yet to reach, description of renal tissue in response to ischemia-reperfusion (IR). Since oxygen deficit and oxygen reintroduction are primary events in this sequence, we developed RCM as a detailed model of oxygen distribution and consumption in the cortex. By construction and parameterization, it is an average cortical slice, representing mostly the proximal convoluted tubule (PCT, ~60% the cortex), and secondarily the pars recta (PR, ~25%) and the distal tubule (DT, ~15%), with no segmental specificity at this stage. In order to accurately cope with the dynamics of the oxygen diffusion process, hemoglobin-oxygen interactions, and trans-tissular “convective” feeding flows, the time scale ranges from the millisecond to tens of seconds. In particular, RCM specifically considers the possible oxygen delivery by the tubular luminal flow to the adjacent epithelial cells. It is an altogether different endeavor than all other MS proposed up to now, which uses sophisticated mathematical MS tools at all uphill renal levels, but the tissue itself.

### 4.2. Bibliographical Analysis for the Rat Kidney

This work is the very first detailed, dynamic and physiologically-based model of renal cortical oxygenation at the cell-tissue level. Great care was dedicated to parameterize the model in terms of one specific species thanks to an extensive bibliographical search. The Rat species was chosen, as opposed to Man, Pig and Mouse, because it remains the most studied animal model in general (~2 million rat publications in PubMed©, as of October 2019), and for renal studies in particular.

### 4.3. Sensitivity Analysis, Parameters, Inputs and Interactions

#### 4.3.1. Parameters

Sensitivity anaysis (SA) showed that the most influential parameters relate to morphometric features and reabsorptive/energetic processes. Based on SA, we calculated the “error” propagated in RCM for each parameter, given its variability. Most “sensitive” parameters (e.g., fractional transcellular fraction) exert a sizable influence on sstPO_2_ (1–3 mmHg). We conservatively estimate model error to ~2 mmHg.

#### 4.3.2. Inputs

RCM features three different inputs (RBF and PO_2_ feeds), and the sstPO_2_ output, in addition to patch-type PO_2_. Since we want to address cortex behavior under experimental situations far apart from “normal” physiology (e.g., ischemia, hypoxemia), inputs were scanned over ranges wider than references.

Except for electrode-based studies by Welch and et al. [52,53,54], intra-tubular and intra-capillary PO_2_ have not been measured, although uphill PO_2_ in glomerular capillaries has been measured in cases [48]. According to Welch and et al., tubular values are confidently measured as within the lumen (~40 mmHg), but “capillary” values actually refer to sub-capsular “star vessels” (B. Welch, personal communication). Although they are comparable to (microelectrode) measurements of superficial glomeruli PO_2_ (~46 mmHg) by Schurek and et al. [48], such values may or may not correspond to deeper, intra-cortical peritubular capillaries. Intra-microvascular (not necessarily capillary) values were determined by Ince and et al., using dual wavelength phosphorimetry [55,56], yielding somewhat higher values (see SM1-Table S1). As reference input values, we used RBF 5.3 (mL/(min.gkw, or flow units, FU), PTC PO_2_ 56 mmHg and LUM PO_2_ 40 mmHg. Relating to our working hypotheses and the luminal side of the tubules, it is noteworthy that indeed sizable levels of PO_2_ have been reported (40 mmHg), i.e., not very different from the capillary level (56 mmHg, see above). This appears at variance, if not conflict, with the conventional hypothesis that luminal contribution to tissue oxygen is to be neglected [1]. A priori, one cannot see why luminal oxygen could not feed close-by epithelial cells.

At reference input values, and over a wide range around these, PO_2_ output is highly dependent on PO_2_ feeds, and weakly controlled by RBF, down to 1.5 FU. At lower RBF, tissue PO_2_ decreases rapidly towards zero. This means that, at least at normal PO_2_ feed, oxygen delivery remains greater than consumption as long as RBF is in the “quasi-normal” range (2.0–10.0 FU). On a patch basis, low RBF induces a continuous decrease of epithelial and interstitial PO_2_ (control slope ~10 and ~1 mmHg/FU, resp.), while capillary PO_2_ remains stable down to ischemia (0.01 FU). This is due to the high hemoglobin saturation (~76% at 56 mmHg).

Our model-based observation of the low control of cortical PO_2_ by RBF (see Figure 3) is reminiscent of experiments by Evans and et al., in Rabbit, showing that cortical PO_2_ is relatively stable (−10%, or 4 mmHg) under altered cortical perfusion (−30% from baseline) [57,58]; experiments in Sheep yielded similar results [57]. In rat, Emans and et al., evidenced a cortical PO_2_ decrease (21%, or ~8 mmHg) upon systemic administration of angiotensin II and concomitant RBF reduction (−50%) [49]. From these in vivo determinations, the authors suggested that a ~30% threshold to RBF decrease applies before cortical hypoxia develops [49]. However, our model is an “ex vivo” construct in which organ and systemic regulations are missing. Nevertheless, the above suggest that the relatively weak control of RBF upon tissue PO_2_ might be a specific renal cortical feature, possibly related to the low extraction ratio. Again in vivo, cortical PO_2_ stability vs. perfusion went unmodified under increased consumption (2-fold, mitochondrial uncoupler, [57]): we simulated this situation by decreasing RBF 2-fold at a 2-fold increased QO_2_: model PO_2_ remained within 1 mmHg from control. Overall, the weak control of RBF over tissue PO_2_ (2.5 mmHg/FU) is quantitatively comparable to experiments, slightly left-shifted towards RBF levels halved versus reference (“pre-ischemic” levels”). Within model limits, RCM implementation of local oxygen “delivery to consumption” appears to properly describe cortical oxygenation.

The second category of RCM input is represented by PTC and LUM PO_2_ feeds. In RCM, under reference conditions, oxygen feeding by luminal PO_2_ dominates the control of tissue PO_2_ by a ~5-fold factor (0.72 vs. 0.17 mmHg/mmHg, for LUM and PTC PO_2_, resp.). This observation suggests that, in in vivo reference conditions, oxygen delivered by the tubules could be of significance, and possibly higher than capillary oxygen delivery (see below for a detailed analysis). Under ischemic condition, capillary control increase three-fold, becoming dominant, with 0.51 mmHg/mmHg vs. 0.26 mmHg/mmHg, for luminal side.

#### 4.3.3. Interactions between Inputs and Parameters

Exhaustive scanning of all possible parameter-parameter and input-parameter interactions was not carried out. Nevertheless, simulations indicated that the parameter and inputs influences did not modify the relative importance of inputs control over PO_2_ output (feeding PO_2_ > RBF), nor the dominant contribution of luminal oxygen vs. capillaries.

### 4.4. Verifications, Adjustments and Model Validation

#### 4.4.1. Model Verifications

For simplicity, we defined RCM as a 2D-idealized slice, plunging radially into the cortex. This choice of an idealized cortex as dictated by “histological” definition (10 µm) as well as typical dimensions of tissue preparations (1/10 mm^2^). It was as comforted by our verifications that model tissue geometry reproduces rat cortical morphometric indexes.

The reference RCM tissue exhibits morphometric features of real cortex, including the fractional volume of the histological compartments [41]. This is important because, by definition and similarly to experimental measures, the average tissue PO_2_ integrates oxygen from all compartments, on a fractional volume basis.

From a functional standpoint, we verified flow distribution and oxygen processes. For perfusion, we verified that capillary and tubular volumic flows are adequately reproduced [59]. Oxygen-related processes were verified individually. For oxygen diffusion, one dedicated routine was code (Fick’s first law). It was then verified to comply with known chemical physics), for both unidirectional and net diffusion (details are given in SM2).

The different terms of oxygen consumption were difficult to parameterize because most sources of information relating to whole kidney consumption, whereas RCM targets sub-compartments and cell types within the cortex. Moreover, renal regulations and experimental protocols generate additional experimental variability. Further, although in normal conditions transport-related cost dominates total QO_2_, non-transport (house-keeping) consumption gains increasing importance as transport load decreases, particularly under situations of interest such as ischemia. We extracted “capillary” consumption from [60,61,62]. This particular figure relates to capillaries and endothelial cells, but it is neither rat-specific (the sole exception in this work) nor kidney-specific, nor specific of renal peritubular capillaries. Then, we partitioned reference renal consumption down to house-keeping (EPI and PTC) and transport (EPI) related consumption of respiring patches. Numerous verifications were performed: (i) the final, effective consumption by RCM corresponds, within 25%, to expected renal cortical consumption, (ii) independently, transport-related QO_2_ of EPI patches (~7 mM/min) complies with rat proximal (and distal) transport capacity (~26 mM/min, under reference conditions), and (iii) as a corollary, these values indicate that the epithelial Na^+^-K^+^-pump would function at ~30% of its maximal rate, which corresponds to 4–12 mM intracellular Na^+^ [63]; indeed, this value compares well to the intra-epithelial Na^+^ concentration, in the 5–20 mM range [46].

Because simulation of RCM leads to the unexpected observation that, at least under reference conditions, hemoglobin contribution to tissue PO_2_ is limited, specific comments about hemoglobin verification, parameters, and equations, are called for. As opposed to Hb-O_2_ equilibrium, very few descriptions of Hb-O_2_ dynamics are available [45,64,65]—and none for the rat. We use Hb-O_2_ dynamic equations of Gutierrez [45] (see SM2 for details and justifications).

First, all equations from Gutierrez [45] were thoroughly verified by checking association/dissociation dynamics, their relative influence upon the equilibrium constant, and the saturation curve. Second we performed additional verifications, including (i) the substitution of Gutierrez sigmoidal saturation equation by a simpler hyperbolic equation (as in [64,65]) and (ii) the implementation of a crude, “linear” hemoglobin (saturation as a linear function of PO_2_, half-point at 37 mmHg, 100% beyond 74 mmHg; more details about Hb and equations used are given in SM2).

#### 4.4.2. Physiological Adjustments

Under RCM° settings, model sstPO_2_ amounts to ~34 mmHg, well within the experimental scatter. This constitutes one additional verification, and one step towards RCM validity. However, some parameters diverged from the normal regimen and required additional tuning.

With the RCM° configuration, fractional “proximal” reabsorption, frPR, was set at reference value (51%). However, real proximal fractional reabsorption is higher because experiments only quantify reabsorption based on the accessible proximal tubule length, leading to a significant underestimation. “Proximal” sodium reabsorption is considered to be 60–75% of filtered load (e.g., [46]). Fractional distal reabsorption amounts to some 9% (and 2 mm long). We adjusted both length and fractional reabsorption (12.4 mm and 75%, resp.), so that fractional medullary reabsorption (absent from RCM), lies in its normal range 0.2–0.3 [66].

The next tuned parameter is the fractional transcellular (or “active”) epithelial sodium reabsorption. Renal proximal tubule carries vectorial transport, reabsorbing NaCl and water near-isotonically. Driven by the ATP-consuming basolateral Na^+^-K^+^ pump, transepithelial reabsorption occurs via a panel of transcellular and paracellular pathways, involving filtered solutes (including bicarbonate), and relying on an axial distribution of processes [33,46]. These complex couplings allow the proximal tubule to reabsorb Na^+^ with higher efficiency than expected from the sole action of the basolateral Na^+^, K^+^ pump (12–18 transported Na^+^ per O_2_) [34,47,67].

For reference, we used an axially-averaged value for transcellular fraction (0.81, see SM1—Table S4). This corresponds to ~18–20 Na^+^/O_2_, a value well below the 24–30 experimental ratio for PT or the cortex [31,47], equivalent to a transcellular fraction ~0.46–0.63. Finally, we increased the ATP/O_2_ ratio (4.5 to 5.5), assuming that the cortex energetic metabolism and Oxphos are optimally fed [68,69].

#### 4.4.3. Model Validation

Tissue PO_2_ output of RCM° (BV°) and RCM* (fine-tuned) is well within reported experimental values of cortical PO_2_. This was expected because RCM and its settings are based on careful bibliography, analyses, and verifications.

Steady-state validation (tissue): RCM individualizes two oxygen inputs, capillaries, and tubules. Accordingly, most simulations were run with PTC/LUM PO_2_ set at their reference values, 56/40 mmHg). In most renal studies, oxygen is measured either: (i) with polarographic, Clark-type microelectrodes (e.g., [12,54]) or fluorescence optodes (as in [16,70]), yielding average tissue PO_2_, or (ii) vascular-confined oxygen-sensitive phosphorescent dyes, addressing vascular and micro-vascular PO_2_ [55,56]). Despite such a variety of methods, careful cross-examination of references and data with the (Rat-based) mathematical model by Gardiner and et al. [24,25], indicates that “peri-glomerular” PO_2_, including post-glomerular PTC, most probably lies in the ~40–50 mmHg range, at least in superficial cortex and nephrons (45 ± 6 mmHg in [54], 46 ± 13 mmHg in [48]). Scanning this range, yielded sstPO_2_ well corresponding to reference values, RCM* (56/40; 5.3) producing a tissue PO_2_ of 36 mmHg. Well within experimental scatter, the latter value lies closer to the outer cortex, possibly because the inner cortex represents less than 25% of the cortex and most reference values derive from experiments relating to the outer cortex and superficial nephrons.

In 2001, Welch and coll. provided the first measurements of intraluminal PO_2_, in addition to post-glomerular and interstitial PO_2_ [52,53,54], thus addressing two out of the four RCM compartments. We used their data to try and complete RCM validation on a local, patch basis (data not shown). Briefly, when set at experimental values of input (45/39 mmHg, 5.1 FU,), model sstPO_2_ was 34–35 mmHg, again within experimental scatter; note however that since average tissue PO2 is not reported in [54] no comparison is possible. On the other hand, as expected, PTC remained close to its feed value (44.7 mmHg) and LUM PO_2_ decreased to 34.9 mmHg, a value differing by ~3 mmHg from the reported one (39.0 ± 4 mmHg SD, “PT” in Table 2 of [54]). Similarly, simulated interstitial PO_2_ (37.5 mmHg, INT patches) was markedly lower (by 4–5 mmHg) than reported (42 ± 7 mmHg SD; “OC”, Table 2, [54]). Overall, RCM did not “satisfactorily” predict the “local” experimental values from [54]. In addition to RCM limitations (see below), in experiments from [54], the “interstitial” compartment is said to be “not so precisely defined”, the average PO_2_ of the tissue is not given, and values pertain only to accessible, superficial nephrons, with no definite segmental positioning. Thus, disappointing as they seem, these observations result from a combination of experimental uncertainties and model-related limitations.

Dynamic validation: In order to validate RCM under transient conditions, we determine two dynamic variables. First, the “ischemic” oxygen half-life, the time required for the average capillary and tissue PO_2_ to reach 50% of their initial value, when RBF is set to zero (thus capillary and tubule volumic flows). Second, by analogy, the “anoxemic” half-time, when oxygen feed is set to zero (PTC and LUM). Very few “dynamic” experimental data are available, and they relate to in vivo tissue and/or vascular PO_2_, with different protocols and methodologies (e.g. [71]). To the best of our knowledge, the only available data are summarized in SM1, for the (micro-)vascular compartments [56,72], and for the average cortical tissue [11,73]. Despite the important gap between the above experimental conditions for reference values and the model level, RCM proved able to reproduce quite well (within experimental range) ischemic and hypoxemic half-times), both in terms of average tissue and in terms of micro-vessels PO_2_. Importantly, Hb omission accelerates oxygen ischemic decreased 5-fold in the tissue, 10-fold in PTC’s. These figures cannot be “validated” per se due to the absence of experimental determinations. For the same reason, RCM anoxemic half-times, although accelerated in the absence of Hb, cannot be validated either. Finally, RCM simulated ischemic time to reach 10% of initial PO_2_ (~17 s) proved reasonably comparable to experimental data, both for tissue and for vessels [56,72]. Overall, RCM dynamic behavior corresponds well to available data. With regard to the limited contribution of hemoglobin on model PO_2_ (in reference conditions), it is important to stress that Hb omission consistently accelerated equilibration transients. At this point, RCM was considered validated, both under steady-state and transient conditions.

### 4.5. Capillary and Hemoglobin Versus Luminal Oxygen Delivery

Two related and counter-intuitive observations were made with RCM, which seemingly contradicts the usual capillary and hemoglobin paradigms in terms of oxygen delivery. First, hemoglobin exerts a weak influence on sstPO_2_ under reference conditions. A priori counter-intuitive, this model-based observation parallels the known excess oxygen supply to the kidney, particularly to the cortex. At RBF°, the capillary flow is ~23 nL/min, equivalent to a PTC residence time of ~2 ms. Based on this important capillary variable, we reasoned that the a priori expected Hb contribution to model PO_2_ could be “unmasked” by increasing residence time. Indeed, we observed that below 2.0 FU (8 nL/min, RT ~5 ms), Hb contribution to tissue PO_2_ started to increase, to reach ~12 mmHg. Other manipulations, such as increasing QO_2_ (2-fold), did not modify ΔHb. Second, as already mentioned, under normal perfusion, about 80% of model PO_2_ is determined by tubular lumen, whereas capillaries contribute ~20%. PTC contribution progressively takes over when RBF is decreased. These exploratory observations and verifications proved robust with respect to tissue geometry and parameters (data not shown). In any case, they likely relate to capillary “blood” (or erythrocytes) transit time. Interestingly, to the best of our efforts, we could not find any experimental demonstration that the presence of Hb in normal kidney perfusion is associated with higher values of tissue PO_2_.

### 4.6. Can Really Luminal PO_2_ Delivery be of Significance?

In view of the above, after numerous verifications, this question became pregnant, even more so because, again to the best of our knowledge, the possibility of any significant oxygen delivery via the tubules has been largely ignored. This is surprising, at least for two reasons. First, bibliographical analysis shows that oxygen is present in the tubular lumen, at levels comparable to micro-vessels and capillaries, 30–50 mmHg according to [52,53,54] (SM1-Table S1). What would prevent such dissolved oxygen to diffuse toward epithelial cells? Second, because urinary PO_2_, well known to be far from zero (in the 10–20 mmHg range) is considered as a reasonable, measure of medullary PO_2_ [74,75].

To repeat ourselves, the standard view about tissue oxygenation, is merely that capillaries and hemoglobin are the sole oxygen providers. In the case of the kidney, it might be an “a priori” requiring to be demonstrated. Our model-based observations suggest that it may not be the case. In their model of “cortical oxygenation”, Lee and et al.’s model only allows capillaries to deliver oxygen (see Figure 2 in [26]); incidentally, the possible advective removal of oxygen by the flowing tubular fluid is also ignored. Simple calculations, ignoring PTC O_2_ delivery and using reference values (plasma sodium 142 mM, SNGFR 33 nL/min, luminal PO_2_ 40 mmHg) yield a single nephron Na^+^ load of 4.5 nmoles/min, versus a simultaneous oxygen load of 0.002 nmoles/min per glomerulus. As such, this ~2300 mole-to-mole ratio certainly does not support luminal PO_2_ as a possible oxygen source. In a recent commentary, Evans and Ow mention that glomerular filtrate oxygen contributes to less than 1% to epithelial oxygen tension [76]. This could be true; but most probably not always and not everywhere along the tubule. The calculations developed below support this notion. Taking into account a 15-factor (3 Na^+^/ATP for the Na^+^-K^+^ pump, and 5 ATP/O_2_ for Oxphos) reduces the luminal Na^+^ load/O_2_ load ratio to ~160; considering the average cortical transcellular/paracellular epithelial reabsorption fractions (0.81/0.19) and the bicarbonate effect” (cortical average, ~1.15 [14,46]) further reduces the ratio to ~110 Na^+^ per O_2_—still largely unfavorable. The total reabsorptive work-load is distributed, although not equally, all along the tubule length (~10 mm, for the proximal tubule). Based on the segmental distribution of maximal Na^+^-K^+^ pump activity (PCT/PR or S1/S2/S3 [46,77,78,79,80]), the average weighed fractional tubular reabsorption amounts to ~25% per millimeter. In other words, each millimeter of PT would have a minima 1 available O_2_ to reabsorb ~11 Na^+^ —still insufficient. But since the lumen-epithelial exchange surface area (brush-border membrane, dedicated to reabsorption) is at least 10-fold higher than the capillary-epithelial contact, the lumen-to-epithelia O_2_ diffusive fluxes are in fine comparable to the capillary-epithelial exchanges (all other factors considered equal). This analysis substantiates the notion that the luminal delivery of oxygen cannot be ignored and can even be substantial. Unplanned, the notion progressively imposed itself. Quite logically, this is exactly what we observed with RCM, whatever parametric/input configuration. Finally, the predominant control of tissue PO_2_ by luminal versus capillary PO_2_, appears directly related to quasi-independence of tissue PO_2_ versus hemoglobin (in reference conditions).

Again, these observations came out as totally unexpected because a priori, we all had the “common sense” notion that hemoglobin provides oxygen to consuming tissues, such as muscles or brain (e.g., [81,82,83])—even though, of course, it is the dissolved oxygen which in fine diffuses towards the tissue. We were so puzzled that we performed extensive, additional verifications, including numerous controls and verifications of hemoglobin equations, parameters and behavior (see above). In all cases, Hb exerted practically no influence upon tissue PO_2_ (<0.1–0.2 mmHg).

In order to confirm that our observations were indeed not artifacts, and specific of the renal cortex, we constructed “muscle-oriented” variants of RCM. These “muscle models” were designed with the same capillary density as their renal counterpart; in addition, i) all luminal patches were replaced by “epithelial” patches, and ii) all “epithelial/muscle” patches were attributed sub-maximal muscle QO_2_ (1.0–5.0 mM/min; [82,83,84,85,86]). Finally, the capillary factor was adjusted so that apparent blood velocity ranged within muscle reference values (0.1 to 2.0 mm/s [82,87]. Under these muscle-like settings, hemoglobin contribution to tissue PO_2_ was ~2–8 mmHg. To the best of our research, no equivalent renal tissue PO_2_ measurements in the absence of hemoglobin was found. Of note, even at the “high” 5 mm/s apparent velocity (perfusion “RBF” set at 5 FU, capillary adjusting factor set at 1.0), Hb contribution was already increased 5–10 fold in “muscle-model” vs. RCM (to ~1.0 mmHg). No further attempt was made here toward a more detailed representation of muscle tissue. Certainly, the emergence of a strong Hb control over tissue PO_2_ (as seen in RCM and “muscle-models”) relates to the capillary residence time (typically 100–1000 ms), a quantity driven by the apparent blood velocity. It is well known that the capillary transit time constitutes a kinetic barrier to oxygen delivery, and that the higher its value the higher the oxygen delivery [45,65]. With RCM, this effect was seen in all parametric configurations tested (see below). A final word of caution. As mentioned, RCM is parameterized from bibliographical references, most of which relate to outer cortex. In addition, at this stage of its development and because of its simple 2D geometry, RCM averages segmental tubular characteristics and does not allow for a more accurate dissection of luminal oxygen delivery. For instance, should the early parts of PCT (the so-called S1 segment, about one third of PT length) exhibit a higher fractional reabsorption than the average value considered above [46,77,78,79,80], it would consume in proportion more oxygen, leaving less for subsequent segments.

It remains that our model and the above reasoning strongly suggest that the bulk of normal renal cortical oxygen originates from filtered oxygen at the glomerular level, not from capillaries. This is perhaps not that surprising, if one considers that the main physiological function of cortical peritubular capillaries is to reabsorb the enormous amount of proximal filtrate (water, NaCl, bicarbonate etc.). Perhaps less obvious is the suggestion that in doing so, perhaps because of doing so, peritubular capillaries do not provide much of the oxygen required for this reabsorption, a task seemingly devoted to the tubules. One incident advantage of such a situation would be that more capillary (Hb bound) oxygen would be thus available for the hypoxic medulla (see [30,88]).

### 4.7. Model Simplifications, Limitations, and Issues

#### 4.7.1. RCM is a Simplified 2D Description

RCM is a 2D, multi-capillary, modified Krogh’s model [81]. Its 2D geometry is the first and most important simplification. Exhibiting an apparent disorder (e.g., “cortical labyrinth”), the cortical tissue is complex and heterogeneous, even more so if compared to the highly structured medulla [21,27,29,30]. This is why, in this ab initio development, we did not embark into 3D tubulo-vascular cortical “reconstruction”—which, to the best of our knowledge, no study addressed up to now. The 2D representation and limited thickness prevent the accounting for known (and putatively consequential to oxygen distribution) axial PO_2_ gradients [45,81]. In addition, the topological continuity along tubules and capillaries is broken. We performed exploratory simulations with axially, parallel configured tubules and capillaries, still two-dimensional. Although not as accurately parameterized nor verified as was RCM, all simulations attempts with these “longitudinal cortical models” (30–40 mmHg average PO_2_) ended at confirming the low control of Hb and PTC over average tissue PO_2_ (data not shown). It nevertheless remains that the limited length for “convection” imposed by the 10 µm-thickness of RCM most likely overestimates the contribution ratio of tubules to capillaries in terms of oxygen delivery. Finally, RCM likely represents more accurately the outer and mid cortex, than the deep cortex and the cortico-medullary junction. Since IR-induced necrosis is known to develop radially, from the CMJ toward the capsule it will be of utmost importance to specify and parameterize this key area. At this point, several of the required experimental data are missing to confidently do so.

#### 4.7.2. RCM Represents Capillaries but Does Not Implement Capillary Hemodynamics nor Circulating Cells, Including Erythrocytes

We partially addressed this limitation with the “capillary hemodynamic factor” allowing to impose more physiological capillary “blood” velocities (see above). The conclusions are drawn from this crude, phenomenological correction obviously call for physiologically-based modeling of the utterly complex capillary hemodynamics, taking into account local “tube” hematocrit, viscosity, flow, velocity, and dimensions. Moreover, the mechanical (deformation) and biochemical (ATP release, Hb/NO°, etc.) interactions between erythrocytes and the capillary/endothelial wall, all known to facilitate oxygen delivery, are ignored in our primitive construct [89,90].

#### 4.7.3. RCM Features Non-Resistive, Non-Pressure Driven Flows

Our model does not implement mechanic nor hemodynamic phenomena: blood and luminal flows are not resistive (i.e., not pressure-driven), tubules are not compliant [33], and hydrostatic and oncotic pressures, as they drive reabsorption towards the capillaries, are ignored. When perfusion is stopped and filtration ceases, local reabsorption can proceed for some time (as occurs in RCM), perhaps 10–15 s (see Discussion in [11]), but, in the same time, tubules and capillaries will collapse, which does not occur in current RCM. The omission of flow-pressure relationships has another consequence: simulations performed at decreasing perfusion, do so by ignoring the reduction of perfusion pressure, thus assuming that the glomerular filtration pressure remains above its cut-off pressure (~18 mmHg). In situ, below such threshold, capillary perfusion (and associated oxygen delivery) can persist while luminal flow is arrested. From this standpoint too, RCM likely overestimates the contribution ratio of tubules to capillaries in terms of oxygen delivery, although this restriction does not apply at normal RBF.

#### 4.7.4. Renal and Systemic Regulations Are Missing

Renal regulations are purposedly missing, including autoregulation and tubulo-glomerular feedback, and by definition systemic influences. Such simplifications set limits to the conclusions that can be drawn, particularly from RCM-simulated ischemia.

#### 4.7.5. RCM Ignores Other Cortical Potential Sources of Oxygen

RCM ignores arterioles and glomeruli, and higher-order vessels as well, structures known to exhibit PO_2_ closer to arterial PO_2_ (~100 mmHg), in any case markedly higher than the “average cortex”. Because the corresponding PO_2_ gradient would be favorable, it can be argued that such potential sources could indeed deliver oxygen to nearby consuming tubules (see [26]). In RCM, we explored the influence of such oxygen sources by setting the PO_2_ variable of selected patches (up to 5 × 5 regions) to the range of reported values (50–100 mmHg). These exploratory simulations showed that the influence of such high oxygen sources restricted to less than 1–2 mmHg, within a 30 µm-radius (single patch or multiple-patch area). Identical observations were made when neighboring consumption was either drastically reduced or increased. Similarly, the local “closing” of a given capillary (simulating a local thrombus or nearby leukocyte adhesion), or for that matter of an entire tubule (simulating intratubular casts), did not extend its influence upon oxygen farther than its immediate neighbors (~10–30 µm). This observation was to be expected, as a direct consequence of oxygen diffusion physics, and it has been confirmed by others that the influence of oxygen sources, including capillaries, ranges 20–30 µm [91].

#### 4.7.6. Defective Validation Versus Luminal and Interstitial Measurements (One Study)

Notwithstanding experimental limitations to this seminal and important study, the defective validation versus luminal and interstitial PO_2_ measurements [54], stresses that RCM, as a model, is uncomplete. As mentioned, RCM currently “average” renal cortical components, both “horizontally” (PCT, PR, DT) and “vertically” (superficial, median and juxtamedullary nephrons). A more specific, region and segment-based parameterization, or a 3D construct should improve RCM discriminating power. Experimental data would be surely missing, although new and more accurate oxygen measurement methodologies (e.g., segment-specific) are becoming available (see [92,93]).

### 4.8. Perspectives and Conclusion

#### 4.8.1. Time Scale, Energetic Metabolism and Tissue Response to IR

RCM was developed to address oxygen dynamics in time ranges pertaining to convection, diffusion, and consumption (ms to seconds), at the histological scale (tens of µm). Such a short time scale cannot address IR events, which develop in minutes (ATP drop or endothelial activation), hours (leukocyte infiltration, cell death), days (tissue repair) and weeks/months to years (fibrosis). As an anticipation, an oxygen steady-state, ATP dynamic, version of RCM has been implemented, able to run with 5 to 60 min time-step, and yielding similar PO_2_ as the present model, within 2–3 mmHg, across the whole ranges of inputs. Finally, RCM does not cover the regulatory processes allowing the kidney to react and adapt to ischemic/hypoxic events. RCM refinement with the addition of more detailed cellular functionalities such as oxidative/nitrosative stress and cell/death/repair is a key requirement to IR and IRI description.

#### 4.8.2. Towards Renal IR Injury

As a whole, our results suggest that in the renal cortex the “oxygen feeding unit” for a tubule, is actually constituted by its own lumen and nearby capillaries. This is different from other, more “structured” organs such as the striated muscle, in which the feeding unit “restricts” to capillaries [81,84,94]. In this reasoning, it appears that unless it is severely widespread, tissue fibrosis will not be a major impediment to oxygen delivery at the local scale—as long as tubular (and perhaps capillary) flow is sufficient, i.e., as long as glomerular function and filtration is maintained. With RCM, we performed exploratory simulations supporting this idea (by manipulating histological diffusion coefficients), but this is an important point, that requires to be specifically addressed, and which will strongly depend on the fibrosis-inducing etiology (e.g., glomerular vs. tubular diseases).

It is often hypothesized that capillary rarefaction and/or fibrosis, both deleterious consequences and hallmarks of renal IR injury, increase diffusion distance and reduce oxygen availability [95]. From our observations, it would appear that, at least at the local scale and in “normal” conditions, and, again, as long as the “tubule/capillaries” couple is present and properly perfused, relevant tubular cells will be oxygenated and the transepithelial reabsorptive processes will occur. It has been observed in IR-related renal pathologies that glomerular degeneration, tubular atrophy, and interstitial fibrosis, correlate with capillary rarefaction [96,97]. This suggests that local hypoxia observed with capillary loss (as in [96], with pimonidazole) might not be due to capillary rarefaction, but to some reduction in tubular flow. RCM simulations, ignoring etiology and regulations, nevertheless yield a quantitative effect of luminal flow, amounting to 10–30 mmHg, far sufficient for chemical detection with pimonidazole [98].

#### 4.8.3. Hypothermia and Oxygen Carriers

With oxygenation, temperature is one key parameter in renal graft preservation. Based on cell metabolic reduction, hypothermic storage is the main strategy to minimize ischemic (and/or reperfusive) injuries [3,99]. RCM hemoglobin stabilizes cortical oxygen and slows the ischemic O_2_ disappearance; exploratory experiments where parameters and processes were adjusted at their 4 °C—equivalent showed that low temperature stabilizes tissue oxygen. Thus RCM could also be used as a helping tool to analyze the role of temperature on the one hand, and hemoglobin and various other oxygen carriers, on the other hand [3,99].

#### 4.8.4. Erythropoietin

The low control of cortical PO_2_ by hemoglobin may appear surprising, considering that the kidney is the main producer of erythropoietin (Epo), in adults [100,101], and that the specific stimulus for Epo expression is considered a reduction in local PO_2_ (cell/tissue?) [101]. However, renal perfusion and hormonal influences have been also been shown to be at play, if not determinant in cases. Moreover, the specifics (amplitude, kinetics and exact localization) of Epo production are still unraveled, especially in terms of local PO_2_, despite the identification of specialized interstitial fibroblasts, the so-called renal Epo-producing (REP) cells, localized in the juxta-medullary region of the cortex, CMJ [102,103]. Current RCM does not target this region. We hypothesize that, when properly unraveled, the CMJ region will show a local, specific regimen of oxygen regulation. Certainly, juxta-medullary model variants would be of help in that matter.

### 4.9. Conclusions

We have developed and validated RCM, the first dynamic model of renal cortical oxygenation at the cell-tissue level. Consistently parameterized for the rat, RCM is more of a “knowledge model” at this stage, but it already helps a better and quantitative understanding of cell-tissue level interactions involved in local oxygen delivery and consumption. As such, it will allow for the examination of mechanistic or etiologic hypotheses. Within acknowledged limits, RCM simulations lead us to propose the challenging hypothesis of a significant contribution of luminal oxygen to cortical oxygenation. Along with progressive refinements, RCM will constitute a quantitative tool to address IR-related pathophysiological and therapeutic aspects. Finally, RCM could constitute a basis for tissue-level modules required for multi-scale/multi-dynamic models addressing renal function, at the organ or systemic levels [102,103] during and after ischemia-reperfusion events.

## Figures and Tables

**Table 1:** Principal variables and parameters used in RCM (reference setting, RCM°).

Name	Description	Value	Unit	SM1 Table
RBF	Renal blood flow	5.3	mL/(gkw.min)	Table S1
PTC PO_2_	Capillary PO_2_	56	mmHg	Table S1
LUM PO_2_	Luminal PO_2_	40	mmHg	Table S1
FF	Filtration fraction	0.34	-	Table S2
Nb	Nephron number	32,400	-	Table S2
Rc	Capillary radius	5.0	µm	Table S3a
Tub-length	Tubule length	10,400	µm	Table S3a
Bbmf	EPI-LUM surface area	2000	µm^2^	Table S3b
ppsa	EPI-EPI surface area	800	µm^2^	Table S3b
pcsa	PTC-EPI surface area	157 *	µm^2^	Table S3b
pcsint	PTC-INT surface area	39 *	µm^2^	estimated
frReab	Fractional reabsorption	0.51	-	Table S4
frTransC	Na Transcell. fraction	0.81	-	Table S4
P/0	Oxphos ATP/O_2_	4.5	-	Table S4
Km-Cox	Km for O_2_ consumption	1.1	µmol/L	Table S4
Hta	Arterial Hematocrit	0.45	-	Table S6
Nap	Plasma Na^+^	142	mmol/L	Table S6
Hb4-RBC	RBC Hb concentration	5.2	mmol/L.rbc ***	Table S6
P_50_	Hb half-saturation PO_2_	36.8	mmHg	Table S6
DO_2_-PTC	Diffusion constant	1.40 × 10^−5^	cm^2^/s	Table S7
DO_2_-LUM	Diffusion constant	2.80 × 10^−5^	cm^2^/s	Table S7
DO_2_-EPI	Diffusion constant	1.10 × 10^−5^	cm^2^/s	Table S7
DO_2_-INT	Diffusion constant	2.20 × 10^−5^	cm^2^/s	Table S7
alpha	O_2_ solubility	1.34	µM/mmHg	Table S8
fBIC	HCO_3_-factor **	1.15	--	--

*, at Rc = 5.0; nb: the PTC-EPI and PTC-INT exchange surface areas (ESA) have been adjusted to 2-fold and 0.5-fold (with respect to the 100 µ^2^ reference ESA), respectively, to represent the fact that capillaries are dominantly in contact with tubules, not with the interstitium (see text). **, the “bicarbonate factor” implements the fact that, in early sections of the convoluted proximal tubule (segments S1 and S2), the basolateral Na^+^–3HCO_3_^−^ cotransport system increase Na^+^ reabsorption by up to 50%, independently of the Na^+^–K^+^- pump, at no ATP or oxygen cost, [14,22,46]. ***, red blood cells.

**Table 2:** Influence of main parameters on tissue PO_2_ output.

Parameter	Description	Parameter-Dependent Error (mmHg)
frReab	Fractional reabsorption	3.1 (−)
HKQO2EPImax	Maximal rate of basal QO_2_ * in EPI	3.0 (−)
frTransC	Transcellular Na reabsorption fraction	2.3 (−)
bbmf	Brush-border membrane factor	0.7 (+)
fPCSA	Surface area factor for PTC	1.0 (+)
DO2-EPI	Diffusion coefficient in EPI	1.0 (+)
ATP-O2	Oxphos ATP/O_2_ ratio	1.4 (+)
fBIC	HCO_3_^−^ factor *	1.4 (+)
alpha	Tissue oxygen solubility	1.7 (+)
Nc/Nt	Capillary/Tubule ratio	1.9 (+)
Rcap	Capillary radius	2.5 (+)
Tub-length	Tubule length	2.9 (+)

*, QO_2_, oxygen consumption.

**Table 3:** Adjusted parameters used in RCM* vs. RCM°.

Name	Description	RCM°	RCM*
Tub-length (µm)	Total tubule length	10,400	12,400
frReab	Total fractional reabsorption	0.51	0.75
frTransC	Na Transcellular reabsorption fraction	0.81	0.56
fBIC	Bicarbonate-factor	1.15	1.5
P/0	Oxphos ATP/O_2_	4.5	5.5
	**Model output PO_2_ (mmHg)**	**34.2**	**35.9**

RCM° and RCM* respectively refer to the reference and the physiologically-adjusted parametric configurations (see text).

## Supplementary Materials

Supplementary Materials can be found at https://www.mdpi.com/1422-0067/20/24/6246/s1. Supplementary Materials File 1 (SM1); Supplementary Materials File 2 (SM2).

## Abbreviations and Symbols Used

ABM	Agent-based modeling
AKI	Acute kidney disease
AV	Arterio-venous
BV (BV°)	Biological (or Bibliographical) value (reference BV)
CBF	Cortical blood flow
CKD	Chronic kidney disease
CFF	Capillary flow factor
CMJ	Cortico-medullary junction
CVF	Capillary volumic flow
DT	Distal tubule
EPI	Epithelial patch
EPO	Erythropoietin
ESA	Exchange surface area
fCapBr	Capillary branching factor
FF	Filtration fraction
frPR	Fractional proximal reabsorption
GFR	Glomerular filtration rate
GUI	Graphic user interface
Hb	Hemoglobin (free of oxygen)
HbO_2_	Oxygenated hemoglobin
Hbt	Total hemoglobin
Hta/e	Hematocrit (afferent/efferent)
HK	House-keeping
INT	Interstitial patch
IP/DP	Independent/Dependent parameter
IR	Ischemia-reperfusion
IRI	Ischemia-reperfusion injury
k′c	Association coefficient for the Hb/0_2_ reaction
kc	Dissociation coefficient for the Hb/0_2_ reaction
LUM	Luminal patch
MS	Modeling and simulation
Nap	Plasma Na^+^ concentration
nHill	Hill’s cooperativity index
N_tot_	Nephron density
Oxphos	Oxidative phosphorylation
OpNe	Operational nephron
P_50_	Half-saturation PO_2_ for hemoglobin
PO_2_	Oxygen partial pressure
PT	PT, proximal tubule
PCT	PCT, proximal convoluted tubule
PTC	PTC, peritubular capillary patch
PU	Patch units
QKDB	Quantitative Kidney DataBase
QO_2_	Oxygen consumption
RBF	Renal blood flow
RCM	Renal cortical model
RSC	Relative sensitivity coefficient
SNAPR	Single-nephron absolute proximal reabsorption
SNEABF	Single-nephron efferent arteriole blood flow
SNFLNa	Single-nephron Na^+^ load
SNGBF	Single-nephron blood flow
SNGFR	Single-nephron GFR
sstPO_2_	Steady-state average model PO_2_
TFF	Tubular flow factor
TNa	Na^+^ transport
TVF	Tubular volumic flow
Vcap	Capillary patch volume
Vlum	Luminal patch volume
