# Supplementary material for: A Computer Model of Oxygen Dynamics in the Cortex of the Rat Kidney at the Cell-Tissue Level
Source: Int J Mol Sci. 2019 Dec 11;20(24):6246. doi: 10.3390/ijms20246246 (PMC6941061; doi:10.3390/ijms20246246)
Supplement: Supplementary file 1 [file ijms-20-06246-s001.zip › ijms-596297-SI-to conversion/ijms-596297-Supplementary Materials File 1-SM1-to conversion.pdf]

International Journal of
Molecular Sciences

Supplemental Materials File 1—SM1

Section 1. Bibliographical analysis
Section 2. Biological values
Section 3. Parameter sensitivity and input-parameter analyses
Section 4. References

1. Literature analysis, parameters and variables

1.1. Bibliographic analysis and exploitation

The first step to construct a realistic rat kidney cortical model (RCM) was to harvest rat-specific functional, anatomical and morphometric data that. Search, extraction, and conversion of biological values was performed manually. We report them in Tables S1–S8, together with references. All values refer to 37 °C, unless otherwise specified. On the average, six bibliographical references and fifteen "determinations" per reference value were extracted (BV°). All Rat strains were considered, although the experimental corpus is dominated by Sprague-Dawley, Wistar and Münich strains. Only studies performed on adult, healthy rats were considered; no distinction was made between male and female.

As a whole, more than one hundred biological values were extracted from 280+ references, used as inputs, outputs or parameters for model construction, verification and validation. In SM1 Tables 1-8, the main 81 figures are reported as average, "normal" (or reference) biological values (BV°), with their standard deviation (SD), range, number of determinations (*n*), and number of sources (*N*), as well as corresponding references (see Methods). For clarity, these figures are categorized in 8 tables: inputs/outputs (Table S1), cortex: nephrons and flows (Table S2), morphometry (Tables S3a,b), energetics and transport (Table S4), non-epithelial consumption (Table S5), blood and hemoglobin (Table S6), oxygen diffusivity (Table S7), and oxygen solubility (Table S8).

Bibliometric yield an average $N = 6$ references (range 1–70, mode 3) per biological value, with average n = 83 determinations (range 1–2215, mode 20). Excluding arbitrarily the 14 BV's for which n > 50 (e.g., n = 2215 for cortical PO_2; Table 2), statistics yield an average of n = 15 ± 12 [1–45] per BV (mode 20). Tables S1–S8 feature more than 160 individual references. Several of them provide more than one BV, with an average of 1.6 BV per reference.

BV° were used for model debugging and tuning, and as base-case values; experimental ranges were used to determine the range of sensitivity analysis. Finally, within the model, the biological values can be categorized as follows: 43 independent parameters (IP), 64 dependent parameters (DP), and 3 inputs and 1 output. Because of space limitations, we do not analyze all reference-based BV's; instead, we detail below model inputs/outputs (I/O) on the one hand, and selected independent parameters, on the other hand.

1.2. Reference values for inputs and outputs

1.2.1. Inputs

The Rat RCM has two oxygen inputs: the renal blood flow (RBF), ultimately distributed to capillary patches (PTC) *via* capillary flow (CVF variable, Equation (7)), carrying O_2 (as Hb bound and dissolved), and the luminal tubular flow (RVF variable, Equation (8)), carrying only dissolved oxygen. Table 2 (upper part) summarizes results from the bibliographical search required for RCM inputs.

The "RBF" input (renal blood flow) refers to a single kidney blood flow, as extracted from litterature; since the model concerns the "cortical area", the *RBF* input is scaled by the "fractional cortical" BF, frCBF (Equation (1)), an independent parameter. The RBF reference value, RBF°, is 5.3 ±

1.0 mL.min^{-1}.gkw^{-1}, or Flow units FU (2.1-12.0 mmHg; n = 182; N = 11 sources, corresponding to a total of 32 original studies:

- three original studies measuring RBF (*n* = 6 in [1]; *n* = 17 in [2]; *n* = 18 in [3]);
- two reviews, [4,5], analyzing *N* = 6 and 9, respectively, "rat" studies for RBF;
- the *Quantitative Kidney DataBase*, QKDB, from which we extracted *N* = 14 studies reporting RBF value in healthy rats [6]; please note that the QKDB link that we used seems to be unavailable as to august 2019.

The second model input is the "feeding PO_2", used to calculate the amount of oxygen delivered to the cortical slice model via both PTC patches (equations 11 and 12) and luminal patches (equations 13 and 14). However, we average all these as reference input for "vessels" (µvPO_2° = 56 mmHg, Table 1). Further, we provisionally define reference feeding PO_2 as the average of luminal tubular (40 ± 6 mmHg, range 34-49, *n* = 16, *N* = 2) and "microvascular" (56 ± 14 mmHg, range 38–82, *n* = 45, *N* = 4; Table 1): in Sensitivity analysis and (the first part) of *I/O* analysis, this 48 ± 10 mmHg figure will be used as the reference value for both PTC and luminal patches feeding PO_2 (vide infra). As a verification, the detailed anatomy-based (see [7]) mathematical model of arterio-venous shunting in the Rat renal cortex by Gardiner and coworkers, suggest that PO_2 in the last vascular segments of the arterial tree are in the 50-70 mmHg range [8,9] (Table S1). The consequences of applying different PO_2 feeding values to capillaries and lumen will be addressed in I/O analysis. Together with reference values for independent parameters (as defined in the following section), reference inputs RBF = 5.3 FU and PO_2 = 48 mmHg (LUM = PTC), will constitute RCM reference configuration (referred to as RCM°).

1.2.2. Outputs

Bibliography-based expected outputs for our cortical model are summarized in Table S1 (lower part). The main RCM output is the average, steady-state cortical "tissue pressure", sstPO_2 calculated as the average PO_2 of the 900 "central" patches (i.e., excluding 124 edge-patches in order to reduce border effects). Juxtamedullary nephrons ("deep nephrons", approximately in the last mm of the 3mm-deep Rat cortex; [10]) represent "only" 6–25 % of all nephrons (Table S2); moreover, the inner cortex is in contact with the outer medulla which presents a 24 ± 12 mmHg PO_2 (range 12–42, [2], see also [10]). As a consequence, the inner cortex exhibits a much lower PO_2 than the outer cortex, in the 23-37 mmHg range (icPO2° = 29.8 ± 1.3 mmHg, Table 2). Thus, RCM output sstPO_2 will be compared to "outer cortex PO_2", ocPO_2 ° = 40.8 ± 6.2 mmHg (range 21–64, Table S2). This is examined in Results. As mentioned, the main RCM output that we exploit is a steady-state value. However, one important feature of our construct is that it is a dynamic model, effectively calculating transients. Thus, we extracted from the experimental literature a dynamic feature, that we call "ischemic cortical oxygen half-life" (t_{50}°, Table 1), defined as the time required for oxygen pressure to reach half of its reference value when input (RBF) is set to zero. This will be used as an additional validation for our model (see Results).

1.3. Reference values for parameters

Among the IP's, six are patch "variables": 4 patch diffusion coefficients, and epithelial and capillary house-keeping consumptions (HK-QO_2-EPI and HK-QO_2-PTC). Among the 64 *DP's*, 31 are patch variables, e.g., oxygen concentration, PO_2, and oxygen consumption rate. The IP/DP compendium is given in Tables S1–S8, as reference biological values (BV°). BV° of dependent parameters are used for internal consistency checking and model verification (see below, *Model verification*), while reference values of IP's are used to instantiate RCM.

Whenever relevant, cross-consistency is verified: as an example, EABF° (152 ± 29 nL/min, independently extracted from literature) is consistent with GBF° (224 ± 93 nL/min) minus SNGFR° (33±12 nL/min), both independently extracted from literature (Table S3). Because of space limitations, we do not comment all BV°; rather, we illustrate the process with a few selected examples below.

1) Single nephron filtration fraction (SNFF° = 0.34 ± 0.06, Table S2) and arterial hematocrit (Hta° = 44.8 ± 3.9, Table S6) drive the conversion of glomerular blood flow (GBF) into the two model

volumetric flows which "convectively" feed capillary and luminal patches, i.e. efferent arterial BF (EABF) and single nephron filtration (SNGFR, Equation (5)), respectively. As a verification, Table 2 also give independent BV° of whole-kidney FF, FF° = 0.33, similar to SNFF°.

2) Outer radius of proximal tubule (orTub° = 21.5 ± 1.8 µm) and the capillary sections over tubular sections ratio (Nc/Nt = 1.6 ± 0.2, Table S4a), condition the number of tubular and capillary sections in RCM. The reference capillary radius (rCap°) is 5.7 µm (Table S3a; see SM1 for the independent verification based on Steinhausen et al., 1973 [11]). In RCM, rCap drives two dependent parameters: i) the capillary branching factor and thus RCM oxygen feeding via the capillary volumic flow (CVF, equation 7; see SM2 for oxygen consumption), ii) the actual volume of the capillary endothelium.

3) In energetics and transport (Table S4), fractional proximal reabsorption (frPR° = 0.51 ± 0.12 nL/min), whole-kidney O_2 consumption (wk-QO_2° = 5.3 ± 2.6), and the cytochrome oxidase apparent Michaelis–Menten constant for O_2 (Km,cox° = 1.1 ± 1.0 µM), directly influence oxygen consumption, especially when available oxygen diminishes. The bibliographical value for frPR° (0.51) is confirmed by computing APR°/SNGFR° (16.9/32.9 = 0.51).

4) Hemoglobin O_2 half-saturation pressure (P_{50}° = 38.6 ± 2.6 mmHg, Table S6) drives the total amount of oxygen carried by "blood" flowing through capillaries. No Rat value for the apparent "off" constant of Hb-O_2 complex (only bovine or human).

5) O_2 diffusion constant is a patch variable (each patch type has its own value) which conditions oxygen fluxes between patches; Table S7 reports average value for different biological tissues, in comparison with water and plasma. For LUM and INT patches, we naturally use "water/saline" (2.8×10^{-5} cm^2/s) and "interstitium" (2.2×10^{-5} cm^2/s) values, respectively. The choice to attribute values for EPI (1.1 $\times 10^{-5}$ cm^2/s) and PTC (1.41 $\times 10^{-5}$ cm^2/s) patches is explained in SM1. Oxygen (Bunsen) solubility in tissues drives dissolved oxygen concentration for a given O_2 partial pressure. Since there is no statistical difference between tissues and/or fluids, RCM uses the O_2 solubility mean value (αO_2° = 1.34 ± 0.27 µM.mmHg^{-1}; Table S8).

2. Compilation of Biological Values of the Rat Kidney and Cortex

SM1-Table S1. Inputs and outputs.

Description	**Acronym** [unit]	**Standard value** SD, range	***n* values, *N* refs**	**References**
		**Model inputs**		
Renal Blood Flow	**RBF** [mL.min^{-1}.gkw^{-1}]	**RBF° = 5.3** SD = 0.9 Range = [2.1–12.0]	*n* = 182, *N* = 50	[1–5], QKDB(Dzo04)
Partial O_2 pressure (vessels)	**µvPO_2** [mmHg]	**µvPO2° = 56** SD = 14 Range = [38–82]	*n* = 45, *N* = 4	[3,12,13]
Partial O_2 pressure (tubular lumen)	**lumPO2**	**lumPO2° = 40** SD = ~6 Range = ~[34–49]	*n* = 20, *N* = 3	[3,14,15]
		**Model outputs**		
		Static		
Cortical PO2 (Outer cortex)*	**ocPO2** [mmHg]	**ocPO2° = 40.8** SD = 6.2 Range = [21–64]	*n* > 2 × 10^3, *N* = 11	[2,10,15–21] **
Cortical PO2 (Inner cortex)*	**icPO2** [mmHg]	**icPO2° = 29.8** SD = 1.3 Range = [23–37]	*n* = 26, *N* = 4	[3,10,15,16]
		Dynamic		
Cortical tissue ischemic O_2 half-life	**t_{50}** [s]	**t_{50}° = 4.7** SD = 2.5 Range = [4–11]	*n* = 8, *N* = 2	[20,22]

Microvascular tissue ischemic O_2 half-life	t_{50}<br>[s]	**t_{50}° = 10.5**<br>SD = 4.1<br>Range = [6–25]	*n* = 6, *N* = 2	[13,23]

* OC defined as first mm in-depth; IC defined as last mm in-depth [10]; **, excluding from [10] highest arterial values (>65mmHg, see also [9]).

SM1-Table S2. Cortex, nephrons and flows.

Description	Acronym [unit]	Standard value SD, range	*n* values, *N* refs	References
		**Independent parameters**		
Number of nephrons	**N**<br>[1e^3.gkw^{-1}]	**N °= 32.4**<br>SD = 4.8<br>Range = [26–40]	*n* = 12, *N* = 11	[5,24–27]
Fractional cortical BF	**frCBF**<br>[-]	**frCBF °= 0.66**<br>SD = 0.13<br>Range = [0.50–0.88]	*n* = 6, *N* = 6	[2,28–31]
Filtration fraction	**FF (kidney)**<br>[-]	**FF °=0.33**<br>SD = 0.11<br>Range = [0.16–0.64]	*n* = 22, *N* = 16	[2,5],<br>QKDB
Single Nephron FF	**SNFF**<br>[-]	**SNFF °= 0.34**<br>SD = 0.06<br>Range = [0.16–0.52]	*n* = 28, *N* = 8	[32–38]
Fraction of JM nephrons	**frJM**<br>[-]	**frJM °= 0.18**<br>SD = 0.09<br>Range = [0.06–0.25]	*n* = 7, *N* = 4	[39–42]
		**Dependent parameters**		
Glomerular Filtration Rate	**wk-GFR**<br>[mL.min^{-1}.<br>gkw^{-1}]	**GFR °= 1.06**<br>SD = 0.34<br>Range = [0.4–1.8]	*n* = 21, *N* = 13	[2,3,26,43–46]<br>QKDB
Single Nephron Glomerular BF	**SNGBF**<br>[nL.min^{-1}]	**SNGBF °= 224**<br>SD = 93<br>Range = [68–414]	*n* = 31, *N* = 9	[5,47–49]
Glomerular Plasma Flow	**GPF**<br>[nL.min^{-1}]	**GPF °= 113**<br>SD = 28<br>Range = [65–145]	*n* = 6, *N* = 2	[48,49]
Single Nephron Efferent Arterial BF	**SNEABF**<br>[nL.min^{-1}]	**SNEABF °= 152**<br>SD = 29<br>Range = [120–202]	*n* = 94, *N* = 4	[35,48–50]
Single Nephron GFR (all Nephrons)	**SNGFR**<br>[nL.min^{-1}]	**SNGFR °(SN) = 32.9**<br>SD = 12.3<br>Range = [15,54] §	*n* = 196, *N* = 69	[5,26,51,52]<br>QKDB
Tubular volume flow (average)	**TVF**<br>[nl/min]	**TVF °= 16.7**<br>SD = 2.0<br>Range = [13–23]	*n* = 20, *N* = 3	[53,54], QKDB
End-Proximal Tubular Flow	**EPTF**<br>[nL.min^{-1}]	**EPTF °= 11.4**<br>SD = 4.2<br>Range = [4–16]	*n* = 7, *N* = 3	[55]<br>QKDB
Max-Proximal Transit Time	**maxPTT**<br>[s]	**maxPTT °= 12.3**<br>SD = 4.5<br>Range = [7–18]	*n* = 4, *N* = 4	[11,56]
Mid-Proximal Transit Time	**midPTT**<br>[s]	**midPTT °= 7.9**<br>SD = 2.6<br>Range = [4–10]		
Tubular velocity (linear)	**Vtub**<br>[mm/s]	**Vtub °= 0.75 mm/s**<br>SD = 0.32<br>Range = [0.4–1.5]	*n* = 106, *N* = 4	[57–60]
Capill velocity (linear)	**Vcap**<br>[mm/s]	**Vcap °= 1.1**<br>SD = 1.3<br>Range = [0.5–4.1]	*n* = 6, *N* = 3	[11,61,62]
Capillary flow	**CVF**<br>[nL.min^{-1}]	**CVF °(Rc = 8 µm) = 28.8**<br>SD = 12.0<br>Range = [10–45]<br>**-**<br>**CVF °(Rc = 6.5 µ) = 11.6**<br>SD = 4.5	*n* = 133, *N* = 1<br>-<br>*n* = 20, *N* = 1	[11]<br>-<br>[11]

Range = [5–19]

SM1-Table S3a. Morphometry.

Description	Acronym [unit]	Standard value SD, Range	*n* values, *N* sources	References
		**General**		
Kidney weight	**kw** [g]	**KW° = 1.29** SD = 0.46 Range = [0.7–2.3]	*n* = 20, *N* = 11	[3,4,36,63–66]
Cortical thickness	**Cth** [mm]	**Cth° = 2.9** SD = 0.7 [2.0–3.9]	*n* = 6, *N* = 2	[7,10]
Fractional glomerular volume	**frGV** [--]	**frGV° = 0.04** SD = 0.02 Range = [0.02–0.07]	*n* = 26, *N* = 4	[63,67–69]
Fractional vascular volume (cortex)	**frVV** [--]	**frVV° = 0.18** SD = 0.08 Range = [0.11–0.27]	*n* = 14, *N* = 3	[7,69,70]
		**Independent parameters**		
Fractional cortical volume	**frCV** [--]	**frCV° = 0.66** SD = 0.09 Range = [0.56–0.77]	*n* = 20, *N* = 4	[7,63,67,70]
PT radius (outer)	**orTub** [μ]	**orPT° = 21.5** SD = 1.8 Range = [15,30]	*n* = 64, *N* = 4	[68,71,72], U1082*
PT radius (inner)	**irTub** [μm]	**irTub° = 11.4** SD = 2.0 Range = [9–15]	*n* = 10, *N* = 3	[68,57,58]
PT cell height (wo BB [1])	**CH** [μm]	**CH° = 9.9** SD = 2.6 Range = [5–14]	*n* = 12, *N* = 5	[71,73,68,72,74]
Brush-Border Height	**BBH** [μm]	**BBH° = 4.3** SD = 1.2 Range = [2.3–5.8]	*n* = 6, *N* = 3	[68,73,75]
Capillary radius (all orders)	**rCap** [μm]	**rCap° = 5.7** SD = 2.6 Range [2–14]	*n* = 29, *N* = 5	[11,56,72,76,77], U1082*
Capill. section/ Tubul. section	**Nc/Nt** [--]	**Nc/Nt° = 1.6** SD = 0.2 Range = [1.1–2.2]	*n* = 17, *N* = 3	[78,79], U1082*
Proximal tubule length (total)	**LPT** [mm]	**LPT° = 10.4** SD = 2.7 Range = [6.9–14.7]	*n* = 249, *N* = 6	[39,57,58,73,80,81]
Proximal tubule length (convol.)	**LPCT** [mm]	**LPCT° = 5.7** SD = 1.1 Range = [4.0–6.3]	*n* = 70, *N* = 3	[25,82,60]
Distal tubule length	**LDCT** [mm]	**LDCT° = 2.0** SD = 0.3 Range = [1.5–2.3]	*n* = 5, *N* = 1	[83]
		**Dependent parameters**		
Capillary density	**CapD** $[mm^{-2}]$	**CapD° = 887** SD = 333 Range = [250–1110]	*n* = 45, *N* = 3	[8,84], U1082*
Fractional tubular volume (lumen included)	**frTV** [--]	**frTV° = 0.76** SD = 0.07 Range = [0.64–0.83]	*n* = 32, *N* = 6	[63,67–69,75], U1082*
Fractional capillary volume	**frCV** [--]	**frCV° = 0.09** SD = 0.03	*n* = 13, *N* = 4	[67,69,72,75]

(cortex)		Range = [0.05–0.12]		
Fractional interstitial volume	**frIV** [--]	**frIV° = 0.08** SD = 0.04 Range = [0.04–0.11]	*n* = 12, *N* = 3	[67,69,75]
Tubule length density (cortex)	**Lv** [m.gkw^{-1}]	**Lv° = 396** SD = 103 Range = [289–494]	*n* = 32, *N* = 3	[68,85,86]
Tubule surface density (cortex)	**Sv** [µm^{-1}]	**Sv° = 0.048** SD = - Range = [-]	*n* = 5, *N* = 1	[85]

* , unpublished data (N. Quellard, B. Fernandez, IRTOMIT and Anatomo-Pathology Dpt, Pr JM Goujon, CHU of Poitiers).

SM1-Table S3b. Exchange surface areas.

Parameter	Patch pair	SAF	ESA	Adjusted
ppsa	EPI-EPI (intra-tubular)	8*	800 µm^2	-
-	EPI-EPI (inter-tubular)	1	100 µm^2	-
bbmf	EPI-LUM (brush-border)	20 *	2,000 µm^2	-
pcsa	PTC-EPI **	0.79	79 µm^2	157 µm^2
pcsint	PTC-INT	1	100 µm^2	49 µm^2
-	LUM-LUM and INT-INT	1	100 µm^2	-

SAF, surface area factor (default = 1.0); ESA (effective surface area) = 100 µm^2 x SAF. * from Pfaller, 1980; ** at capillary radius Rc = 5 µm. NB: the PTC-EPI and PTC-INT exchange surface areas (ESA) have been adjusted to 2-fold and 0.5-fold (with respect to the 100 µm^2 reference lateral ESA), respectively, to represent the fact that capillaries are dominantly in contact with tubules, not with the interstitium.

SM1-Table S4. Energetics and transport.

Description	Acronym [unit]	Standard value SD, range	*n* values, *N* sources	References
		**Independent parameters**		
Whole kidney consumption	**WK-QO$_2$** [mM/min]	**WK-QO$_2$ °= 5.79** SD = 2.3 Range = [3.2–9.2]	*n* = 35, *N* = 5	[3,10,21,87,88]
Fractional basal O_2 consumption	**frHK-QO$_2$** [-]	**frHK-QO$_2$ °= 0.25** SD = 0.16 Range = [0.12–0.44]	*n* = 56, *N* = 3	[3,88,89]
Fractional proximal sodium reabsorption	**frPR** [-]	**frPR °= 0.51** SD = 0.12 Range = [0.27–0.65]	*n* = 54, *N* = 6	[32,90–94]
Fractional distal sodium reabsorption	**frDR** [-]	**frDR °= 0.09** SD = 0.06 Range = [0.03–0.20]	*n* = 12, *N* = 7	[83,95–97]
Fractional (proximal) transcellular Na^+ transport	**frCTNa** [-]	**frCTNa °= 0.81** SD = 0.20 Range = [0.53–1.00]	*n* = 14, *N* = 3	[98–101]
P/O ratio (Oxphos)	**P/O** [-]	**P/O °= 2.5** [2] SD = 0.5 Range = [2.0–3.0]	*n* = 12, *N* = 3	[102–104]
Cytochrome C oxidase Km for O_2	**Km,cox** [µM]	**Km,cox °= 1.1** [3] SD = 1.0 Range = [0.2–3.0]	*n* = 46; *N* = 34	[105,106]
		**Dependent parameters**		
Single Nephron Absolute Proximal Reabsorption	**SNAPR** [nL.min^{-1}]	**APR °= 16.9** SD = 3.8 Range = [9.0–20.3]	*n* = 29, *N* = 5	[32,90,93,107,108]
PCT/PR NaK-pump	**frNaKP** [-]	**frNaKP °~4.0** SD = -	*n* = 3, *N* = 3	[109–111]

rate ratio		[3.0-10.0]		
PT Na-K pump Maximal rate (Na+ flux)	**Vm(NaKP, PT)** [mM/min]	**Vm(NaKP,PT) = 396** [4] SD = 320 [125–750]	*n* = 4, *N* = 4	[109,112,113,110,111]
DT Na-K pump Maximal rate (Na+ flux)	**Vm(NaKP, DT)** [mM/min]	**Vm(NaKP, DT) = 796** [4] SD = 305 [521–1125]	*n* = 4, *N* = 4	[109,112,113,110,111]
PT transport metabolic efficiency	**TNa/QO_2**	**TNa/QO_2 °= 27.0** SD = 2.0 [24–30]	(-) *N* = 2	[98,99]

[1], all three *Rat* references reviewed and analyzed in [114]; [2], averaging values from succinate and NADH-fed mitochondria; [3], values reviewed (and determined) in [105] and in [106]; refer to 25–37°C (*n* = 24/46 values at 37°C), and various experimental models (*n* = 26/46 values for Rat, but no renal data); [4], mean value calculated along the appropriate segment.

SM1-Table S5. Non-epithelial oxygen consumption.

Description	Acronym [unit]	Standard value SD, range	***n*** **values,** ***N*** **sources**	References
		**Independent parameters**		
Capillary specific consumption rate	**$HK-QO_2-PTC$** [mM/min]	**$HK-QO_2-PTC$ °= 1.2** SD = 1.1 Range = [0.2–2.8]	*n* = 18, *N* = 3	[115–117]
Fibroblast specific consumption rate	**$HK-QO_2-FIB$** [mM/min]	**$HK-QO_2-FIB$ °= 1.4** SD = 1.3 Range = [0.2–2.8]	*n* = 3, *N* = 1	[118]

SM1-Table S6. Blood and hemoglobin.

Description	Acronym [unit]	Standard value SD, range	***n*** **values,** ***N*** **sources**	References
		**General**		
Arterial PO_2	**aPO2** [mmHg]	**aPO2° = 91** [80–104]	*n* = 30, *N* = 3	[2,3,10]
		**Independent parameters**		
Arterial hematocrit	**Hta** [%]	**Hta° = 44.8** SD = 3.9 Range = [41–52]	*n* = 21, *N* = 10	[1,2,37,39, 123–125]
Molecular weight (tetramer)	**MW** [g/mol]	**64,000**	-	[122]
Hb4 RBC concentration	**Hb** [g/dL] **Hb** [mM]	**Hb° = 33.5** [29–40] **Hb° = 5.2** [4.5–6.2]	- -	[123] AP, 2nd ed. 2005
Half-saturation PO_2	**P50** [mmHg]	**P50° = 38.6** SD = 2.7 [36–42]	*n* = 71, *N* = 4	[87,124–126]
Hill's index	**nH** [-]	**nH° = 2.4** SD = 0.2 [2.1–2.9]	*n* = 41, *N* = 3	[87,124,125]
Rate constant for HbO_2 dissociation	**k′ (or k_{off})** [s^{-1}]	**k′° = 28** SD = 17 [8–65]	*n* = 20, *N* = 3	[127–129]
Plasma Na^+	**Nap** [mM]	**Nap° = 142.6** SD = 3.3 [134–152]	*N* = 78, *N* = 2	[119,130]
		**Dependent parameters**		
Blood hemoglobin concentration	**HGB** [g/dL]	**HBG° = 15.1** SD = 0.93 [13.1–16.0]	*n* = 8, *N* = 4	[3,119,123,131]

*, mammalian values (not specifically Rat); Note: 1 mmHg (1 Torr) = 1.25 μM; 1 kPa = 7.5 mmHg = 9.37 μM (37°C).

SM1-Table S7. Oxygen diffusivity in tissues and fluids.

Description	Acronym [unit]	Standard value [exp. range]	SD n, N	References
		**General**		
Water/saline	**DO_2** [cm2/s]	**2.8x10^{-5}** [2.1–3.3]	**SD = 0.58** n = 18, N = 5	[132–136]
		**Independent parameters**		
Plasma		**2.1x10^{-5}** [1.3–2.8]	**SD = 0.5** n = 9, N = 7	[141–143,137, 144,145,139]
Blood [1]		**1.2x10^{-5}** [0.7–1.6]	**SD = 0.3** n = 11, N = 7	[134,135,140–143]
Tissue [2]	**DO_2** [cm2/s]	**1.7x10^{-5}** [1.1–2.4]	**SD = 0.5** n = 7, N = 4	[133,136,141,144]
Interstitium [3]		**2.2x10^{-5}** [2.0–2.4]	**SD = 0.2** n = 3, N = 2	[139,143,145]
Intracellular [4]		**1.4x10^{-5}** [0.5–2.2]	**SD = 0.6** n = 8, N = 7	[117,132,139,143,145–147]
Endothelial cell		**0.5x10^{-5}** [0.1–0.9]	**SD = 0.5** n = 2, N = 1	[117] [139,148]

All values (rounded to the nearest 1st decimal) unless specified correspond to 37 °C (explicitely mentioned in most cases). [1], "blood" value represent the average of: (i) hemoglobin solutions (n = 4, among which two are given at 25°C [134,142], still higher than the other two values, reported by Spaeth [135]), (ii) red blood cells suspensions (n = 1, [133]), and (iii) blood (n = 6, N = 6); [2], "tissues" value represent the average of non-muscle (n = 6), striated muscles (n = 1), and kidney tissue (n = 1, carcinoma, [133]); [3], "interstitium" value averaged from: (i) Vadapalli-2002 [139], (ii) review by Dutta and Popel [145], hypothesizing from measurements and theory that interstitium Krogh's permeability [the product of solubility and diffusivity] for oxygen should be close to saline, and (iii) Sharan-1992 [143]; [4], "intracellular" values estimated from: (i) Dutta and Popel, [145] (n = 2, ibid; citing Rumsey [149] and Jones et al., 1980: n = 2, cardiomyocytes), and (ii) Brown [132] (n = 1).

SM1-Table S8. Oxygen solubility in biological tissues and fluids.

Description	Acronym [unit]	Standard value range	SD n, N	References
**General**				
**Water** [1]	αO_2 [μM.mmHg^{-1}]	**1.35** [1.25–1.49]	**SD = 0.09** n = 7, N = 5	[139,142,145,150,151]
**Independent parameters**				
**Blood** [2]		**1.47** [1.39–1.56]	**SD = 0.09** n = 5, N = 4	[127,139,152,153]
**Tissue, cells** [3]	αO_2 [μM.mmHg^{-1}]	**1.31** [0.94–1.49]	**SD = 0.38** n = 8, N = 5	[121,142,149,156,157]
**Vessel wall**		**1.22** [0.95–1.75]	**SD = 0.46** n = 3, N = 3	[139,154,155]
**Mean**	αO_2° [μM.mmHg^{-1}]	**1.34** [0.90–1.75]	**SD = 0.27** n = 23	**N = 12**

All values rounded to the nearest 1st decimal; unless specified (see below), all values correspond to 37°C, either explicitely mentioned in reference from the corpus (mostly) or implicitely (rarely). [1], "water" value represent the average of water/saline buffer (n = 4) and plasma (n = 1); two values at 23-25°C (kept because in range); [2], "blood" value represent the average of red blood cells suspensions (n = 2) and blood (n = 1); [3], "tissue" value represent the average of "many tissues" (n = 1, [156], cited in [117]), cell/extracell (n = 1, [138]), "cytosol" or "tissue" (n = 2, Groebe90, citing [157]), and muscle fiber (n=2, [145,152]). One value at 40°C (1.94, in [145]).

3. Parametric sensitivity and input-parameter analysis

In order to best characterize model dependence with regards to reference values (extracted from our bibliographical corpus, see 4th section, and carrying experimental variability), we performed: i) parametric sensitivity analysis (SA), ii) input/output analysis (I/O), and iii) input-parameter interaction analysis.

This was done based on steady-state average tissue oxygen pressure, $sstPO_2$: $sstPO_2$ tolerance < 1×10^{-5} mmHg, typically reached in 1000–1300 ms simulated time from initial state (all patches initialized to zero oxygen).

3.1. Parametric sensitivity analysis

Exhaustive SA, i.e. scanning of all parameter-parameter interactions (so-called global SA), was not performed, because it would be unpractical and lengthy. We performed local SA, whereby the influence of one parameter upon the output variable ($sstPO_2$) is evaluated at a time, around its reference value, within experimental limits (see Tables S1–S8, above). On the basis of exploratory simulations and/or reasoning, from the whole set of model parameters, only 22 were specifically addressed in the SA. Based on the relative sensitivity coefficient (RSC, Figure S1B below), two main groups appear: i) parameters that are influential with RSC ranging 1.0–3.0 (positive for those related to oxygen delivery, negative for those related to consumption), and ii) uninfluential parameters with RSC 0.0–0.5.

In addition, based on parameter experimental variability, we converted the model output-parameter dependence slope into a "parameter-dependent error" in terms of RCM-simulated tissue PO_2 ($sstPO_2$ slope vs parameter times 2 SD; SD reported in SM1). The most influential parameters (absolute slopes of 3.8–12.9 mmHg/parameter-unit) are: Na^+ reabsorption fraction (−12.9), oxygen solubility (+6.9), transcellular fraction of Na+ reabsorbed (−6.1) and PCT-bicarbonate factor (+3.8). Setting aside solubility, three of these directly influence epithelial oxygen consumption, while the last one relates to model morphometry (Nc/Nt, the number of capillaries per tubule).

Table S9 (below) reports the 12 most influent parameters (absolute value of associated error > 0.5 mmHg); signs are reminders of the parameter influence direction. For instance, experimental SD uncertainty about the transcellular Na^+ reabsorption fraction (0.81 ± 0.20, range 0.53–1.00; SM1-Table S4, Energetics and transport) translates into a tissue PO_2 error of 2.3 mmHg.

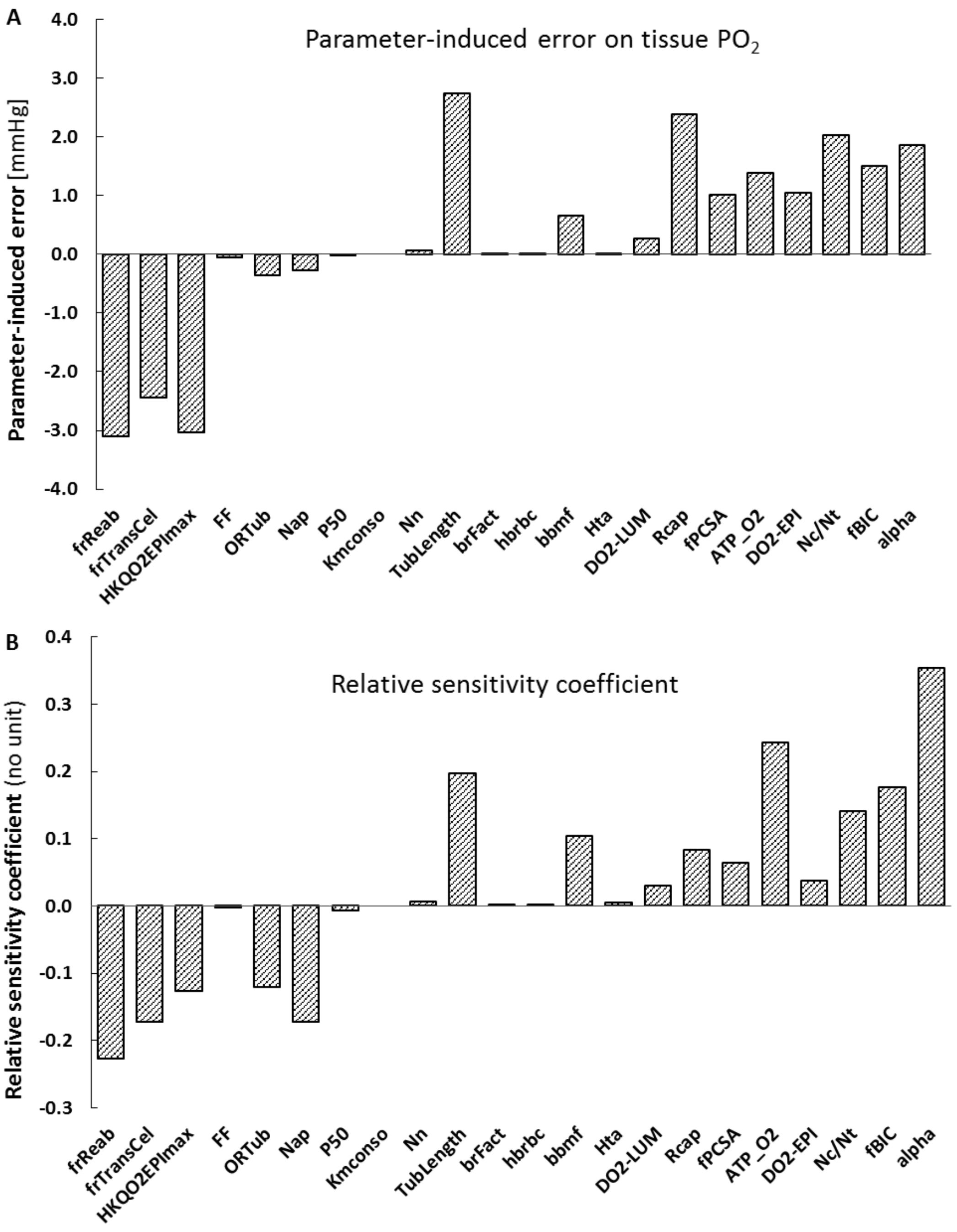

SMI-Figure S1. Parametric sensitivity analysis. The principal independent parameters (IP, $n = 22$) are tested for their relative and absolute influence upon steady-state tissue PO_2 ($sstPO_2$). (**a**) Parametric relative sensitivity coefficient (RSC) allows for direct comparison of IP's, on a normalized scale (see Methods). (**b**) The slope of $sstPO_2$ versus the parameter is multiplied by 2 × SD° of the parameter (see SM1 Tables S1–S8) in order to quantify the parameter-associated error in RCM. Conservatively, twice the (absolute) mean value of individual parametric errors is considered an estimate of RCM error (2.2 mmHg).

SM1-Table S9. Influence of main 22 parameters on tissue PO_2 output.

Parameter	Description	Parameter-dependent error (mmHg)
frReab	Fractional reabsorption	3.1 (−)
frTransC	Na Transcell. fraction	2.3 (−)
HKQO2EPImax	Max basal QO_2 in EPI	3.0 (−)

FF	Filtration fraction	0.1 (−)
OR-Tub	Tubular outer radius	0.3 (−)
Nap	Plasma sodium concentration	0.2 (−)
P50	Hemoglobin half-saturation PO_2	0.1 (−)
Km-conso	Apparent O_2 dissociation constant of transport-dependent consumption in EPI	0.0
Nn	Number of operational nephrons	0.1 (+)
Tub-length	Tubule length	2.9 (+)
Br-Fact	Capillary branching factor	0.0
Hb-RBC	Erythrocyte hemoglobin	0.0
bbmf	Brush-border membrane factor	0.7 (+)
Hta	Afferent hematocrit	0.0
DO2-LUM	Diffusion coefficient in LUM	0.4 (+)
Rcap	Capillary radius	2.5 (+)
fPCSA	Surface area factor for PTC	1.0 (+)
ATP-O2	Oxphos ATP/O_2 ratio	1.4 (+)
DO2-EPI	Diffusion coefficient in EPI	1.0 (+)
Nc/Nt	Capillary/Tubule ratio	1.9 (+)
fBIC	HCO_3-factor*	1.4 (+)
alpha	Tissue oxygen solubility	1.7 (+)

Unsorted values.

3.2. Input/output analysis (presented in main text)

3.3. Input-parameter interaction analysis

Because of the number of parameters and associated combinatorial, we limited the sensitivity analysis of parameter-inputs interactions to selected IP's. We explored the effect of four influential IP's, namely fractional reabsorption, fractional transcellular reabsorption, tubule length, and the ATP/O2 Oxphos ratio (all drivers of oxygen consumption), as they interact with RBF (5.3 and 0.2 FU) and PTC/LUM inputs (4 combinations).

In figure 2, panel A presents the control slope of the 4 parameters vs. tissue PO_2, at various PTC/LUM feeds, and at reference RBF (5.3 FU); panel B presents the same data, but obtained at low RBF (0.2 FU). Briefly, parameter control does not significantly change with oxygen inputs. At normal perfusion (RBF°, panel A), individual parametric influence is practically independent of the PTC/LUM feed combination. At low RBF (0.2 FU, panel B), the general parametric influence is markedly reduced, but remains independent of PTC/LUM feed, with the exception of control by fractional reabsorption which remains sizable (–8.1 mmHg), and becomes strongly dependent on oxygen feed, i.e. decreasing to –1.3 mmHg at 28/20 mmHg PTC/LUM feed.

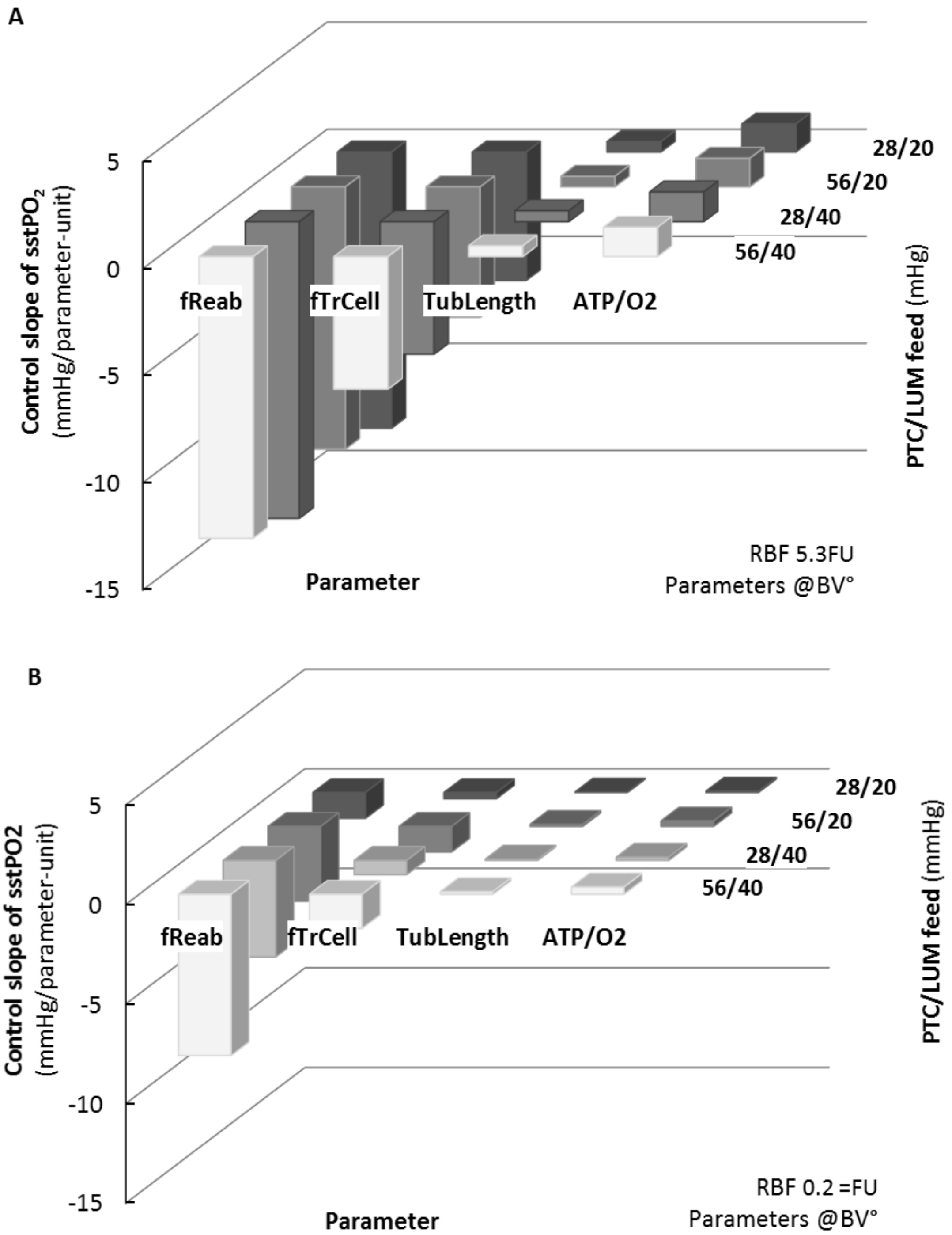

SM1-Figure S2. Input parameter interactions. Control of tissue PO_2 by oxygen feed and RBF, under the influence of selected parameters. Selected parameters were evaluated for their influence on the tissue PO_2 control by inputs, RBF and PTC/LUM oxygen feeds (RCM° parametric configuration was used for all other parameters). (**a**) The control slope of each parameter upon $sstPO_2$, at four different PTC/LUM feed combinations, under normal perfusion condition (RBF°, 5.3 FU). (**b**) Data obtained in exactly the same conditions, except for the lower perfusion level (RBF 0.2 FU). In terms of model accuracy, we infer from the above that changing PO_2 or RBF inputs will not increase model error, especially in experiments where ischemia (reduced RBF) or hypoxemia (reduced PO_2 input) will be simulated.

Reference

1. Johannes, T.; Mik, E.G.; Nohe, B.; Unertl, K.E.; Ince, C. Acute decrease in renal microvascular PO2 during acute normovolemic hemodilution. *Am J Physiol Renal Physiol* **2007**, *292*, F796–F803.
2. O'Connor, P.M.; Anderson, W.P.; Kett, M.M.; Evans, R.G. Renal preglomerular arterial-venous O2 shunting is a structural anti-oxidant defence mechanism of the renal cortex. *Clin Exp Pharmacol Physiol* **2006**, *33*, 637–641.
3. Welch, W.J.; Baumgartl, H.; Lubbers, D.; Wilcox, C.S. Nephron pO2 and renal oxygen usage in the hypertensive rat kidney. *Kidney Int* **2001**, *59*, 230–237.
4. Lindstedt, S.L.; Schaeffer, P.J. Use of allometry in predicting anatomical and physiological parameters of mammals. *Lab Anim* **2002**, *36*, 1–19.
5. Steinhausen, M.; Endlich, K.; Wiegman, D.L. Glomerular blood flow. *Kidney Int* **1990**, *38*, 769–784.
6. Thomas, S.R. Kidney modeling and systems physiology. *Wiley Interdiscip Rev Syst Biol Med* **2009**, *1*, 172–190.
7. Garcia-Sanz, A.; Rodriguez-Barbero, A.; Bentley, M.D.; Ritman, E.L.; Romero, J.C. Three-dimensional microcomputed tomography of renal vasculature in rats. *Hypertension* **1998**, *31*, 440–444.
8. Gardiner, B.S.; Thompson, S.L.; Ngo, J.P.; Smith, D.W.; Abdelkader, A.; Broughton, B.R.; Bertram, J.F.; Evans, R.G. Diffusive oxygen shunting between vessels in the preglomerular renal vasculature: Anatomic observations and computational modeling. *Am J Physiol Renal Physiol* **2012**, *303*, F605–F618.
9. Gardiner, B.S.; Smith, D.W.; O'Connor, P.M.; Evans, R.G. A mathematical model of diffusional shunting of oxygen from arteries to veins in the kidney. *Am J Physiol Renal Physiol* **2011**, *300*, F1339–F1352.
10. Lubbers, D.W.; Baumgartl, H. Heterogeneities and profiles of oxygen pressure in brain and kidney as examples of the pO2 distribution in the living tissue. *Kidney Int* **1997**, *51*, 372–380.
11. Steinhausen, M.; Eisenbach, G.M.; Bottcher, W. High-frequency microcinematographic measurements on peritubular blood flow under control conditions and after temporary ischemia of rat kidneys. *Pflugers Arch* **1973**, *339*, 273–288.
12. Dyson, A.; Bezemer, R.; Legrand, M.; Balestra, G.; Singer, M.; Ince, C. Microvascular and interstitial oxygen tension in the renal cortex and medulla studied in a 4-h rat model of LPS-induced endotoxemia. *Shock* **2011**, *36*, 83–89.
13. Johannes, T.; Mik, E.G.; Nohe, B.; Raat, N.J.; Unertl, K.E.; Ince, C. Influence of fluid resuscitation on renal microvascular PO2 in a normotensive rat model of endotoxemia. *Crit Care* **2006**, *10*, R88.
14. Wilcox, C.S.; Palm, F.; Welch, W.J. Renal oxygenation and function of the rat kidney: Effects of inspired oxygen and preglomerular oxygen shunting. *Adv Exp Med Biol* **2013**, *765*, 329–334.
15. Welch, W.J.; Baumgartl, H.; Lubbers, D.; Wilcox, C.S. Renal oxygenation defects in the spontaneously hypertensive rat: Role of AT1 receptors. *Kidney Int* **2003**, *63*, 202–208.
16. Welch, W.J.; Mendonca, M.; Aslam, S.; Wilcox, C.S. Roles of oxidative stress and AT1 receptors in renal hemodynamics and oxygenation in the postclipped 2K,1C kidney. *Hypertension* **2003**, *41*, 692–696.
17. Liss, P.; Nygren, A.; Revsbech, N.P.; Ulfendahl, H.R. Intrarenal oxygen tension measured by a modified clark electrode at normal and low blood pressure and after injection of x-ray contrast media. *Pflugers Arch* **1997**, *434*, 705–711.
18. Schurek, H.J.; Jost, U.; Baumgartl, H.; Bertram, H.; Heckmann, U. Evidence for a preglomerular oxygen diffusion shunt in rat renal cortex. *Am J Physiol* **1990**, *259*, F910–F915.
19. Gunther, H.; Aumuller, G.; Kunke, S.; Vaupel, P.; Thews, G. [The oxygen supply of the kidney. I. Distribution of O2 partial pressures in the rat kidney under normal conditions (author's transl)]. *Res Exp Med (Berl)* **1974**, *163*, 251–264.
20. Leichtweiss, H.P.; Lubbers, D.W.; Weiss, C.; Baumgartl, H.; Reschke, W. The oxygen supply of the rat kidney: Measurements of intrarenal pO2. *Pflugers Arch* **1969**, *309*, 328–349.
21. Dyson, A.; Bezemer, R.; Legrand, M.; Balestra, G.; Singer, M.; Ince, C. Microvascular and interstitial oxygen tension in the renal cortex and medulla studied in a 4-h rat model of LPS-induced endotoxemia. *Shock 36*, 83–89.
22. Whitehouse, T.; Stotz, M.; Taylor, V.; Stidwill, R.; Singer, M. Tissue oxygen and hemodynamics in renal medulla, cortex, and corticomedullary junction during hemorrhage-reperfusion. *Am J Physiol Renal Physiol* **2006**, *291*, F647–F653.

23. Grosenick, D.; Cantow, K.; Arakelyan, K.; Wabnitz, H.; Flemming, B.; Skalweit, A.; Ladwig, M.; Macdonald, R.; Niendorf, T.; Seeliger, E. Detailing renal hemodynamics and oxygenation in rats by a combined near-infrared spectroscopy and invasive probe approach. *Biomed Opt Express* **2015**, *6*, 309–323.
24. Black, M.J.; Briscoe, T.A.; Constantinou, M.; Kett, M.M.; Bertram, J.F. Is there an association between level of adult blood pressure and nephron number or renal filtration surface area? *Kidney Int* **2004**, *65*, 582–588.
25. Solomon, S. Developmental changes in nephron number, proximal tubular length and superficial nephron glomerular filtration rate of rats. *J Physiol* **1977**, *272*, 573–589.
26. de Rouffignac, C.; Monnens, L. Functional and morphologic maturation of superficial and juxtamedullary nephrons in the rat. *J Physiol* **1976**, *262*, 119–129.
27. Knepper MA, Danielson RA, Saidel GM, P.RS. Quantitative analysis of renal medullary anatomy in rats and rabbits. *Kidney Int* **1977**, *12*, 313–323.
28. Badzynska, B.; Sadowski, J. Opposed effects of prostaglandin E2 on perfusion of rat renal cortex and medulla: Interactions with the renin-angiotensin system. *Exp Physiol* **2008**, *93*, 1292–1302.
29. Roald, A.B.; Ofstad, J.; Iversen, B.M. Attenuated buffering of renal perfusion pressure variation in juxtamedullary cortex in SHR. *Am J Physiol Renal Physiol* **2002**, *282*, F506–F511.
30. Roman, R.J.; Cowley Jr., A.W.; Garcia-Estan, J.; Lombard, J.H. Pressure-diuresis in volume-expanded rats. Cortical and medullary hemodynamics. *Hypertension* **1988**, *12*, 168–176.
31. Khan, M.A.H.; Islam, M.T.; Castillo, A.; Majid, D.S.A. Attenuation of renal excretory responses to ANG II during inhibition of superoxide dismutase in anesthetized rats. *American journal of physiology. Renal physiology* **2010**, *298*, F401–F407.
32. Mertz, J.I.; Haas, J.A.; Berndt, T.J.; Burnett Jr., J.C.; Knox, F.G. Effects of secretin on peritubular capillary physical factors and proximal fluid reabsorption in the rat. *J Clin Invest* **1983**, *72*, 622–625.
33. Jackson, B.; Oken, D.E. Internephron heterogeneity of filtration fraction and disparity between protein- and hematocrit-derived values. *Kidney Int* **1982**, *21*, 309–315.
34. Yarger, W.E.; Boyd, M.A.; Schrader, N.W. Evaluation of methods of measuring glomerular and nutrient blood flow in rat kidneys. *Am J Physiol* **1978**, *235*, H592–H600.
35. Myers, B.D.; Deen, W.M.; Robertson, C.R.; Brenner, B.M. Dynamics of glomerular ultrafiltration in the rat. VIII. Effects of hematocrit. *Circ Res* **1975**, *36*, 425–435.
36. Kaufman, J.M.; Siegel, N.J.; Hayslett, J.P. Functional and hemodynamic adaptation to progressive renal ablation. *Circ Res* **1975**, *36*, 286–293.
37. Kallskog, O.; Lindbom, L.O.; Ulfendahl, H.R.; Wolgast, M. Kinetics of the glomerular ultrafiltration in the rat kidney. An experimental study. *Acta Physiol Scand* **1975**, *95*, 293–300.
38. Ichikawa, I.; Hoyer, J.R.; Seiler, M.W.; Brenner, B.M. Mechanism of glomerulotubular balance in the setting of heterogeneous glomerular injury. *J Clin Invest* **1982**, *69*, 185–198.
39. Christensen, E.I.; Grann, B.; Kristoffersen, I.B.; Skriver, E.; Thomsen, J.S.; Andreasen, A. Three-dimensional reconstruction of the rat nephron. *Am J Physiol Renal Physiol* **2014**, *306*, F664–F671.
40. de Rouffignac, C.; Morel, F. [Misrodissection study of the distribution and length of the proximal tubules of the kidney of five species of rodents]. *Arch Anat Microsc Morphol Exp* **1967**, *56*, 123–132.
41. Kriz, W. [The architectonic and functional structure of the rat kidney]. *Z Zellforsch Mikrosk Anat* **1967**, *82*, 495–535.
42. Moss, R.; Thomas, S.R. Hormonal regulation of salt and water excretion: A mathematical model of whole kidney function and pressure natriuresis. *Am J Physiol Renal Physiol* **2014**, *306*, F224–F248.
43. Lopez, B.; Moreno, C.; Salom, M.G.; Roman, R.J.; Fenoy, F.J. Role of guanylyl cyclase and cytochrome P-450 on renal response to nitric oxide. *Am J Physiol Renal Physiol* **2001**, *281*, F420–F427.
44. de Vries, P.A.; Navis, G.; de Jong, P.E.; de Zeeuw, D. Can continuous intraperitoneal infusion of 125I-iothalamate and 131I-hippuran be used for measurement of GFR in conscious rats? *Ren Fail* **1998**, *20*, 249–255.
45. Jobin, J.; Bonjour, J.P. Measurement of glomerular filtration rate in conscious unrestrained rats with inulin infused by implanted osmotic pumps. *Am J Physiol* **1985**, *248*, F734–F738.
46. Brodsky, S.; Gurbanov, K.; Abassi, Z.; Hoffman, A.; Ruffolo, R.R.J.; Feuerstein, G.Z.; Winaver, J. Effects of eprosartan on renal function and cardiac hypertrophy in rats with experimental heart failure. *Hypertension* **1998**, *32*, 746–752.
47. Tanner, G.A.; Knopp, L.C. Glomerular blood flow after single nephron obstruction in the rat kidney. *Am J Physiol* **1986**, *250*, F77–F85.

48. Robertson, C.R.; Deen, W.M.; Troy, J.L.; Brenner, B.M. Dynamics of glomerular ultrafiltration in the rat. 3. Hemodynamics and autoregulation. *Am J Physiol* **1972**, *223*, 1191–1200.
49. Brenner, B.M.; Troy, J.L.; Daugharty, T.M.; Deen, W.M.; Robertson, C.R. Dynamics of glomerular ultrafiltration in the rat. II. Plasma-flow dependence of GFR. *Am J Physiol* **1972**, *223*, 1184–1190.
50. Kon, V.; Hughes, M.L.; Ichikawa, I. Blood flow dependence of postglomerular fluid transfer and glomerulotubular balance. *J Clin Invest* **1983**, *72*, 1716–1728.
51. Blantz, R.C.; Gabbai, F.B.; Peterson, O.; Wilson, C.B.; Kihara, I.; Kawachi, H.; Shimizu, F.; Yamamoto, T. Water and protein permeability is regulated by the glomerular epithelial slit diaphragm. *J Am Soc Nephrol* **1994**, *4*, 1957–1964.
52. Wright, F.S.; Giebisch, G. Glomerular filtration in single nephrons. *Kidney Int* **1972**, *1*, 201–209.
53. Romano, G.; Favret, G.; Damato, R.; Bartoli, E. Proximal reabsorption with changing tubular fluid inflow in rat nephrons. *Exp Physiol* **1998**, *83*, 35–48.
54. Preisig, P.A.; Ives, H.E.; Cragoe Jr., E.J.; Alpern, R.J.; Rector Jr., F.C. Role of the Na+/H+ antiporter in rat proximal tubule bicarbonate absorption. *J Clin Invest* **1987**, *80*, 970–978.
55. Panico, C.; Luo, Z.; Damiano, S.; Artigiano, F.; Gill, P.; Welch, W.J. Renal proximal tubular reabsorption is reduced in adult spontaneously hypertensive rats: Roles of superoxide and Na+/H+ exchanger 3. *Hypertension* **2009**, *54*, 1291–1297.
56. Jensen, P.K. Continuous measurement of flow rate and volume in the nanoliter range. *Acta Physiol Scand* **1979**, *106*, 5–9.
57. Baines, A.D.; de Rouffignac, C. Functional heterogeneity of nephrons. II. Filtration rates, intraluminal flow velocities and fractional water reabsorption. *Pflugers Arch* **1969**, *308*, 260–276.
58. Baines, A.D.; Baines, C.J.; de Rouffignac, C. Functional heterogeneity of nephrons. I. Intraluminal flow velocities. *Pflugers Arch* **1969**, *308*, 244–259.
59. Bonvalet, J.P.; de Rouffignac, C. Distribution of ferrocyanide along the proximal tubular lumen of the rat kidney: Its implications upon hydrodynamics. *J Physiol* **1981**, *318*, 85–98.
60. Gertz, K.H.; Mangos, J.A.; Braun, G.; Pagel, H.D. On the glomerular tubular balance in the rat kidney. *Pflugers Arch Gesamte Physiol Menschen Tiere* **1965**, *285*, 360–372.
61. Matsumoto, M.; Tanaka, T.; Yamamoto, T.; Noiri, E.; Miyata, T.; Inagi, R.; Fujita, T.; Nangaku, M. Hypoperfusion of peritubular capillaries induces chronic hypoxia before progression of tubulointerstitial injury in a progressive model of rat glomerulonephritis. *J Am Soc Nephrol* **2004**, *15*, 1574–1581.
62. Yamamoto, T.; Tada, T.; Brodsky, S. V.; Tanaka, H.; Noiri, E.; Kajiya, F.; Goligorsky, M.S. Intravital videomicroscopy of peritubular capillaries in renal ischemia. *Am J Physiol Renal Physiol* **2002**, *282*, F1150–F1155.
63. Simsek, N.; Altunkaynak, B.Z.; Unal, D.; Can, S.; Malkoc, I.; Unal, B. A stereological and electron microscopic study of the development of the nephron in prenatal and postnatal rats. *Eurasian J Med* **2009**, *41*, 84–90.
64. Ijpelaar, D.H.; Schulz, A.; Koop, K.; Schlesener, M.; Bruijn, J.A.; Kerjaschki, D.; Kreutz, R.; de Heer, E. Glomerular hypertrophy precedes albuminuria and segmental loss of podoplanin in podocytes in Munich-Wistar-Fromter rats. *Am J Physiol Renal Physiol* **2008**, *294*, F758–F767.
65. Ellison, D.H.; Velazquez, H.; Wright, F.S. Adaptation of the distal convoluted tubule of the rat. Structural and functional effects of dietary salt intake and chronic diuretic infusion. *J Clin Invest* **1989**, *83*, 113–126.
66. Ichikawa, I.; Schor, N.; Brenner, B.M. Effects of parathyroid hormone on glomerular ultrafiltration in the rat. *Adv Exp Med Biol* **1982**, *151*, 577–585.
67. Niederalt, C.; Wendl, T.; Kuepfer, L.; Claassen, K.; Loosen, R.; Willmann, S.; Lippert, J.; Schultze-Mosgau, M.; Winkler, J.; Burghaus, R.; et al. Development of a physiologically based computational kidney model to describe the renal excretion of hydrophilic agents in rats. *Front Physiol* **2013**, *3*, 494.
68. Rasch, R.; Dorup, J. Quantitative morphology of the rat kidney during diabetes mellitus and insulin treatment. *Diabetologia* **1997**, *40*, 802–809.
69. Pfaller, W.; Rittinger, M. Quantitative morphology of the rat kidney. *Int J Biochem* **1980**, *12*, 17–22.
70. Ortiz, M.C.; Garcia-Sanz, A.; Bentley, M.D.; Fortepiani, L.A.; Garcia-Estan, J.; Ritman, E.L.; Romero, J.C.; Juncos, L.A. Microcomputed tomography of kidneys following chronic bile duct ligation. *Kidney Int* **2000**, *58*, 1632–1640.

71. Leh, S.; Hultstrom, M.; Rosenberger, C.; Iversen, B.M. Afferent arteriolopathy and glomerular collapse but not segmental sclerosis induce tubular atrophy in old spontaneously hypertensive rats. *Virchows Arch* **2011**, *459*, 99–108.
72. Faarup, P.; Saelan, H.; Ryo, G. Correlation between tubules and capillaries and size of interstitial space in the functioning rat kidney. Influence of different types of preparation. *Acta Pathol Microbiol Scand A* **1971**, *79*, 607–616.
73. Dorup, J.; Maunsbach, A.B. Three-dimensional organization and segmental ultrastructure of rat proximal tubules. *Exp Nephrol* **1997**, *5*, 305–317.
74. Golalipour, M.J.; Azarhoush, R.; Ghafari, S.; Davarian, A.; Fazeli, S.A. Can Formaldehyde Exposure Induce Histopathologic and Morphometric Changes on Rat Kidney? *International Journal of Morphology* **2009**, *27*.
75. Picard, N.; Baum, O.; Vogetseder, A.; Kaissling, B.; Le Hir, M. Origin of renal myofibroblasts in the model of unilateral ureter obstruction in the rat. *Histochemistry and cell biology* **2008**, *130*, 141–155.
76. Bentley, M.D.; Ortiz, M.C.; Ritman, E.L.; Romero, J.C. The use of microcomputed tomography to study microvasculature in small rodents. *Am J Physiol Regul Integr Comp Physiol* **2002**, *282*, R1267–R1279.
77. Jensen, P.K.; Steven, K. Angiotensin II induced reduction of peritubular capillary diameter in the rat kidney. *Pflugers Arch* **1977**, *371*, 245–250.
78. Ohashi, R.; Shimizu, A.; Masuda, Y.; Kitamura, H.; Ishizaki, M.; Sugisaki, Y.; Yamanaka, N. Peritubular capillary regression during the progression of experimental obstructive nephropathy. *J Am Soc Nephrol* **2002**, *13*, 1795–1805.
79. Ohashi, R.; Kitamura, H.; Yamanaka, N. Peritubular capillary injury during the progression of experimental glomerulonephritis in rats. *J Am Soc Nephrol* **2000**, *11*, 47–56.
80. de Rouffignac, C.; Bonvalet, J.P. [Study of variations in the glomerular filtration rate of single superficial and deep nephrons as a function of sodium intake in the rat]. *Pflugers Arch* **1970**, *317*, 141–156.
81. de Rouffignac, C.; Deiss, S.; Bonvalet, J.P. [Determination of the glomerular filtration rate of individual nephrons accessible and inaccessible to micropuncture]. *Pflugers Arch* **1970**, *315*, 273–290.
82. Seiller, W.; Gertz, K. Single Nephron Filtration, Luminal Flow and Tubular Fluid Reabsorption along the Proximal Convolution and the Pars recta of the Rat Kidney as Influenced by Luminal Pressure Changes. *Pflügers Archiv - European Journal of Physiology* **1977**, *371*, 235–243.
83. Chang, H.; Fujita, T. A numerical model of the renal distal tubule. *Am J Physiol* **1999**, *276*, F931–F951.
84. Manotham, K.; Tanaka, T.; Matsumoto, M.; Ohse, T.; Miyata, T.; Inagi, R.; Kurokawa, K.; Fujita, T.; Nangaku, M. Evidence of tubular hypoxia in the early phase in the remnant kidney model. *J Am Soc Nephrol* **2004**, *15*, 1277–1288.
85. Barbuto, N.; Almeida, J.R.; Pereira, L.M.; Mandarim-de-Lacerda, C.A. Renal cortex remodeling in nitric oxide deficient rats treated with enalapril. *J Cell Mol Med* **2004**, *8*, 102–108.
86. Pereira, L.M.M.; Almeida, J.R.; Mandarim-de-Lacerda, C.A. Kidney adaptation in nitric oxide-deficient Wistar and spontaneously hypertensive rats. *Life Sciences* **2004**, *74*, 1375–1386.
87. Mik, E.G.; Johannes, T.; Ince, C. Monitoring of renal venous PO2 and kidney oxygen consumption in rats by a near-infrared phosphorescence lifetime technique. *Am J Physiol Renal Physiol* **2008**, *294*, F676–F681.
88. Parekh, N.; Veith, U. Renal hemodynamics and oxygen consumption during postischemic acute renal failure in the rat. *Kidney Int* **1981**, *19*, 306–316.
89. Elinder, G.; Aperia, A. Renal oxygen consumption and sodium reabsorption during isotonic volume expansion in the developing rat. *Pediatr Res* **1982**, *16*, 351–353.
90. Deen, W.M.; Robertson, C.R.; Brenner, B.M. A model of peritubular capillary control of isotonic fluid reabsorption by the renal proximal tubule. *Biophys J* **1973**, *13*, 340–358.
91. Peterson, O.W.; Gushwa, L.C.; Blantz, R.C. An analysis of glomerular-tubular balance in the rat proximal tubule. *Pflügers Archiv - European Journal of Physiology* **1986**, *407*, 221–227.
92. Thomson, S.; Blantz, R.C. Homeostatic efficiency of tubuloglomerular feedback in hydropenia, euvolemia, and acute volume expansion. *Am J Physiol Renal Physiol* **1993**, *33*, F930–F936.
93. Tucker, B.J.; Blantz, R.C. Determinants of proximal tubular reabsorption as mechanisms of glomerulotubular balance. *Am J Physiol* **1978**, *235*, F142–F150.
94. Schnermann, J.; Wahl, M.; Liebau, G.; Fischbach, H. Balance between Tubular Flow Rate and Net Fluid Reabsorption in the Proximal Convolution of the Rat Kidney*. *Pflügers Archiv - European Journal of Physiology* **1968**, *304*, 90–103.

95. Zhang, W.; Edwards, A. Oxygen transport across vasa recta in the renal medulla. *Am J Physiol Heart Circ Physiol* **2002**, *283*, H1042–H1055.
96. Reilly, R.F.; Ellison, D.H. Mammalian distal tubule: Physiology, pathophysiology, and molecular anatomy. *Physiol Rev* **2000**, *80*, 277–313.
97. Giebisch, G.; Klose, R.M.; Malnic, G.; Sullivan, W.J.; Windhager, E.E. Sodium Movement across Single Perfused Proximal Tubules of Rat Kidneys. *J Gen Physiol* **1964**, *47*, 1175–1194.
98. Larsen, E.H.; Mobjerg, N.; Sorensen, J.N. Fluid transport and ion fluxes in mammalian kidney proximal tubule: A model analysis of isotonic transport. *Acta Physiol (Oxf)* **2006**, *187*, 177–189.
99. Weinstein, A.M.; Weinbaum, S.; Duan, Y.; Du, Z.; Yan, Q.; Wang, T. Flow-dependent transport in a mathematical model of rat proximal tubule. *Am J Physiol Renal Physiol* **2007**, *292*, F1164–F1181.
100. Weinstein, A.M. Osmotic diuresis in a mathematical model of the rat proximal tubule. *Am J Physiol* **1986**, *250*, F874–F884.
101. Weinstein, A.M. A mathematical model of the rat proximal tubule. *Am J Physiol* **1986**, *250*, F860–F873.
102. Brand, M. The efficiency and plasticity of mitochondrial energy transduction. *Biochemical Society Transactions* **2005**, *33*, 897–904.
103. Freeman, D.; Bartlett, S.; Radda, G.; Ross, B. Energetics of sodium transport in the kidney. Saturation transfer 31P-NMR. *Biochim Biophys Acta* **1983**, *762*, 325–336.
104. Mergner, W.J.; Smith, M.W.; Trump, B.F. Studies on the pathogenesis of ischemic cell injury. XI. P/O ratio and acceptor control. *Virchows Arch B Cell Pathol* **1977**, *26*, 17–26.
105. Scandurra, F.M.; Gnaiger, E. Cell respiration under hypoxia: Facts and artefacts in mitochondrial oxygen kinetics. *Adv Exp Med Biol* **2010**, *662*, 7–25.
106. Gnaiger, E. Bioenergetics at low oxygen: Dependence of respiration and phosphorylation on oxygen and adenosine diphosphate supply. *Respir Physiol* **2001**, *128*, 277–297.
107. Peterson, O.W.; Gushwa, L.C.; Blantz, R.C. An analysis of glomerular-tubular balance in the rat proximal tubule. *Pflugers Arch* **1986**, *407*, 221–227.
108. Schnermann, J.; Wahl, M.; Liebau, G.; Fischbach, H. Balance between tubular flow rate and net fluid reabsorption in the proximal convolution of the rat kidney. I. Dependency of reabsorptive net fluid flux upon proximal tubular surface area at spontaneous variations of filtration rate. *Pflugers Arch* **1968**, *304*, 90–103.
109. Gullans, S.R.; Mandel, L. COUPLING OF ENERGY TO TRANSPORT IN PROXIMAL AND DISTAL NEPHRON. In *The kidney: Physiology and pathophysiology*; Seldin, D.W., Giebisch, G., Eds.; Lippincott Williams and Wilkins, 2000; Vol. 1, pp. 445–482.
110. Garg, L.C.; Mackie, S.; Tisher, C.C. Effect of low potassium-diet on Na-K-ATPase in rat nephron segments. *Pflugers Arch* **1982**, *394*, 113–117.
111. Katz, A.I.; Doucet, A.; Morel, F. Na-K-ATPase activity along the rabbit, rat, and mouse nephron. *Am J Physiol* **1979**, *237*, F114–F120.
112. Soltoff, S.P. ATP and the Regulation of Renal Cell Function. *Ann Rev Physiol* **1986**, *48*, 9–31.
113. Guder, W.G.; Ross, B.D. Enzyme distribution along the nephron. *Kidney International* **1984**, *26*, 101–111.
114. Evans, R.G.; Harrop, G.K.; Ngo, J.P.; Ow, C.P.; O'Connor, P.M. Basal renal O2 consumption and the efficiency of O2 utilization for Na+ reabsorption. *Am J Physiol Renal Physiol* **2014**, *306*, F551–F560.
115. Pittman, R.N. Oxygen transport in the microcirculation and its regulation. *Microcirculation* **2013**, *20*, 117–137.
116. Sasaki, N.; Horinouchi, H.; Ushiyama, A.; Minamitani, H. A new method for measuring the oxygen diffusion constant and oxygen consumption rate of arteriolar walls. *Keio J Med* **2012**, *61*, 57–65.
117. Vadapalli, A.; Pittman, R.N.; Popel, A.S. Estimating oxygen transport resistance of the microvascular wall. *Am J Physiol Heart Circ Physiol* **2000**, *279*, H657–H671.
118. Papandreou, I.; Cairns, R.A.; Fontana, L.; Lim, A.L.; Denko, N.C. HIF-1 mediates adaptation to hypoxia by actively downregulating mitochondrial oxygen consumption. *Cell Metabolism* **2006**, *3*, 187–197.
119. Fukuda, S.; Tsuchikura, S.; Iida, H. Age-related changes in blood pressure, hematological values, concentrations of serum biochemical constituents and weights of organs in the SHR/Izm, SHRSP/Izm and WKY/Izm. *Exp Anim* **2004**, *53*, 67–72.
120. Boonjarern, S.; Laski, M.E.; Kurtzman, N.A. Effects of extracellular volume expansion on the tubular reabsorption of glucose. A microinjection study. *Pflugers Arch* **1976**, *366*, 67–71.

121. Baines, A.D. Effect of extracellular fluid volume expansion on maximum glucose reabsorption rate and glomerular tubular balance in single rat nephrons. *J Clin Invest* **1971**, *50*, 2414–2425.
122. Stein, S.; Cherian, M.G.; Mazur, A. Preparation and properties of six rat hemoglobins. Nonuniform biosynthesis in marrow erythroid cells. *J Biol Chem* **1971**, *246*, 5287–5293.
123. Sharp, P.; Villano, J. *The Laboratory Rat*; Press, C.R.C., Group, T.& F., Eds.; Boca Raton, FL, 2012; ISBN 978-1-4398-2986-8.
124. Schmidt, W.; Correa, R.; Boning, D.; Ehrich, J.H.; Kruger, C. Oxygen transport properties in malaria-infected rodents--a comparison between infected and noninfected erythrocytes. *Blood* **1994**, *83*, 3746–3752.
125. Cartheuser, C.F. Standard and pH-affected hemoglobin-O2 binding curves of Sprague-Dawley rats under normal and shifted P50 conditions. *Comp Biochem Physiol Comp Physiol* **1993**, *106*, 775–782.
126. Mairbaurl, H.; Humpeler, E. Diminution of the temperature effects on the oxygen affinity of hemoglobin after prolonged hypothermia. *Pflugers Arch* **1980**, *383*, 209–213.
127. Clark Jr., A.; Federspiel, W.J.; Clark, P.A.; Cokelet, G.R. Oxygen delivery from red cells. *Biophys J* **1985**, *47*, 171–181.
128. Wittenberg, J.B. The molecular mechanism of hemoglobin-facilitated oxygen diffusion. *J Biol Chem* **1966**, *241*, 104–114.
129. Bouwer, S.T.; Hoofd, L.; Kreuzer, F. Reaction rates of oxygen with hemoglobin measured by non-equilibrium facilitated oxygen di¡usion through hemoglobin solutions. *Biochimica et Biophysica Acta* **2000**, 10.
130. Graf, C.; Maser-Gluth, C.; de Muinck Keizer, W.; Rettig, R. Sodium retention and hypertension after kidney transplantation in rats. *Hypertension* **1993**, *21*, 724–730.
131. Adler, S.; Huang, H. Impaired regulation of renal oxygen consumption in spontaneously hypertensive rats. *J Am Soc Nephrol* **2002**, *13*, 1788–1794.
132. Brown, D.A.; MacLellan, W.R.; Laks, H.; Dunn, J.C.; Wu, B.M.; Beygui, R.E. Analysis of oxygen transport in a diffusion-limited model of engineered heart tissue. *Biotechnol Bioeng* **2007**, *97*, 962–975.
133. Grote, J.; Susskind, R.; Vaupel, P. Oxygen diffusivity in tumor tissue (DS-carcinosarcoma) under temperature conditions within the range of 20--40 degrees C. *Pflugers Arch* **1977**, *372*, 37–42.
134. Kreuzer, F. Gas diffusion in the tissues. *Folia Med Neerl* **1970**, *13*, 108–112.
135. Spaeth, E.E.; Friedlander, S.K. The diffusion of oxygen, carbon dioxide, and inert gas in flowing blood. *Biophys J* **1967**, *7*, 827–851.
136. Wise, D.L.; Houghton, G. The diffusion coefficients of ten slightly soluble gases in water at 10â€"60 C. *Chemical Engineering Science* **1966**, *21*, 999–1010.
137. Moschandreou, T.E.; Ellis, C.G.; Goldman, D. Influence of tissue metabolism and capillary oxygen supply on arteriolar oxygen transport: A computational model. *Mathematical Biosciences* **2011**, *232*, 1–10.
138. Clark, A.R.; Stokes, Y.M.; Lane, M.; Thompson, J.G. Mathematical modelling of oxygen concentration in bovine and murine cumulus-oocyte complexes. *Reproduction* **2006**, *131*, 999–1006.
139. Vadapalli, A.; Goldman, D.; Popel, A.S. Calculations of oxygen transport by red blood cells and hemoglobin solutions in capillaries. *Artif Cells Blood Substit Immobil Biotechnol* **2002**, *30*, 157–188.
140. Goldstick, T.K.; Ciuryla, V.T.; Zuckerman, L. Diffusion of oxygen in plasma and blood. *Adv Exp Med Biol* **1976**, *75*, 183–190.
141. Fischkoff, S.; Vanderkooi, J.M. Oxygen diffusion in biological and artificial membranes determined by the fluorochrome pyrene. *J Gen Physiol* **1975**, *65*, 663–676.
142. Spaan, J.A.; Kreuzer, F.; van Wely, F.K. Diffusion coefficients of oxygen and hemoglobin as obtained simultaneously from photometric determination of the oxygenation of layers of hemoglobin solutions. *Pflugers Arch* **1980**, *384*, 241–251.
143. Sharan, M.; Selvakumar, S. The effects of chemical kinetics on oxygen delivery to tissue. *Math Biosci* **1992**, *108*, 253–277.
144. Bentley, T.B.; Meng, H.; Pittman, R.N. Temperature dependence of oxygen diffusion and consumption in mammalian striated muscle. *Am J Physiol* **1993**, *264*, H1825–H1830.
145. Dutta, A.; Popel, A.S. A theoretical analysis of intracellular oxygen diffusion. *J Theor Biol* **1995**, *176*, 433–445.
146. Chow, D.C.; Wenning, L. a; Miller, W.M.; Papoutsakis, E.T. Modeling pO(2) distributions in the bone marrow hematopoietic compartment. II. Modified Kroghian models. *Biophysical journal* **2001**, *81*, 685–696.

147. Bouwer, S.T.; Hoofd, L.; Kreuzer, F. Diffusion coefficients of oxygen and hemoglobin measured by facilitated oxygen diffusion through hemoglobin solutions. *Biochim Biophys Acta* **1997**, *1338*, 127–136.
148. Liu, C.; Eskin, S.; Hellums, J. The oxygen permeability of cultured endothelial cell monolayers. *Adv Exp Med Biol* **1994**, *345*, 723–730.
149. Rumsey, W.L.; Schlosser, C.; Nuutinen, E.M.; Robiolio, M.; Wilson, D.F. Cellular energetics and the oxygen dependence of respiration in cardiac myocytes isolated from adult rat. *J Biol Chem* **1990**, *265*, 15392–15402.
150. Gnaiger, E.; Lassnig, B.; Kuznetsov, A.; Rieger, G.; Margreiter, R. Mitochondrial oxygen affinity, respiratory flux control and excess capacity of cytochrome c oxidase. *J Exp Biol* **1998**, *201*, 1129–1139.
151. Gutierrez, G. The rate of oxygen release and its effect on capillary O2 tension: A mathematical analysis. *Respir Physiol* **1986**, *63*, 79–96.
152. Groebe, K. An easy-to-use model for O2 supply to red muscle. Validity of assumptions, sensitivity to errors in data. *Biophys J* **1995**, *68*, 1246–1269.
153. Groebe, K. A versatile model of steady state 02 supply to tissue Application to skeletal muscle. *Biophysical Journal* **1990**, *57*, 485–498.
154. Tsai, A.G.; Johnson, P.C.; Intaglietta, M. Oxygen gradients in the microcirculation. *Physiol Rev* **2003**, *83*, 933–963.
155. Tsai, A.G.; Friesenecker, B.; Mazzoni, M.C.; Kerger, H.; Buerk, D.G.; Johnson, P.C.; Intaglietta, M. Microvascular and tissue oxygen gradients in the rat mesentery. *Proc Natl Acad Sci U S A* **1998**, *95*, 6590–6595.
156. Dutta, A.; Wang, L.; Meng, H.; Pittman, R.N.; Popel, A.S. Oxygen tension profiles in isolated hamster retractor muscle at different temperatures. *Microvasc Res* **1996**, *51*, 288–302.
157. Zander, R. [Experimental Research on the O2 supply of the eye author's transl]. *Albrecht Von Graefes Arch Klin Exp Ophthalmol* **1975**, *195*, 215–230.
