# Supplementary material for: A Computer Model of Oxygen Dynamics in the Cortex of the Rat Kidney at the Cell-Tissue Level
Source: Int J Mol Sci. 2019 Dec 11;20(24):6246. doi: 10.3390/ijms20246246 (PMC6941061; doi:10.3390/ijms20246246)
Supplement: Supplementary file 1 [file ijms-20-06246-s001.zip › ijms-596297-SI-to conversion/ijms-596297-Supplementary Materials File 2-SM2-to conversion.pdf]

International Journal of
*Molecular Sciences*

# Supplemental Materials File 2—SM2

**Section 1. RCM tissue geometry, verification and variants**
**Section 2. Partitioning of renal oxygen consumption**
**Section 3. Diffusion routine**
**Section 4. Hemoglobin/$O_2$ dynamics**
**Section 5. Sensitivity analysis and I/O analysis**
**Section 6. References**

## 1. RCM tissue geometry, verification and tissue variants

### *1.1. Verification of model morphometry*

We constructed the base-case RCM tissue with the following reference morphometric characteristics (tissue "1540", for ~1.5 capillary per patch and 40µm outer tubular diameter):

(i) 55 tubular sections in ~1/10th of mm$^2$ (vs. 50 ± 8, *n* = 6), of luminal surface area 300–500 µm$^2$ (average 389 ± 25 µm$^2$ vs. 442 ± 138 µm$^2$, n = 14) [43,47];

(ii) ~20 µm outer radius and ~10 µm inner radius (vs. orPT° = 21.5 µm and irPT° = 11.4° µm, respectively); cell height 10 µm (vs. CH° = 9.9 µm);

(iii) 1.4 capillary section per tubule section (Nc/Nt° = 1.6) i.e. 79 capillary sections (rCap set at 5.0 µm); this is equivalent to 790 capillaries/mm$^2$, which compares to average experimental value, CapD° = 887 mm$^{-2}$ (SM1-Table S3a).

In addition, tissue "1540" exhibits the following fractional volumes: capillaries 7%-*v*/*v*, epithelial cells 66%-*v*/*v*, luminal area 22%-*v*/*v* (total tubular area = 88%-*v*/*v*), and interstitial areas 5%-*v*/*v*. Tubular length and surface density amount to 350 m/gkw (vs. 447) and 60 (vs. 48) mm$^2$/mm$^3$, respectively. Capillary surface area in RCM amounts to 402 cm$^2$/g-cortex (slightly higher than 300 cm$^2$/g-cortex reported in [10]). Table SM2-Table S1 compares these six morphometric indicators of tissue "1540" with "real" Rat cortex: the average mean tissue-to-source ratio is 0.99 ± 0.28. Thus, on morphometric grounds, tissue "1540" lies close to the average, Rat renal cortex.

**SM2-Table S1.** Verification of RCM morphometry.

| | **Tissue 1540** | **Source** | **Ratio** |
|---|---|---|---|
| Tubules (EPI+LUM patches) | 88.0 %vv | 76.0 ± 7.0 %vv | 1.16 |
| Interstitium (INT patches) | 5.0 %vv | 8.0 ± 4.0 %vv | 0.63 |
| Tubular length density | 350 m/gkw | 447 ± 45 m/gkw | 0.78 |
| Tubular surface density | 60 mm2/mm3 | 48 mm2/mm3 * | 1.25 |
| Capillaries (PTC patches) | 7.0 %vv | 9.0 ± 3.0 %vv | 0.78 |
| Capillary surface area (PCSA) | 402 cm2/gkw* | 300 ± 38 cm2/gkw** | 1.33 |
| | | **M ± SD** | **0.99 ± 0.28** |

"Source" refers to BV° from bibliographical analysis as reported in Supplemental material (SM1-Table S2), except for: *, n=1 [48], and **, at Rc = 5.0 µm; source value from [10].

### *1.2. Tissue variants*

In order to cover the experimental ranges of the two main geometrical independent parameters, outer tubule radius (orTub) and the number of capillary section per tubule section (Nc/Nt, see SM1-Table S3a), we devised six different tissue models, "scanning" both parameters range (orTub = 40 and 50 µm, and Nc/Nt =1.0, 1.5 and 2.0), including the reference tissue "1540". Geometry and patch composition of these model tissues is reported in Table S2, below. Of note, the real cortex exhibits 76%v/v of tubules (see SM1-Table S3a, Morphometry), whereas in the "1540" tissue it is 88%, yielding

a model/real cortex ratio of 1.16: thus, the over-estimating factor 1.27 = 4/π (due to square patches) is half-negated in our construct.

**SM2-Table S2.** Tissue variants and model geometry (tubule diameter and capillary density).

| | Model | | Patches |
|---|---|---|---|
| Dimensions | 320 µ × 320 µ<br>thickness 10 µ | Volume<br>1.024 nL | *n* = 1,024<br>(900) [1] |
| **Model tissues with 40 µ-diameter tubules** | | | |
| Tissue id | "1040" | "**1540**" | "2040" |
| Cap/tub ratio | 1.0 | **1.5** | 2.0 |
| Tub. ∅ [µ] | 40 | **40** | 40 |
| Cap. ∅ [µ] | 10 | **10** | 10 |
| Number of patches *per* type* (%*v*/*v*) [2] | | | |
| Vascular patches | 42 (5%) | **61 (7%)** | 83 (9%) |
| Epithelial patches | 600 (67%) | **598 (66%)** | 582 (65%) |
| Luminal patches | 200 (22%) | **196 (22%)** | 171 (19%) |
| Interstitial patches [3] | 58 (6%) | **45 (5%)** | 64 (7%) |
| **Model tissues with 50µ-diameter tubules** | | | |
| Tissue id | "1050" | "1550" | "2050" |
| Cap/tub ratio | 1.0 | 1.5 | 2.0 |
| Tub. ∅ [µ] | 50 | 50 | 50 |
| Cap. ∅ [µ] | 10 | 10 | 10 |
| Number of patches *per* type* (%*v*/*v*) | | | |
| Vascular patches | 28 (3%) | 42 (5%) | 58 (6%) |
| Epithelial patches | 533 (59%) | 534 (59%) | 535 (59%) |
| Luminal patches | 290 (32%) | 284 (32%) | 270 (30%) |
| Interstitial patches [3] | 49 (5%) | 40 (4%) | 37 (4%) |

[1], "effective" number of patches reduced to 900 for average tissue and patch-type $PO_2$ (124 border patches excluded); [2], rounded to the nearest integer; [3], %v/v calculated on the basis of the number of PTC patches.

## 2. House-keeping oxygen consumption in epithelial and other patches

### *2.1. Epithelial patches (HK-QO$_2$-EPI)*

In RCM, transport-related oxygen consumption (epithelial patches, TNa-QO$_2$-EPI) is dynamically calculated from reabsorptive sodium transport (TNa), via the $Na^+$/$K^+$-pump & Oxphos stoichiometry (SM1-Table S4, see equation 10). On the other hand, the calculation of epithelial house-keeping consumption (HK-QO$_2$-EPI) is carried out by partitioning whole-kidney consumption (Table S3). Step-wise calculations are explained below and summarized in SM2-Table S3 (intermediate calculations) and SM2-Table S4 (partitioning).

First step: transport-dependent versus house-keeping QO$_2$. Whole-kidney reference QO$_2$ is split into house-keeping (WK-HK-QO$_2$) and transport-linked consumption (WK-TNa-QO$_2$), using reference fractional basal consumption (frHKQO$_2$° = 0.25 ± 0.16, Table 4; see also [1]): this yields wk-HK-QO$_2$° = 1.45 mM-wk/min and WK-TNa-QO$_2$° = 4.34 mM-wk/min (SM2-Table S3, 2nd column). Recall that "mM-wk" refers to "Liter of kidney organ" (see Methods).

Second step: energetics of proximal and distal cortex versus medulla. Whole-kidney HK and TNa-QO$_2$ can be partioned into cortical and medullary terms, provided that fractional cortical (frQO$_2$-CTX) consumption is known. The latter can be evaluated on the basis of regional/segmental fractional reabsorption and associated TNa/QO$_2$ ratio; these values, however, are quite variable and/or uncertain because, in addition to being difficult to access, they depend on region/segment considered, and on various regulations and prevailing conditions (see [2–7]).

In normal condition, total kidney sodium reabsorption can be equated to filtered load, since fractional sodium excretion is less than 0.5% of filtered load [1]. On the other hand, (cortical + medullary) fractional reabsorption must amount to unity, while cortical reabsorption equals the sum of proximal and distal reabsorptions. On this basis, segmental/regional fractional reabsorptive costs can be estimated as the ratios of segmental fractional reabsorptions to their respective QO$_2$/TNa ratio

(nb: total transport cancels out in the ratio). Calculations are summarized in SM2-Table S3 below (bibliographical references for values reported in this table [2,8–10,4,11–13,6,14–16]):

(i) first line gives bibliographical values of "$Na^+$ fractional reabsorption" (see SM1-Table S5), and whole cortex (CTX) equals PT + DT reabsorptions; as such, this sum does not amount to 1.0, but 0.85 (see also physiological adjustments in Results);

(ii) third line presents bibliographical values of DT and MED fractional reabsorptions, and:

a. calculated CTX fractional reabsorption, 0.75 (frRCTX = 1 – frRMED),

b. deduced PT fractional reabsorption, 0.66 (frRCTX – frRDT);

(iii) the bottom section gives the segmental/regional fractional consumption (fractional reabsorption divided by $QO_2$/TNa, 1st line), and the percent of total (2nd line).

NB: we verified proper correspondence of these calculations with bibliographical whole-kidney data. Whole-kidney TNa/$QO_2$ is 21 ± 6 ($n$ = 7, $N$ = 5), which would correspond to a "fractional" $QO_2$ for CTX+MED of 0.0476 ± 0.015, whereas our value is 0.037 (SM2-Table S3, last column). This 30 % deviation is considered reasonable because of experimental uncertainties and because we ignored here the house-keeping contribution to overall consumption.

**SM2-Table S3.** Estimation of fractional segmental/regional reabsorptive costs.

| | CORTEX | | | MEDULLA | SUM |
|---|---|---|---|---|---|
| | **PT** | **DT** | **CTX** | **MED** | **CTX+MED** |
| **$Na^+$ fr. reabs.** | 0.51 (BV°) | 0.09 (BV°) | 0.60 (BV°) | 0.25 (BV°) | $0.85^{calc}$ |
| [range] | [0.27–0.65] | [0.03–.22] | [0.30–0.87] | [0.10–0.40] | - |
| **(frRx)** | **$(0.66)^{calc}$** | **$0.09^{bv}$** | **$(0.75)^{calc}$** | **$0.25^{bv}$** | **1.0** |
| **TNa/$QO_2$** | **27 ± 3** ($n$ = 3) | **17 ± 2** ($n$ = 2) | - | **34 ± 3** ($n$ = 3) | - |
| [range] | ~[24–30] | ~[15–18] | - | ~[30–36] | - |
| **Fractional $QO_2$** | **$0.0244^{calc}$** | **$0.0053^{calc}$** | **$0.0297^{calc}$** | **$0.0073^{calc}$** | **$0.0370^{bv}$** |
| **%(CTX + MED)** | **66** | **14** | **80** | **20** | **100** |

PT, DT, CTX, MED, proximal tubule, distal tubule, cortex, medulla; $Na^+$ fr. reabs., $Na^+$ fractional reabsorption (segmental, regional); BV° values, as in bibliographical analysis (Table 5); $^{calc}$, calculated value (see text); $^{bv}$, bibliographical value (see text); frRx, fractional reabsorption (for "x" segment/region); TNa/$QO_2$, ratio of $Na^+$ reabsorptive transport to $O_2$ consumption (mol/mol).

In fine, under normal conditions, the reference cortical fractional reabsorptive cost (frQO2-CTX°) amounts to ~0.7-0.8. We use 0.75, which leads to cortical transport (TNa-$QO_2$-CTX) and house-keeping costs (HK-$QO_2$-CTX) of 3.2 and 1.1 mM(wk)/min, respectively (SM2-Table S4, 3rd column). Such kidney-based $QO_2$ values are converted to cortex specific values using renal fractional cortical volume (frCV° = 0.66, SM1-Table S6), yielding TNa-$QO_2$-CTX° = 4.9 and HK-$QO_2$-CTX° = 1.6 mM-ctx/min (SM2—Table S4, fourth column), for a total specific cortical consumption of 6.5 mM-ctx/min.

Third step: assignment of house-keeping EPI specific consumption. Ignoring PTC consumption (see below for capillary consumption), we divide HK-$QO_2$-CTX (1.62 mM/min) by the model fractional volume of epithelial cells (0.66 for reference tissue; SM2-Table S2) to obtain reference specific HK-$QO_2$-EPI° = 2.42 mM/min (SM2-Table S4, last column). The influence of this independent parameter is addressed in the sensitivity analysis (see Results).

**SM2-Table S4.** Partition of reference whole-kidney consumption.

| | **WK Transport and Basal $QO_2$** | **Regional Transport and Basal $QO_2$** | **Cortex-specific Transport and Basal $QO_2$** | **Patch-type Specific TNa and HK-$QO_2$** |
|---|---|---|---|---|
| | Calculated with frHK-$QO_2$ ° (SM1-Table S5) | Calculated with fr$QO_2$-CTX (see text) | Calculated with frCV ° = 0.66 (Table 4a) | Calculated with EPI fractional volume [1] |
| **WK-$QO_2$** **5.8 ± 2.3** | WK-TNa-$QO_2$ **4.3** | TNa-$QO_2$-CTX **3.2** | **TNa-$QO_2$-CTX** **4.9** | **TNa-$QO_2$-EPI** **7.4** |
| | | TNa-$QO_2$-MED 1.1 | n.a. | n.a. |
| | WK-HK-$QO_2$ **1.4** | HK-$QO_2$-CTX **1.1** | **HK-$QO_2$-CTX** **1.6** | **HK-$QO_2$-EPI** [2] **1.9 ± 0.8** |
| | | HK-QO2-MED 0.4 | n.a. | n.a. |

| | | | | |
|---|---|---|---|---|
| mM-wk/min | mM-wk/min | mM-(ctx or med)/min | mM-ctx/min | mM-EPI/min |

[1], using HK-$QO_2$-PTC = 1.2, HK-$QO_2$-INT = 0.9; [2], see text, 1st to third steps, for detailed calculation and sources; wk, whole-kidney.

### *2.2. Other patches (HK-$QO_2$)*

In addition to EPI patches, two additional patch types consume oxygen in RCM, for "house-keeping" purposes, capillary (PTC) and interstitial (INT) patches.

Capillary patches: Despite their low specific consumption (1.2 mM/min, SM1-Table S5) and their limited volumic contribution (~7%-*v*/*v*) consumption by capillaries is considered. Literature is extremely uncertain (and experimentally rare), not to mention renal capillaries or dedicated Rat studies. Our bibliographical analysis swept capillaries, endothelial cells and micro-vessels/arteriolar wall, yielding a raw, wide-ranging, specific $QO_2$ of 16.5 ± 22.4 mM/min (range 0.3–51.0, [17]). Excluding extreme values (28 and 51 mM-ptc/min), we use HK-$QO_2$-PTC = 1.2 ± 1.1 mM-ptc/min. Of note, Vadapalli and coworkers, as cited above [17], later used the lower bound of their estimations (0.27 mM/min) in a mathematical model which specifically addressed capillaries [17,18]. HK-$QO_2$-PTC is scaled down to PTC fractional volume and the endothelial fractional volume (~0.23, an equivalent ~0.5 μm-thick crown, at Rc = 5 μm), yielding an effective $QO_2$ of ~0.020 mM-ptc/min. This seemingly low value was nevertheless kept and considered in RCM, first because of the experimental uncertainty, and second, for upcoming developments, that will target the distinction of endothelial vs epithelial cells aerobic/anaerobic metabolism and ATP production.

Interstitial patches: The renal interstitium is complex and heterogeneous [19], despite its low fractional volume (4–11%, see SM1-Table S3a). Interstitial cells include fibroblasts, monocytes/macrophages and neutrophils. Cortical fibroblast $QO_2$, a parameter rarely quantified, amounts to 0.5–2.0 mM/min [20]. Immune cells may consume ~0.2–4.0 mM/min, [21,22]). Currently, in RCM, we consider that one "undefined interstitial cell" resides per INT patch, with a specific $QO_2$ of 1.4 mM-INT/min.

## 3. Diffusion (programmed diffusion4 procedure)

### *3.1. Patch-type $DO_2$*

$O_2$ diffusion constant is a patch variable which conditions oxygen fluxes between patches. Thus, each patch type has its own value, but because of the 10 μm space discretization (patches), in the calculation of patch-to-patch diffusive (unidirectional) fluxes (Equation (16), Methods), we use the diffusion coefficient mean value of source and target patches.

SM1-Table 8 reports averages value for different biological tissues, in comparison with water and plasma. Accordingly, for lumina (LUM patches) we use "water/saline" bibliographical value ($2.8 \times 10^{-5}$ cm$^2$/s). For "interstitium" (INT patches), we also use the bibliographical value ($2.2 \times 10^{-5}$ cm$^2$/s); indeed, no more than it does for water, "extracellular matrix" does not represent a marked impediment to oxygen diffusion (e.g., [23]). For "capillaries", we use the following crude estimation: (fractional volume-) weighed mean of plasma (0.4), blood (0.4) and endothelial crown (0.2), i.e., $1.4 \times 10^{-5}$ cm$^2$/s (PTC patches). Finally, since for epithelial cells we could find any specific value, we attribute to EPI patches the $1.1 \times 10^{-5}$ cm$^2$/s value (averaged from "tissue", "intracellular" and "endothelial cells", SM1-Table S8). Note that $DO_2$ (EPI) is studied as an independent parameter in the sensitivity analysis (see Results).

### *3.2. Exchange surface areas*

Each patch has four contact surfaces with its neighbors, each one with a default area of 100 μm$^2$ (patch is 10 × 10 × 10 μm), through which $O_2$ diffuses. To account for cortical tubular (and/or capillary) cell-to-cell contacts geometry, this default value is multiplied by a factor, given in SM1-Table S3b. These factors are stored in NetLogo matrices, using the NetLogo Matrix extension. Capillary (PTC) contacts with epithelial cells (EPI) and with interstitium (INT) are set to depend on Rc, assuming PTC

are cylinders and distributing their lateral surface equally between the four neighbors. With respect to the standard 1000 $\mu m^3$ patch volume, the steric error amounts to –20% and +10% (at Rc = 5 and 6 µm, respectively). All other values related to epithelial cells are derived from morphometric determination in Rat kidney by Pfaller [24]. The brush-border membrane surface area (EPI-LUM interface) is 20, while the multiplying factor due to lateral inter-digitations between adjacent cells is 8 (SM1-Table 3a).

Because, in normal cortical tissue, peritubular capillary are "pressed" in between two or three tubules (e.g., see microphotographs in [19]), the contact surface area between PTC and tubules is further multiplied by a factor of 2.0, while the contact with interstitium is scaled down by a factor of 0.5 (SM1-Table S3b).

### *3.3. Coding of the diffusion procedure*

The physical equation for diffusion is given in *Methods*, but the actual, NetLogo implementation involves two additional factors. First, during each tick, the four unidirectional fluxes are calculated and stored for all 1024 patches; then fluxes are algebraically summed up for each patch, yielding net patch-to-patch oxygen diffusion throughout the whole model tissue. To ensure concurrent, from the same central pool, outgoing fluxes toward the four neighbors, a ¼ factor is introduced in (eq. 16). Second, in a discretized model as ours (10 µm and 1 ms), the diffusion equation assumes that all oxygen bound to diffuse does so along the whole center-to-center patch distance (10 µm), within one NetLogo tick (1 ms); however, in 2D diffusion, the (average) time required for $O_2$ to diffuse 10 µm is $t = 4 \times D/L^2$. As a consequence, the "diffusion time" toward each neighbor, which depends on adjacent patches average $DO_2$'s, is pre-calculated in the NetLogo setup procedure, and stored for each patch. Within the *diffusion4* procedure, it is then used in the diffusion equation (Equation (16)) as a factor to scale unidirectional fluxes ($J_{ij}$) to chosen tick duration (currently, 1 ms).

### *3.4. Verification of the oxygen diffusion procedure*

For simplicity, the verification of our diffusion routine was carried out with a simple model, featuring 11 × 11 identical patches ($DO_2 = 2.8 \times 10^{-5}$ $cm^2/s$), uniform 100 $\mu m^2$ exchange surface area, and one single, central, source patch (see Figure 1a). In all simulations, mass conservation was verified to be, in a 6-patches radius (60 µm), within $10^{-10}$ amoles at 1 ms and $10^{-2}$ amoles after 200 ms. Figure 1a presents a qualitative check, showing how $O_2$ from one instantaneous source spreads out in neighboring patches ($PO_2(t0)$ = 100 mmHg). The central patch empties below 0.1 mmHg in about 8 seconds, and $O_2$ reaches (> 0.1 mmHg) model borders, 150 µm in any direction, in about 3 seconds. With such an instantaneous source, the initial amount of oxygen distributes radially throughout the model, roughly emulating net diffusion (unidirectional fluxes of comparable magnitudes). If the source patch is continuous (e.g., 100 mmHg, constant), yielding dominant outwardly-directed oxygen fluxes, borders are reached in ~1.7 s; simulating this model with a physiological feeding $PO_2$ (45 mmHg, constant), they are reached in ~2.0 s (data not shown). Figure 1b shows the average diffusion distance (L) from the source in identical conditions (constant source, set at 45 mmHg) as a function of time. The theoretical relationship for 2D-Fick's diffusion is presented ($L^2 = 4Dt$; continuous line). The predicted curve is compared to simulated curves, obtained from the same simulation, using two different $PO_2$ thresholds to decide whether $O_2$ did reach the abscissa-given distance; the 0.1 and 0.01 mmHg thresholds respectively correspond to 0.125 and 0.0125 amol/patch (or µmol/L), equivalent to 75,250 and 7525 $O_2$ molecules per patch. Thus, diffusion-wise, our model exhibits an error of ~0.1 mmHg.

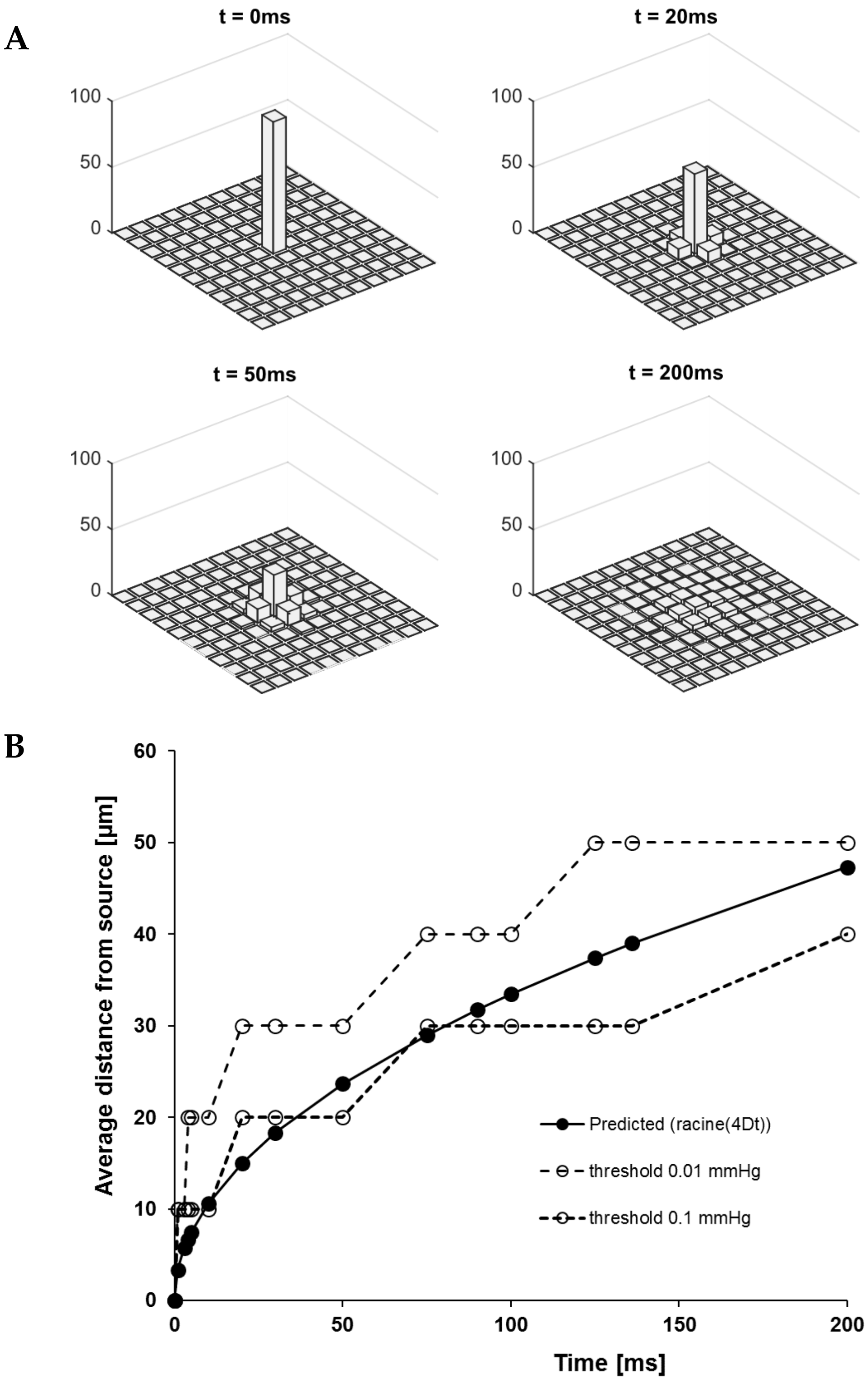


**SM2-Figure S1.** Verification and validation of the oxygen diffusion module. A "realistic" procedure was devised for oxygen diffusion. **A.** Verification of oxygen diffusion. **B.** Verification of oxygen - Diffusion distance.

Upper panel (A), qualitative verification. All patches are identical ($DO_2$ = 2.8 × $10^{-5}$ $cm^2/s$), initialized at $PO_2$ = 0 mmHg (exchange area, 100 $\mu m^2$), except for the source patch, initialized at 100 mmHg (instantaneous source). Patch $PO_2$ levels are represented on a 11 × 11 patch-grid (110 × 110 $\mu m^2$) centered on the source, at zero time and after 20, 50 and 200 ms. Oxygen progressively diffuses

from one patch to its neighbors and distributes around the source, until oxygen reaches model borders in ~3 s (150 μm) and the source empties (~8 s).

Lower panel (B) quantitative verification (constant source, 45 mmHg). In a plane, Fick's diffusion implies that the (squared) average diffusion distance is proportional to D and diffusion time (times *2.d*, where *d* is dimensionality, 2 in a plane). FThe fgure shows that the predicted curve ($L^2 = 4Dt$) falls in-between two simulated curves obtained using 0.1 (short-dotted line) and 0.01 mmHg (long-dotted line) oxygen "reaching" thresholds, at a given distance (vertical axis) within time (horizontal axis).

## 4. Hemoglobin/$O_2$ dynamics

Hemoglobin/$O_2$ interactions are usually studied at steady-state, as the so-called ODC (oxygen dissociation curve) which expresses Hb saturation ($SO_2$) vs $PO_2$ (or oxygen concentration). It is known that Hb deoxygenation from 100% saturation, or full saturation from zero, usually resolve in ca. 100-300ms, but can take anything from tens of milliseconds to seconds to complete, depending on the experimental model and conditions [25,26]; in vivo, such processes depend on capillary transit times allowance for oxygen capture (e.g., lung alveolar capillaries) or liberation (tissue capillaries), both lying in the 100–1000 ms (see [27]). Notwithstanding prevailing conditions ($O_2$ and Hb concentrations, capillary hemodynamics, as driven by tube diameter and local hematocrit, perfusion rate or residence time), Hb-related oxygen dynamic relaxation times appear (relatively) "slow" because Hb/$O_2$ on/off interactions are highly reversible. Gutierrez equations were fitted from human data ($P_{50}$ 27 mmHg). However, in the latter study, a wide range of $P_{50}$ was tested, including 37 mmHg [27]. Thus we confidently substituted the initial human value for the Rat's Hb specific one (37 mmHg). Indeed, this substitution did not destabilize RCM, nor modify its behavior (data not shown).

We specifically verified that dynamic equations from [27] do reproduce fast forwards/backwards rates "equilibration, within RCM and in isolation (Excel© sheet and Matlab/Simulink©): under all conditions tested, unidirectional binding and release rates become equal in less than ~10 ms. Further, we observed that in RCM Hb-related oxygen transients resolve in tens of milliseconds to several seconds, depending on conditions (see Results). Thus, although individual kinetic rates are fast and Hb/$O_2$ are "always" close to steady-state (for a discussion about this point, as well as about conditions and validity of Gutierrez equations, see [28,29]), we found it easier and more manageable to use equations from [27], because the (rest of the) whole model is *dynamic*, and because the very use of Hb/$O_2$ rate equations circumvented the otherwise slower and cumbersome (especially with NetLogo) requirement to iteratively solve an implicit equation (in order to determine the amount of $O_2$ released by $HbO_2$ when switching from one equilibrium point to another). Of final note, quantitative verifications (as above) and monitoring, showed that at model "steady-state" (for sst$PO_2$), hemoglobin/$O_2$ forward (binding) and reverse (release) processes do equilibrate, within 0.01 to 0.1 patch units (amol/(patch.ms)$^{-1}$) or ~0.01 to 0.1 mmHg, a numerical uncertainty ignored in model error estimations (see text).

## 5. Sensitivity analysis and I/O analysis

In Results, the LSA of model-independent parameters is detailed. Global SA (GSA, scanning of all parameter couples) was not performed. Nevertheless, exploratory GSA simulations were carried out (considering only steady-state tissue $PO_2$) to evaluate potential interactions, especially between most influential IP's, especially tubule length (LPT), fractional reabsorption (frPR), transcellular reabsorption fraction (frCTNa) and Oxphos ATP/$O_2$ ratio (P/O), in addition to selected, "minor" parameters such as epithelial HK consumption, or conjectural couples such as $DO_2$(EPI) and the BBM exchange surface factor. Briefly, we observed that:

(i) as expected, LPT and frPR do interact significantly, potentially generating each 0.5-2.0 mmHg error at most; however, taking into account that their mutual interactions are sign-opposite and partially cancel each other (75%) and that they can be expected to "vary" in the same direction (experimentally, the more tubule length is addressed, the higher the observed fractional reabsorption), the overall model error they would generate was estimated to ~0.4 mmHg; of note, this was the most influential couple observed in our exploratory GSA;

(ii) frPR, as expected too, did interact with P/O ratio, on the one hand, as well as, on the other hand, with an "uphill" parameter, FF (filtration fraction): in both cases, however, mutual influences were restricted to less than 1 mmHg (around BV°) and partially canceled out; the same observations were obtained with frCTNa and P/O ratio;

(iii) $DO_2$(EPI) and BBM factor, expected to modulate each other's (they both drive diffusion fluxes), only interact weakly (less than 0.2 mmHg); this was neglected;

(iv) epithelial HK-$QO_2$ did not interact noticeably with fractional reabsorption, and changed frReab-dependent sst$PO_2$ by less than 0.2 mmHg.

## Reference


1. Evans, R.G.; Harrop, G.K.; Ngo, J.P.; Ow, C.P.; O'Connor, P.M. Basal renal O2 consumption and the efficiency of $O_2$ utilization for Na+ reabsorption. *Am J Physiol Renal Physiol* **2014**, *306*, F551–F560.
2. Layton, A.T.; Vallon, V.; Edwards, A. Modeling oxygen consumption in the proximal tubule: Effects of NHE and SGLT2 inhibition. *Am J Physiol Renal Physiol* **2015**, *308*, F1343–F1357.
3. Moss, R.; Thomas, S.R. Hormonal regulation of salt and water excretion: A mathematical model of whole kidney function and pressure natriuresis. *Am J Physiol Renal Physiol* **2014**, *306*, F224–F248.
4. Deng, A.; Miracle, C.M.; Lortie, M.; Satriano, J.; Gabbai, F.B.; Munger, K. a; Thomson, S.C.; Blantz, R.C. Kidney oxygen consumption, carbonic anhydrase, and proton secretion. *American journal of physiology. Renal physiology* **2006**, *290*, F1009–F1015.
5. Deng, A.; Miracle, C.M.; Suarez, J.M.; Lortie, M.; Satriano, J.; Thomson, S.C.; Munger, K.A.; Blantz, R.C. Oxygen consumption in the kidney: Effects of nitric oxide synthase isoforms and angiotensin II. *Kidney Int* **2005**, *68*, 723–730.
6. Feraille, E.; Doucet, A. Sodium-potassium-adenosinetriphosphatase-dependent sodium transport in the kidney: Hormonal control. *Physiol Rev* **2001**, *81*, 345–418.
7. Frömter, E.; Rumrich, G.; Ullrich, K. Phenomenologic description of Na+, Cl− and HCO 3− absorption from proximal tubules of the rat kidney. *Pflügers Archiv* **1973**, *343*, 189–220.
8. Palmer, L.G.; Schnermann, J. Integrated control of Na transport along the nephron. *Clin J Am Soc Nephrol* **2015**, *10*, 676–687.
9. Satriano, J.; Sharma, K.; Blantz, R.C.; Deng, A. Induction of AMPK activity corrects early pathophysiological alterations in the subtotal nephrectomy model of chronic kidney disease. *Am J Physiol Renal Physiol* **2013**, *305*, F727–F733.
10. McDonough, A.; Thomson, S.C. Metabolic Basis of Solute Transport. In *Brenner & Rector'sThe Kidney 9th Edition*; Saunders, E., Ed.; 2012.
11. Varela, M.; Herrera, M.; Garvin, J.L. Inhibition of Na-K-ATPase in thick ascending limbs by NO depends on O2- and is diminished by a high-salt diet. *American Journal of Physiology . Renal Physiology* **2004**, *287*, 224–230.
12. Zhang, W.; Edwards, A. Oxygen transport across vasa recta in the renal medulla. *Am J Physiol Heart Circ Physiol* **2002**, *283*, H1042–H1055.
13. Welch, W.J.; Baumgartl, H.; Lubbers, D.; Wilcox, C.S. Nephron pO2 and renal oxygen usage in the hypertensive rat kidney. *Kidney Int* **2001**, *59*, 230–237.
14. Gullans L.J., S.R.& M. Coupling of energy to transport inproximal and distal nephron. In *The kidney: Physiology and pathophysiology*; 1992; pp. 445–482.
15. Hebert, S.C.; Reeves, W.B.; Molony, D. a; Andreoli, T.E. The medullary thick limb: Function and modulation of the single-effect multiplier. *Kidney international* **1987**, *31*, 580–589.
16. Kiil, F. Renal energy metabolism and regulation of sodium reabsorption. *Kidney Int* **1977**, *11*, 153–160.
17. Vadapalli, A.; Pittman, R.N.; Popel, A.S. Estimating oxygen transport resistance of the microvascular wall. *American Journal of Physiology-Heart and Circulatory Physiology* **2000**, *279*, H657–H671.
18. Vadapalli, A.; Goldman, D.; Popel, A.S. Calculations of oxygen transport by red blood cells and hemoglobin solutions in capillaries. *Artif Cells Blood Substit Immobil Biotechnol* **2002**, *30*, 157–188.
19. Aukland, K.; Bogusky, R.; Renkin, E. Renal cortical interstitium and fluid reabsorption by peritubular capillaries. *Am J Physiol Renal Physiol* **1994**, *266*, F175–F184.
20. Papandreou, I.; Cairns, R.A.; Fontana, L.; Lim, A.L.; Denko, N.C. HIF-1 mediates adaptation to hypoxia by actively downregulating mitochondrial oxygen consumption. *Cell Metabolism* **2006**, *3*, 187–197.

21. Chow, D.C.; Wenning, L. a; Miller, W.M.; Papoutsakis, E.T. Modeling pO(2) distributions in the bone marrow hematopoietic compartment. II. Modified Kroghian models. *Biophysical journal* **2001**, *81*, 685–696.
22. Reiss, M.; Roos, D. Differences in oxygen metabolism of phagocytosing monocytes and neutrophils. *J. Clin. Invest.* **1978**, *61*, 480–488.
23. Androjna, C.; Gatica, J.E.; Belovich, J.M.; Derwin, K. a Oxygen diffusion through natural extracellular matrices: Implications for estimating "critical thickness" values in tendon tissue engineering. *Tissue engineering. Part A* **2008**, *14*, 559–569.
24. Pfaller, W.; Seppi, T.; Ohno, A.; Giebisch, G.; Beck, F.X. Quantitative Morphology of Renal Cortical Structures during Compensatory Hypertrophy. *Nephron Experimental Nephrology* **1998**, *6*, 308–319.
25. Moll, W. The influence of hemoglobin diffusion on oxygen uptake and release by red cells. *Respir Physiol* **1968**, *6*, 1–15.
26. Gibson, Q.H.; Kreuzer, F.; Medan, E.; Roughton, F.J.W. The Kinetics of Human Haemoglobin in Solution and in the red cell at 37°C. *J Physiol* **1955**, *129*, 65–89.
27. Gutierrez, G. The rate of oxygen release and its effect on capillary O2 tension: A mathematical analysis. *Respir Physiol* **1986**, *63*, 79–96.
28. Sharan, M.; Selvakumar, S. A note on Gutierrez's kinetics model for oxygen delivery to tissue. *Biosystems* **1992**, *26*, 171–176.
29. Sharan, M.; Selvakumar, S. The effects of chemical kinetics on oxygen delivery to tissue. *Math Biosci* **1992**, *108*, 253–277.
